# Plasma membrane mediated GLUT10 mitochondrial targeting regulates intracellular ascorbic acid homeostasis

**DOI:** 10.1016/j.isci.2026.115891

**Published:** 2026-04-25

**Authors:** Anu Chirackal Jose, Yu-Wei Syu, Hao-Wen Lai, Ming-Yuan Tsai, Yi-Fan Jiang, Shao-Chun Hsu, Po-Yen Lin, Wan-Chen Huang, Wei-Chen Chu, Chi-Yu Fu, Yi-Ching Lee

**Affiliations:** 1 Institute of Cellular and Organismic Biology, Academia Sinica, Taipei 11529, Taiwan; 2 Molecular and Biological Agricultural Sciences Program, Taiwan International Graduate Program, Academia Sinica, Taipei 11529, Taiwan; 3 Graduate Institute of Biotechnology, National Chung-Hsing University, Taichung 40227, Taiwan; 4 Insititute of Molecular Medicine, National Tsing Hua University, Hsinchu 30013, Taiwan; 5 Graduate Institute of Molecular and Comparative Pathobiology, School of Veterinary Medicine, National Taiwan University, Taipei 10617, Taiwan; 6 Biotechnology Center, National Chung-Hsing University, Taichung 40227, Taiwan

**Keywords:** biochemistry, molecular biology, cell biology

## Abstract

Intracellular ascorbic acid (AA) regulation is essential for connective tissue homeostasis; however, the precise mechanisms governing AA homeostasis during cellular stress remain poorly understood. Here, we identify an oxidative stress-induced glucose transporter 10 (GLUT10) intracellular trafficking mechanism that regulates AA homeostasis via a noncanonical route from the endoplasmic reticulum (ER) to the plasma membrane (PM) and ultimately to mitochondria. This mechanism bridges the traditionally considered spatially and mechanistically distinct pathways of endomembrane system trafficking and mitochondria targeting. Using live-cell imaging and complementary biochemical approaches, we demonstrate that oxidative stress drives this redistribution. Increased PM localization of GLUT10 enhances the uptake of dehydroascorbic acid (DHA), the oxidized form of AA, thereby sustaining intracellular AA levels. The disruption of this trafficking pathway impairs AA homeostasis. Our findings reveal previously unrecognized localization of GLUT10 at the PM and endosomes and uncover endomembrane-mitochondria communication that maintains intracellular AA homeostasis and supports adaptation to oxidative stress.

## Highlights

• GLUT10 traffics via a unique route linking endomembranes and mitochondria
• Oxidative stress enhances GLUT10 transit from the ER to plasma membrane and endosomes
• RAB5-dependent endosomal sorting directs GLUT10 mitochondrial targeting
• Stress-induced GLUT10 trafficking sustains intracellular vitamin C homeostasis

## Graphical abstract

**Figure:**
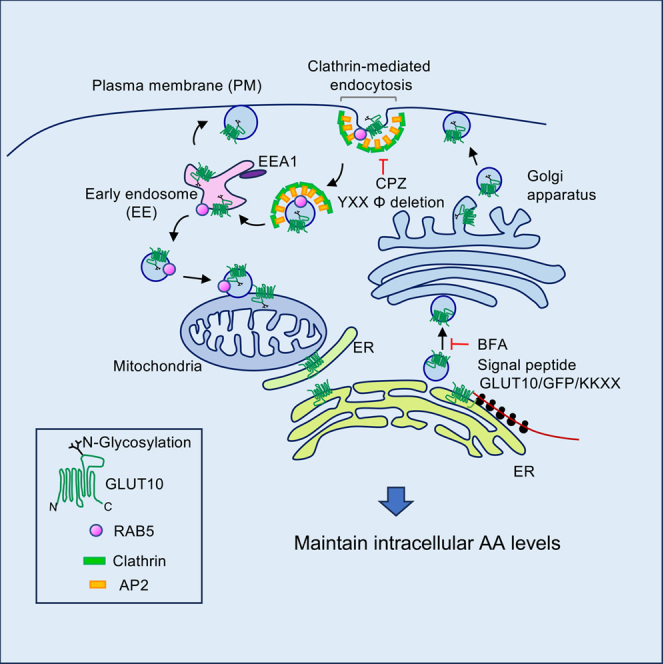

## Introduction

For a protein to function optimally, it must be targeted to the appropriate subcellular location within the proper physiological context. Many proteins play functional roles in cellular compartments distant from where they are synthesized, and targeting them to the proper location typically involves intricate mechanisms dependent on sorting signals in their amino acid sequences.^1^ It is widely accepted that the mechanisms that target proteins to mitochondria are largely distinct from those that target other proteins to the endomembrane system. In general, nuclear-encoded mitochondrial proteins are synthesized in the cytosol and rely on mitochondrial targeting sequences to enter the mitochondrial compartments. Meanwhile, proteins destined for secretion or the endomembrane system [i.e., endoplasmic reticulum (ER), Golgi, lysosomes, and plasma membrane (PM)] are synthesized on ribosomes associated with the rough ER.^2^ Interestingly, recent studies have shown that some proteins can be dually targeted to both mitochondria and other compartments,^3^ and several nuclear-encoded mitochondrial proteins undergo N-glycosylation,^4^^,^^5^^,^^6^ a process that typically occurs in the ER and Golgi.^7^ Thus, the targeting of proteins to mitochondria may be more complex and varied than previously expected.

It is well accepted that mitochondria have many functions and communicate with other organelles to maintain cellular homeostasis. For instance, direct interactions between mitochondria and organelles such as the ER, nucleus, and peroxisomes have been implicated in the regulation of calcium homeostasis and lipid metabolism.^8^ Additionally, the interaction and crosstalk between the ER and mitochondria have recently received significant attention.^9^ Interactions between endosomes and mitochondria have also been shown to facilitate intracellular iron transfer in different cell types.^10^^,^^11^ Although intricate networks of inter-organelle communication that control cellular homeostasis and adaptation are beginning to be identified, the complete landscape of functional organelle interactions remains to be fully delineated.

The solute carrier transporter family (SLCs) is characterized by proteins with multiple transmembrane domains that function to mediate metabolite movement across all cellular membranes. As such, proper subcellular localization of SLCs is crucial for regulating metabolic flux and maintaining cellular physiological states. However, the mechanisms by which environmental cues regulate SLC subcellular localization to maintain metabolic homeostasis remain largely unknown. Functional deficiencies in various SLCs have been linked to the etiology of numerous diseases,^12^ providing a valuable platform for studying their function and regulation. Loss-of-function mutations in the *SLC2A10* gene, which encodes glucose transporter 10 (GLUT10), lead to arterial tortuosity syndrome (ATS), a rare connective tissue disease that affects the development and maintenance of connective tissues.^13^ Our studies have shown that GLUT10 is highly expressed in aortic smooth muscle cells (ASMCs) of major arteries, which are the primary site of lethal complications in patients with ATS.^14^ In ASMCs, GLUT10 is located in both ER and mitochondria, where it facilitates the transport of dehydroascorbic acid (DHA), the oxidized form of ascorbic acid (AA; vitamin C).^14^^,^^15^^,^^16^ Overexpression of GLUT10 in ASMCs significantly increases DHA uptake and maintains intracellular and mitochondrial AA levels, redox balance, and mitochondrial function. Furthermore, AA serves as an enzyme cofactor in the hydroxylation of proline and lysine, which is essential for stabilizing collagen structure within connective tissues. Thus, defects in GLUT10 function impair connective tissue development and integrity, driving ATS pathogenesis.^14^^,^^15^

Intriguingly, our previous work demonstrated that under aging and oxidative stress conditions, elevated intracellular and mitochondrial ROS levels increase GLUT10 colocalization with mitochondria in ASMCs, which is important for enhancing DHA uptake and maintaining intracellular AA levels.^15^ These findings highlight the importance of proper GLUT10 localization in both physiological and pathological contexts. This raises a key question: How does GLUT10 traffic to the mitochondria, and how does this trafficking influence its function? Our findings reveal a surprising trafficking pathway in which GLUT10 is directed from the ER to mitochondria via the PM and endosome-mediated pathway. GLUT10 became N-glycosylated in the ER and Golgi before being targeted to the PM. The N-glycosylation may facilitate early endosomal sorting and subsequent delivery to the mitochondria. Notably, stress conditions further enhance this trafficking. Importantly, PM localization of GLUT10 promotes DHA uptake and contributes to the maintenance of intracellular AA levels. This pathway reveals previously unrecognized localization of GLUT10 at the PM and endosomal vesicles and suggests a dynamic redistribution of GLUT10 as part of cellular adaptation. Overall, our study establishes a link between protein subcellular localization and the regulation of intracellular AA levels, highlighting the importance of this mechanism for cellular adaptation to environmental stress.

## Results

### GLUT10 transits from the ER to mitochondria via vesicles

We previously demonstrated that in primary human ASMCs (hASMCs), aging and oxidative stress elevate intracellular ROS, thereby increasing endogenous GLUT10 targeting to mitochondria, which is critical for maintaining intracellular AA levels and arterial walls integrity.^15^ Consistent with previous findings, our confocal imaging experiments on GLUT10/GFP-expressing ASMCs showed that the protein colocalized with both ER and mitochondria (Figures S1A and S1B). These findings were further supported by subcellular fractionation and immunoblotting in both ER- and mitochondria-enriched fractions in GLUT10/GFP-expressing ASMCs (Figures S1C and S1D). Importantly, we further confirmed the presence of endogenous GLUT10 in mitochondrial- and ER-enriched fractions from 293T cells (Figure S1E). Consequently, both ASMCs and 293T cells were used in subsequent experiments.

**Figure 1:**
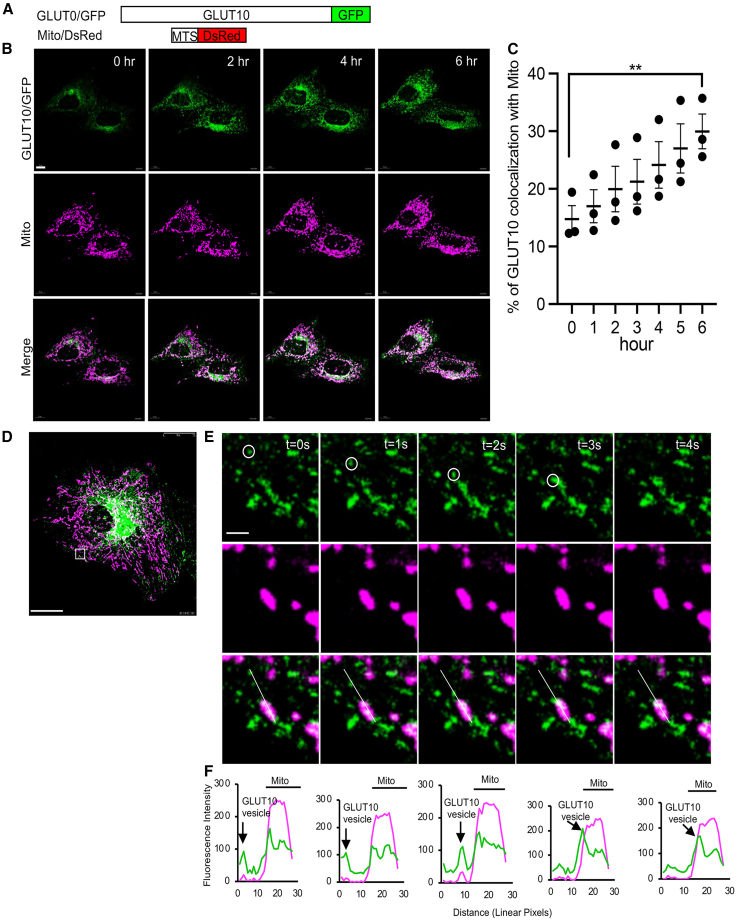
GLUT10-containing vesicles target mitochondria (A) Schematic of GLUT10/GFP and Mito/dsRed fusion proteins. GLUT10/GFP was generated by fusion GFP to the C-terminus of GLUT10 (GLUT10/GFP). Mito/dsRed was generated by fusing dsRed to the C-terminus of a mitochondrial targeting sequence (MTS) derived from human *COX8A*. (B) Representative confocal images show the colocalization of GLUT10/GFP and mitochondria at the indicated time points. A10 cells co-expressing GLUT10/GFP and Mito/dsRed were treated with 100 μM H_2_O_2_; live-cell imaging was performed every hour for 6 h. Scale bars, 10 μm. (C) Quantification of mitochondrial colocalization. Percentage of GLUT10/GFP signals colocalized with Mito/dsRed calculated from three cells across three independent experiments. Data represent the mean ± SEM. Statistical significance was determined using two-tailed Student’s *t* test. ∗*p* < 0.05. (D) Merged confocal image of live A10 cells co-expressing GLUT10/GFP and Mito/dsRed. Scale bars, 25 μm. (E) Time-lapse confocal images show the dynamic colocalization of GLUT10/GFP and Mito/dsRed over 0–4 s from the region enlarged in (D). Scale bars, 1 μm. (F) Fluorescence intensity profiles along the line drawn in E, shown for each time point from 0 to 4 s. B, D, and E, *Green,* GLUT10/GFP; *magenta*, Mito/dsRed; *white,* merged.

To further investigate the dynamics of GLUT10 subcellular localization under stress conditions, we used live-cell imaging to track GLUT10 intracellular trafficking in ASMCs co-transfected with GLUT10/GFP and Mito/dsRed, a mitochondrial marker construct containing a mitochondrial targeting sequence (Figure 1A). Under basal conditions, GLUT10 predominantly localized to the perinuclear region, consistent with ER localization (Figures 1B and S1B). Upon H_2_O_2_ treatment, GLUT10 underwent a dynamic redistribution throughout the cell, with a marked increase in mitochondrial colocalization (Figure 1B and Video S1). Quantitative analysis revealed that the mitochondrial colocalization of GLUT10 significantly increased from approximately 10-20% to 25–40% following 6 h of H_2_O_2_ treatment (Figure 1C), indicating a stress-induced translocation. In contrast to GLUT10, although GLUT1 has previously been reported to localize to mitochondria,^17^ we detected only minimal mitochondrial colocalization of GLUT1, and H_2_O_2_ treatment did not increase its mitochondrial localization (Figure S2 and Video S2), indicating that the oxidative stress-induced increase in mitochondrial localization is a distinctive feature of GLUT10. High-resolution live-cell images at 1-s intervals revealed dynamic trafficking of GLUT10-containing vesicles, with some vesicles moving toward and making close contact with mitochondria (Figures 1D–1F, Videos S3 and S4), supporting the notion that oxidative stress promotes targeted GLUT10 redistribution toward mitochondria.

Video S1. Live time-lapse confocal imaging illustrating GLUT10/GFP targeting to mitochondria A10 cells co-expressing both GLUT10/GFP and Mito/dsRed. Cells were treated with 100 μM H_2_O_2_, and imaging was performed every 1 h for 6 h. Scale bars, 15 μm.

Video S2. Live time-lapse confocal imaging illustrating GLUT1/GFP targeting to mitochondria A10 cells co-expressing both GLUT1/GFP and Mito/dsRed. Cells were treated with 100 μM H_2_O_2_, and imaging was performed every 1 h for 6 h. Scale bars, 10 μm.

Video S3. Live time-lapse confocal imaging illustrating GLUT10/GFP-containing vesicles interacting with mitochondria A10 cells expressing both GLUT10/GFP and Mito/dsRed, Cells were treated with 100 μM H_2_O_2_; imaging was performed every 1 s for 171 s. Scale bars, 15 μm.

Video S4. Enlarged live time-lapse confocal imaging illustrating GLUT10/GFP-containing vesicles approaching, making direct contact with, and merging with mitochondria A10 cells expressing both GLUT10/GFP and Mito/dsRed, Cells were treated with 100 μM H_2_O_2_; imaging was performed every 1 s for 171 s. Scale bars, 5 μm.

We next wanted to examine the dynamics of GLUT10-containing vesicle targeting to mitochondria, so we analyzed the GLUT10-containing particle movements in H_2_O_2_-treated cells. The particles were tracked at 1-s intervals for 30 s, and we observed linear trajectories of GLUT10 vesicles departing from the perinuclear region toward the cell periphery (Figure S3A). This pattern of movement was indicative of regulated GLUT10 vesicle trafficking. Our overall analysis of 429 trajectories allowed us to identify two types of motion, including stationary (320 trajectories with track displacements <0.67 μm) and dynamic (109 trajectories with displacements >0.67 μm) (Figures S3B and S3C). Speed analysis showed that the GLUT10 vesicles slowed upon encountering mitochondria and then merged with stationary tracks (Figures S3D and S3E). These observations led us to conclude that the GLUT10-containing vesicles come into close contact with mitochondria, suggesting a role for vesicle-mediated trafficking in targeting GLUT10 to mitochondrial compartments.

### GLUT10 localizes to the endomembrane system and mitochondria

**Figure 2:**
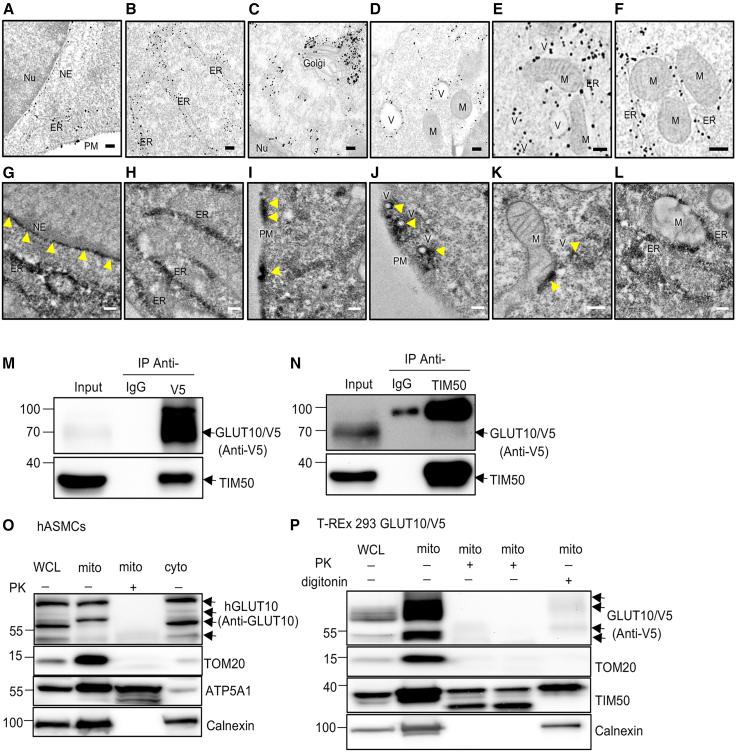
GLUT10 localizes to the endomembrane system and mitochondria (A–F) TEM images of ultrathin sections from GLUT10/GFP-expressing 293T cells. GFP was labeled with 1.4 nm nanogold-conjugated antibodies. GLUT10/GFP signal was detected in ER (A, B, E, and F), plasma membrane (PM) and nuclear envelope (NE) (A), Golgi (C), mitochondria (M) (D–F), vesicles (V) (D and E), and ER-mitochondria contacts (F). Scale bars, 200 nm. For A–F, images were adjusted for brightness and contrast uniformly, and no region-specific or selective modifications were performed. The regions shown in each panel represent enlarged views selected from the original images. (G–L) TEM images of MOVAS cells expressing GLUT10/APEX2. Enhanced electron contrast indicates GLUT10/APEX2 localization in ER (G, H, and L), nuclear envelope (NE) (G), plasma membrane (PM) (I), vesicles (V) (J), mitochondria (K), and ER-mitochondria contacts (L). Scale bars, 100 nm. (M and N) Detection of a direct interaction between GLUT10 and mitochondrial inner membrane protein TIM50 by immunoprecipitation (IP) and immunoblots of specific antibodies in T-REx-293 cells expressing GLUT10/V5. (M) Immunoblot analysis of GLUT10/V5 and TIM50 in whole-cell lysates from T-REx-293 cells expressing GLUT10/V5 before IP (Input) or after IP using either control IgG or anti-V5 antibodies. (N) Immunoblot analysis of GLUT10/V5 and TIM50 in whole-cell lysates from T-REx-293 cells expressing GLUT10/V5 before IP (Input) or after IP using either IgG or anti-TIM50 antibodies. (O and P) Localization of GLUT10 on the mitochondrial outer membrane (MOM) was analyzed by immunoblotting of mitochondria-enriched fractions coupled with proteinase K or digitonin treatment. (O) Immunoblots of endogenous GLUT10, ATP5A1 (mitochondrial inner membrane, MIM), TOM20 (MOM), and Calnexin (ER membrane) in whole cell lysate (WCL), mitochondrial-enriched fractions (mito), cytosolic fractions (cyto) from hASMCs treated with (+) or without (−) proteinase K (PK). (P) Immunoblots of GLUT10/V5 (anti-V5 antibodies), TIM50 (mitochondrial inner membrane, MIM), TOM20 (MOM), and Calnexin in whole cell lysate (WCL) and mitochondrial-enriched fractions (mito) from GLUT10/V5-expressing T-REx 293 cells treated with (+) or without (−) proteinase K (PK) or digitonin.

Since our initial live-cell trafficking data revealed a broad intracellular distribution of GLUT10 and suggested a vesicular trafficking route from the ER to mitochondria, we sought to investigate the ultrastructural details of GLUT10 localization. Transmission electron microscopy (TEM) with immunogold labeling showed gold particles localized to the ER, mitochondria, PM, nuclear envelope (NE), Golgi complex, and vesicle structures, with particularly high signal intensity on vesicles and ER membranes (Figures 2A–2F). This high density of labeling intracellular membranes corroborated the confocal microscopy results. To validate these findings using an independent experimental approach, we utilized enhanced ascorbate peroxidase 2 (APEX2)-tagged GLUT10 (GLUT10/APEX2) and examined its localization by TEM. As APEX2 catalyzes diaminobenzidine (DAB) polymerization to provide EM contrast,^18^ we could directly visualize GLUT10/APEX2 subcellular localization (Figures S4A–S4C). Compared to standard osmium fixated controls (Figure S4D), APEX2-only controls (Figure S4E), and negative controls without DAB staining (Figure S4F), we observed specific DAB signals corresponding to GLUT10/APEX2 on the ER, PM, endocytosed vesicles, and mitochondria (Figures 2G–2L and Video S5).

Video S5. Electron tomogram reconstruction of MOVAS cells expressing GLUT10/APEX2, illustrating the endocytosed vesicles Cells were stained with DAB. EM images show strong EM contrast signals indicating the localization of GLUT10/APEX2.

Next, we wanted to better understand the mitochondrial localization of GLUT10, so we examined its interactions with mitochondrial proteins. To identify GLUT10-interacting proteins, we conducted co-immunoprecipitation (co-IP) followed by mass spectrometry (MS) on GLUT10/V5-expressing T-REx-293 cells. Using this strategy, we were able to identify several mitochondrial proteins that associated with GLUT10 (Figure S5A and Table S1), such as import receptor subunits (TOM40, TOM70, TIM50), mitochondrial ATP synthase subunits (ATP5C1, ATP5A1 and ATPAF1), and mitochondrial matrix proteins [NAD (+)-dependent isocitrate dehydrogenases (IDH3B)]. Among these proteins, the most abundant mitochondrial protein associated with GLUT10/V5 was the mitochondrial inner membrane protein, TIM50 (Figure S5A). The interaction between GLUT10 and TIM50 was further confirmed by co-IP using an anti-V5 antibody, and reciprocal co-IP with an anti-TIM50 antibody, followed by immunoblotting (Figures 2M and 2N). We also explored the potential direct interaction between GLUT10/V5 and a mitochondrial matrix protein, IDH3B, which was identified from the initial co-IP/MS assay. However, no direct interaction was observed for these two proteins (Figure S5B), underscoring the specificity of the identified association of GLUT10 with TIM50 and further supporting the conclusion that GLUT10 is indeed localized to mitochondria.

To clarify whether GLUT10 localizes to the mitochondrial outer membrane or the mitochondrial inner membrane, both of which could potentially allow interaction with TIM50, we probed the sub-mitochondrial localization of GLUT10 using a proteinase K and digitonin protection assay.^19^ Mitochondria purified from hASMCs or GLUT10/V5-expressing T-REx-293 cells were subjected to proteinase K digestion, which degraded the cytosol-exposed outer membrane proteins. As expected, the known outer membrane protein TOM20 was sensitive to proteinase K digestion, while inner membrane proteins ATP5A1 and TIM50 were resistant (Figures 2O and 2P). Interestingly, GLUT10 was sensitive to proteinase K treatment, suggesting it is most likely localized to the outer membrane (Figures 2O and 2P). Next, we wanted to confirm the localization of GLUT10 to the outer membrane and exclude the potential interference of ER contamination on the proteinase K assay results. To do so, mitochondria were purified from cells and treated with digitonin at a concentration that selectively solubilizes the mitochondrial outer membrane but preserves the integrity of the inner membrane (Figure 2P). After digitonin treatment, both TOM20 and GLUT10 levels were significantly reduced, whereas the inner membrane protein TIM50 remained unaffected (Figure 2P), suggesting the conclusion that GLUT10 is localized to the mitochondrial outer membrane. Taken together, our findings to this point indicate that GLUT10 is localized to both the endomembrane system and the outer membrane of mitochondria.

### ER-Golgi derived N-glycosylated GLUT10 is detected in mitochondria

**Figure 3:**
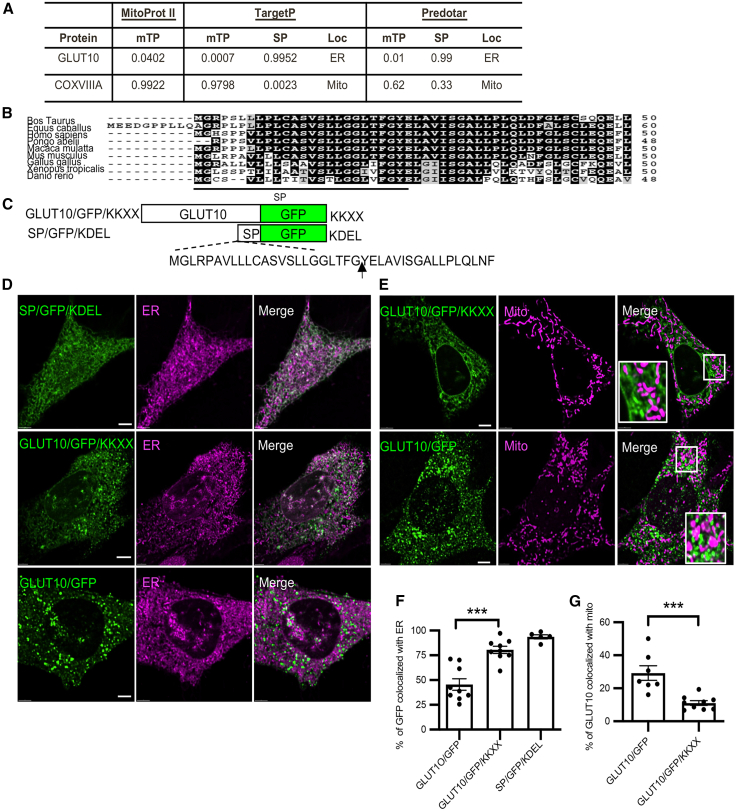
GLUT10 retention in the ER reduces its mitochondrial targeting (A) Prediction of mitochondrial-targeting signals (mTP), signal peptide (SP), and subcellular localization (Loc) of mouse GLUT10 by Mitoprot II, TargetP, and Predotar analyses. CoxVIIIA, human cytochrome oxidase subunit VIIIA, a mitochondrial transmembrane protein, is shown as a positive control. (B) N-terminal amino acid alignments of GLUT10 from several species using CLUSTAL W. Black shading indicates fully conserved residues; gray shading indicates residues within the same conservation group. SP denotes ER signal peptide predicted by SignalP. (C) Schematic diagram of GFP fusion constructs: GLUT10/GFP/KKXX and SP/GFP/KDEL. Arrow indicates the predicted SP cleavage site. KKXX and KDEL serve as ER retention signals for membrane proteins and soluble proteins, respectively. (D) Representative confocal images of MOVAS cells expressing the indicated GFP fusion proteins stained with ER Tracker Red. *Green,* GFP; *magenta*, ER Tracker Red; *white,* merged. Scale bars, 10 μm. (E) Representative confocal images of MOVAS cells expressing the indicated GFP fusion proteins stained with MitoTracker Red. *Green,* GFP; *magenta*, MitoTracker Red; *white,* merged. Scale bars, 10 μm. (F) Quantification of the percentage of GFP fusion proteins colocalized with ER, as shown in D. Data represent the mean ± SEM, n = 5–10 cells from three independent experiments. (G) Quantification of the percentage of GFP fusion proteins colocalized with mitochondria, as shown in E. Data represent the mean ± SEM, n = 7–9 cells from three independent experiments. Statistical comparisons were made with a two-tailed Student’s *t* test. ∗∗∗*p* < 0.001.

To identify the trafficking pathways that regulate GLUT10 intracellular distribution, we searched for functional targeting motifs within the GLUT10 sequence. By doing so, we identified a typical evolutionarily conserved N-terminal signal peptide (SP) for ER targeting (Figures 3A and 3B); however, no consensus mitochondrial targeting signal was found (Figure 3A). To test the functionality of the SP, we fused the SP of GLUT10 to a GFP tagged with an ER-retention signal (KDEL) (SP/GFP/KDEL) (Figure 3C). The SP directed GFP to the ER, and the KDEL signal restrained the protein in the ER (Figures 3D and 3F), confirming that the SP of GLUT10 is functional and sufficient for ER targeting.

To further investigate whether retained GLUT10 in the ER might affect its mitochondrial trafficking, a transmembrane protein ER retention signal, di-lysine (KKXX) motif^20^ was fused to the C-terminus of GLUT10/GFP (GLUT10/GFP/KKXX) (Figure 3C). The GLUT10/GFP/KKXX protein was retained in the ER and exhibited reduced mitochondrial trafficking under H_2_O_2_ treatment (Figures 3D–3G), suggesting that the retention of GLUT10 in the ER can impair its mitochondrial targeting.

**Figure 4:**
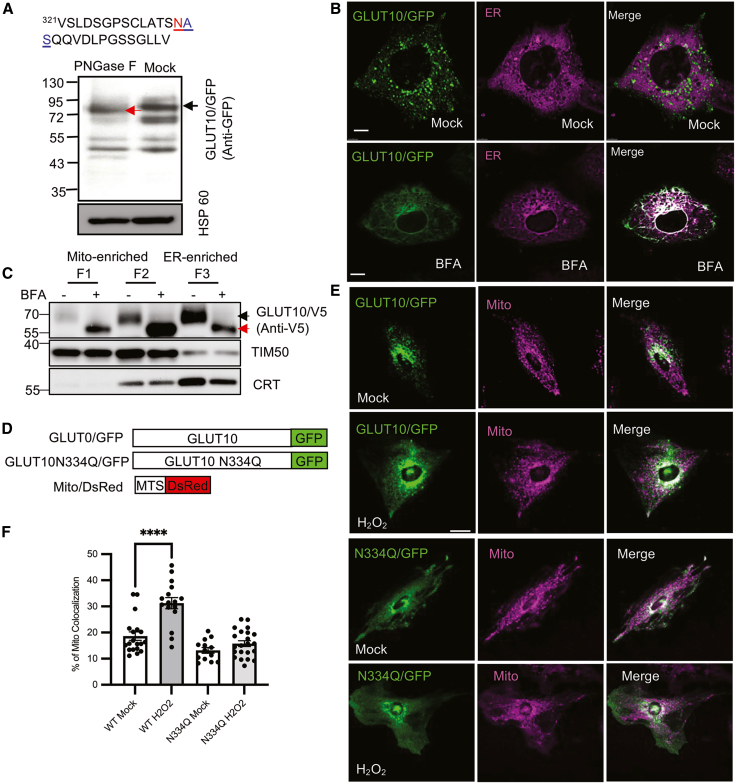
Mitochondrial GLUT10 is N-glycosylated (A) The N-glycosylation site (N-X-S, underlined triplet) in mouse GLUT10 was predicted with NetNglyc1.0. The asparagine (N) predicted to be N-glycosylated is highlighted in red. Immunoblot analysis showed a molecular weight shift of GLUT10/GFP in mitochondrial-enriched fractions from GLUT10/GFP-expressing A10 cells treated with PNGaseF (indicated by black and red arrows). HSP60, Heat shock protein 60, a mitochondrial marker. Brightness and contrast were adjusted across the entire WB image for better visualization of bands. (B) Representative confocal images of GLUT10/GFP colocalized with ER in A10 cells expressing GLUT10/GFP treated with or without 5 μM BFA for 1 h; ER was stained with ER Tracker Red. *Green,* GFP; *magenta*, ER Tracker Red; *white,* merged. Scale bars, 10 μm. (C) Immunoblots to detect molecular weight changes of GLUT10/V5 in subcellular fractions. GLUT10/V5 T-REx-293 cells were induced with TET for 12 h. After 4 h of induction, cells were treated with or without 3.56 μM BFA for 4 h. BFA was then removed, and induction continued for another 4 h. Mitochondria-enriched fractions (F1 and F2) and ER-enriched fractions were subjected to IB using V5 for GLUT10/V5; TIM50, mitochondrial marker; CRT, calreticulin, ER marker. (D) Diagram shows GLUT10/GFP, GLUT10 N334Q mutant fused to GFP (GLUT10 N334Q/GFP), and Mito/dsRed fusion protein. (E) Representative confocal images of A10 cells expressing GLUT10/GFP or GLUT10 N334Q/GFP and Mito/dsRed treated without (Mock) or with 100 μM H_2_O_2_ for 12 h. *Green,* GFP; *magenta*, Mito/dsRed; *white,* merged. Scale bar, 25 μm. (F) Quantification of the percentage of GLUT10/GFP (WT) or GLUT10 N334Q/GFP (N334Q) colocalized with mitochondria, as in B. Data represent the mean ± SEM, n = total of 13–22 cells from three independent experiments. Statistical comparisons were made with a two-tailed Student’s *t* test. ∗∗∗∗*p* < 0.0001.

GLUT10 contains a predicted N-linked glycosylation site (Figure 4A), and we have observed distinct molecular weights for GLUT10 in the mitochondrial- and ER-enriched fractions in both GLUT10/GFP expressing cells and endogenous contexts (Figures S1D and S1E), suggesting post-translational modifications. Since N-linked glycosylation typically occurs in the ER and Golgi,^21^ we hypothesize that mitochondrial GLUT10 is trafficked through the ER-Golgi pathway and undergoes N-glycosylation. To test this, mitochondria-enriched fractions from GLUT10/GFP expressing ASMCs were treated with N-glycosidase F (PNGase F), which removes N-linked glycans.^22^ This treatment reduced the molecular weight of GLUT10/GFP (Figure 4A). Since the GFP moiety is not glycosylated,^23^ these results indicate that mitochondrial GLUT10 is indeed N-glycosylated.

To confirm that ER to Golgi translocation is important for GLUT10 glycosylation and targeting to mitochondria, we treated cells with brefeldin A (BFA), an inhibitor of vesicle formation and transport between the ER and Golgi.^24^ BFA treatment resulted in GLUT10 accumulation in the ER (Figure 4B) and reduced its molecular weight in both mitochondrial and ER-enriched fractions (Figure 4C), in line with the expected loss of N-glycosylation. Paradoxically, upon BFA treatment, the intensity of the lower molecular weight form of GLUT10 increased in F1 (mitochondria-enriched fraction) and F2 (mitochondria- and MAM-enriched) fractions, while it decreased in F3 (ER-enriched) fraction. Although the GLUT10/GFP images displayed a reticular network resembling the ER pattern under BFA treatment, the shift in fractionation profiles can be explained by previous reports that BFA enhances ER-mitochondria contacts (MAMs),^25^ which are enriched in the F2 fraction, as indicated by the presence of both ER and mitochondrial markers. This increased ER-mitochondria interaction likely facilitates the redistribution of immature, ER-retained GLUT10 into the mitochondria- and MAM-enriched fractions, despite its lower molecular weight, and images confirm its retention in the ER.

Since N-glycosylation is a well-established regulator of intracellular protein trafficking,^26^ so we next tested the effect of mutating the predicted N-linked glycosylation site from asparagine (N) to glutamine (Q) in GLUT10 (GLUT10-N334Q) (Figure 4D). The results showed that the GLUT10-N334Q mutation significantly reduced its mitochondrial targeting upon H_2_O_2_ treatment (Figures 4E and 4F). This suggests that GLUT10 N-glycosylation is essential for its mitochondrial targeting.

In conclusion, these findings suggest that GLUT10 is transported through the ER-Golgi system, where it undergoes N-glycosylation before being targeted to mitochondria.

### YXXΦ motif mediates clathrin-dependent GLUT10 endocytosis from the PM

**Figure 5:**
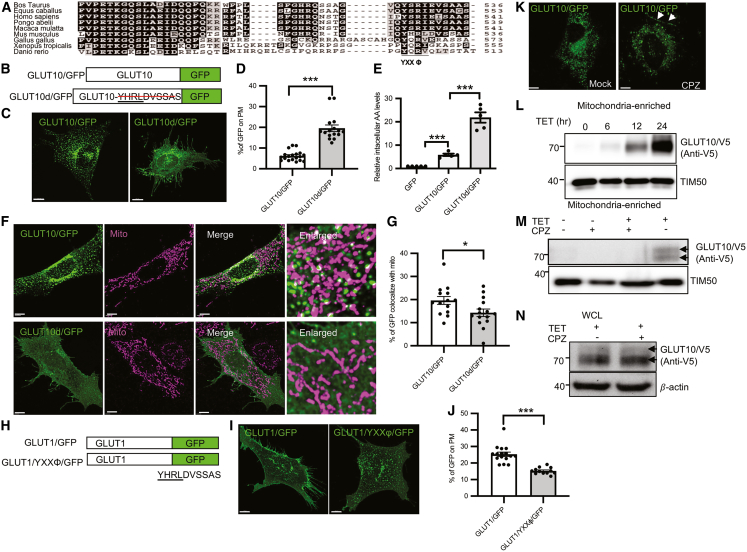
YXXΦ motif and clathrin mediate GLUT10 plasma membrane endocytosis and mitochondrial targeting (A) Amino acid alignments of the GLUT10 C-terminus from several species analyzed by the CLUSTAL W program. The YXXΦ motif is underlined. Residues in black shading are conserved, while gray shading indicates amino acids belonging to the same conservation group. (B) GLUT10/GFP and YXXΦ motif-deleted GLUT10/GFP (GLUT10d/GFP) fusion proteins. (C) Confocal images of MOVAS cells expressing GLUT10/GFP or GLUT10d/GFP. Scale bars, 10 μm. (D) Quantification of the relative intensity of plasma membrane-localized GFP, as in C. A custom ImageJ macro was used as described in Supplementary materials and Figure S8. Data are shown as mean ± SEM, n = total 16–19 cells from 3 independent experiments. (E) Quantification of the DHA uptake. A10 cells expressing GFP, GLUT10/GFP, or GLUT10d/GFP were incubated with 5 mM DHA for 30 min, and intracellular AA levels were measured by HPLC and presented as relative levels compared to cells expressing GFP control. Data are shown as mean ± SEM from 5 independent experiments. (F) Confocal images of MOVAS cells expressing GLUT10/GFP or GLUT10d/GFP stained with MitoTracker Red. *Green,* GFP; *magenta*, MitoTracker Red; *white,* merged. Scale bars, 5 μm. (G) Quantification of the percentages of GLUT10/GFP and GLUT10d/GFP colocalized with MitoTracker, as in F. Data represent the mean ± SEM, *n* = 14–15 cells from 3 independent experiments. (H) GLUT1/GFP and GLUT1-YXXΦ/GFP fusion proteins. (I) Confocal images of MOVAS cells expressing GLUT1/GFP or GLUT1-YXXΦ/GFP. Scale bars, 10 μm. (J) Quantification of the relative intensity of plasma membrane GFP, as in I. Images were analyzed using a custom ImageJ macro described in Supplementary materials and Figure S13. Data are shown as mean ± SEM, n = total 12–16 cells from 3 independent experiments. (K) Confocal images of GLUT10/GFP-expressing A10 cells treated with or without 20 μM CPZ for 6 h. Arrows indicate plasma membrane-localized GLUT10/GFP. Scale bars, 10 μm. (L) Immunoblots of GLUT10/V5 levels in mitochondria-enriched fractions from T-REx-293 cells with induced GLUT10/V5 expression at the indicated time points. IB, V5 for GLUT10/V5, TIM50, mitochondrial marker. (M and N) Immunoblots of GLUT10/V5 levels in (M) mitochondria-enriched fractions and in (N) total protein lysates of T-REx-293 cells pretreated with 20 μM CPZ for 1 h before the induction of GLUT10/V5 expression for 6 h. IB, V5 for GLUT10/V5, TIM50, mitochondrial marker, and beta-actin served as a total protein loading control. Statistical comparisons were made with two-tailed Student’s *t* test in D, E, G, and J. ∗*p* < 0.05, ∗∗*p* < 0.01, and ∗∗∗*p* < 0.001.

To investigate the mechanisms underlying GLUT10-containing vesicle targeting to mitochondria, we examined the potential role of an evolutionarily conserved vesicle trafficking motif (YXXΦ) identified in the C-terminal region of GLUT10 (YXXΦ, Y denotes tyrosine; X is any amino acid; and Φ is an amino acid with a bulky hydrophobic side group, such as leucine, isoleucine, methionine, valine or phenylalanine) (Figure 5A). This motif is known to mediate protein rapid internalization and sorting from the PM to endosomal and lysosomal compartments.^27^ To assess its functional role in GLUT10 trafficking, we generated a deletion mutant lacking this motif (GLUT10d/GFP) (Figure 5B). Confocal microscopy revealed that GLUT10 exhibited minimal localization at the PM, whereas the deletion of the YXXΦ motif markedly enhanced GLUT10 PM localization (Figures 5C and 5D). Given that GLUT10 mediates DHA uptake, which is intracellularly reduced to AA,^14^^,^^15^ we examined the functional consequence of this altered localization. Cells expressing more PM localized GLUT10d/GFP showed significantly increased intracellular AA levels compared to WT GLUT10 expressing cells (Figure 5E), indicating that the PM localization of GLUT10 is crucial for increasing DHA uptake and intracellular AA levels. Furthermore, the increase in GLUT10d/GFP on the PM corresponded to a decrease in mitochondrial GLUT10 targeting (Figures 5F and 5G), suggesting that the YXXΦ motif is essential for GLUT10 endocytosis from the PM and its subsequent transport to mitochondria. To further validate the role of the YXXΦ motif, we replaced the C-terminal 10 amino acids of GLUT1 with the corresponding sequence from GLUT10, generating a chimeric GLUT1/YXXΦ/GFP construct (Figure 5H). While GLUT1/GFP was predominantly localized to PM, GLUT1/YXXΦ/GFP showed reduced PM localization and exhibited much broader intracellular distribution (Figures 5I and 5J). These findings reinforce that the YXXΦ motif is critical for mediating endocytic trafficking of GLUT10 from the PM toward intracellular compartments, including mitochondria.

Given that the YXXΦ motif is typically recognized by the adaptor protein-2 (AP-2) complex to facilitate clathrin-mediated endocytosis,^28^ we next tested whether GLUT10 undergoes YXXΦ-mediated PM endocytosis via a clathrin-mediated pathway. As predicted, the results showed that GLUT10/GFP colocalized with clathrin (Figures S6A and S6B). Furthermore, inhibiting clathrin-AP2-mediated endocytosis with chlorpromazine (CPZ; inhibits the assembly of clathrin and AP2 on endosomal membrane^29^) enhanced GLUT10 PM localization (Figure 5K), supporting a YXXΦ motif- and clathrin-dependent endocytic mechanism. To directly assess whether this endocytic pathway mediates GLUT10 mitochondrial targeting, we utilized a T-REx-293 inducible GLUT10/V5 expression system, which allowed temporal control of GLUT10 expression and tracking of newly synthesized protein, overcoming the detection limitations posed by pre-existing mitochondrial GLUT10 in the constitutive system. In this system, CPZ treatment effectively prevented the GLUT10 accumulation in the mitochondrial fraction (Figures 5L, 5M, and S7), while total GLUT10/V5 levels or overall mitochondrial protein markers remained unchanged (Figures 5M and 5N). These results indicate that clathrin-mediated PM endocytosis is essential for GLUT10 targeting to mitochondria.

Taken together, our findings led us to conclude that the YXXΦ motif- and clathrin-mediated PM endocytosis of GLUT10 contribute to its mitochondrial targeting. Furthermore, the PM localization of GLUT10 is crucial for enhancing DHA uptake and increasing intracellular AA levels.

### RAB5-positive endosomes facilitate GLUT10 mitochondrial trafficking

**Figure 6:**
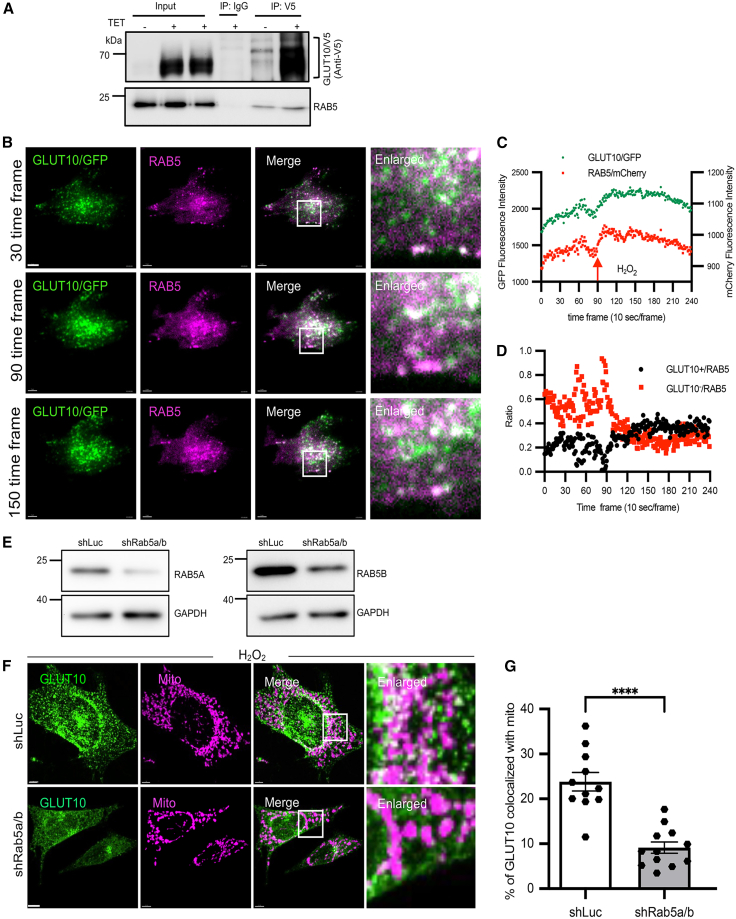
Oxidative stress-induced GLUT10 PM targeting, endocytosis, and endosomal trafficking to mitochondria (A) RAB5 is present in GLUT10-containing vesicles. Immunoblot analysis of GLUT10/V5 and RAB5 in GLUT10/V5 expressing T-REx-293 cells before (Input) and after pulling down of GLUT10-containing vesicles using anti-V5 antibodies. Blots were probed with anti-V5 and anti-RAB5 antibodies. (B–D) Oxidative stress increases GLUT10 and RAB5 localization in the plasma membrane area. (B) TIRF images of plasma membrane area of MOVAS cells co-expressing GLUT10/GFP (green) and RAB5A/mCherry (magenta), captured at time frames 30, 90, and 150 (10 s/frame). (C) Quantification of mean fluorescence intensity of GLUT10 (green) and RAB5 (red) in plasma membrane area before and after treatment with 50 μM of H_2_O_2_. (D) Ratio of GLUT10-positive RAB5 vesicles (GLUT10+/RAB5) and GLUT10-negative RAB5 vesicles (GLUT10-/RAB5) before and after H_2_O_2_ treatment. Scale bars, 15 μm. (E) Validation of RAB5A and RAB5B knockdown. Immunoblot to analyze RAB5A and RAB5B levels in control (shLuc) and RAB5A/B double knockdown (shRab5a/b) MOVAS cells. GAPDH is used as the loading control. (F) GLUT10 mitochondrial localization is impaired by RAB5A/B knockdown. Representative confocal images of GLUT10/GFP in control MOVAS cells (shLuc) and RAB5A/B double knockdown (shRab5a/b) conditions following treatment with 100 μM H_2_O_2_. *Green,* GLUT10/GFP; *magenta*, ATP5A1 IF for mitochondrial marker; *white,* merged. Scale bars, 5 μm. (G) Quantification of GLUT10 colocalized with mitochondria. Relative colocalization levels of GLUT10 with mitochondria are shown in (F). Data are presented as mean ± SEM, *n* = 11–12 cells from 3 independent experiments. Statistical comparisons were made with two-tailed Student’s *t* test, ∗∗∗∗*p* < 0.0001.

To investigate the mechanisms directing endocytosed GLUT10 toward mitochondria, we isolated GLUT10-containing vesicles and performed proteomic analyses to identify potential regulatory proteins. Among the proteins identified in GLUT10-containing vesicles from GLUT10/V5-expressing T-REx-293 cells were several vesicle trafficking regulators, including RAB family members (RABs) (Table S2). Immunofluorescence staining revealed that GLUT10/GFP exhibited substantially higher colocalization with endogenous RAB5 compared to other RABs (Figures S8A and S8B). Consistently, RAB5, a key regulator of cargo sorting and trafficking in the early endosome (EE),^30^ was detected in GLUT10/V5-containing vesicles isolated from GLUT10/V5-expressing T-REx-293 cells (Figure 6A).

To examine GLUT10 recruitment to the PM and its association with RAB5-positive EEs, we used total internal reflection fluorescence (TIRF) microscopy to track GLUT10 and RAB5 dynamics near the PM. H_2_O_2_ treatment was used to enhance GLUT10 trafficking. Upon H_2_O_2_ stimulation, GLUT10 rapidly accumulated at the PM region, accompanied by a modest rise in RAB5 signal (Figures 6B, 6C, and Video S6), indicating that oxidative stress promotes GLUT10 PM localization and recruits RAB5-positive vesicles. Quantitative analysis revealed that prior to H_2_O_2_ treatment, most RAB5 vesicles lacked detectable GLUT10 (GLUT10-/RAB5), resulting in a high GLUT10-/RAB5 ratio. Following H_2_O_2_ exposure, the proportion of RAB5 vesicles containing GLUT10 (GLUT10+/RAB5) increased, with a corresponding reduction in the GLUT10-/RAB5 ratio (Figures 6B and 6D). This trend was reproducible across multiple independent experiments despite variability in fluorescence intensity caused by transfection efficiency. Together, these data indicate that oxidative stress enhances GLUT10 accumulation at the PM and its association with RAB5-positive vesicles.

Video S6. Live time-lapse TIRF microscopy imaging illustrating GLUT10/GFP in the plasma membrane region MOVAS cells co-expressing both GLUT10/GFP and RAB5A/mCherry. An initial 15 min recording shows the control cell without H_2_O_2_ addition. Then, 50 μM H_2_O_2_ was added, and recording was continued for another 25 min. Imaging was performed at the rate of 6 frames/min (10 s/frame). Scale bars, 15 μm.

To determine the functional role of RAB5-positive vesicles in GLUT10 mitochondrial trafficking, we performed a combined knockdown of the major endocytic RAB5 isoforms, RAB5A and RAB5B (RAB5AB KD), in MOVAS cells (Figure 6E). Given their high sequence identity (>90%) and overlapping roles in endocytosis and EE sorting,^31^ RAB5AB knockdown provides an effective loss-of-function model for RAB5-dependent trafficking. Under these conditions, RAB5AB knockdown markedly reduced the abundance of GLUT10-containing vesicles, decreased GLUT10 colocalization with EEs (Figure S9), and significantly attenuated GLUT10 mitochondrial localization, even under H_2_O_2_ treatment (Figures 6F and 6G). In contrast, knockdown of RAB7, which regulates EE-to-late endosome progression and promotes cargo retention in EEs,^32^ did not reduce GLUT10 retention within EEs or its mitochondrial colocalization (Figure S10). These findings indicate that RAB5-regulated EEs, but not RAB7-regulated late endosomes, are required for efficient targeting of GLUT10 to mitochondria. Accordingly, the disruption of RAB5-dependent PM endocytosis and EE sorting prevents GLUT10 entry into this trafficking pathway, resulting in reduced its EEs and mitochondrial colocalization.

**Figure 7:**
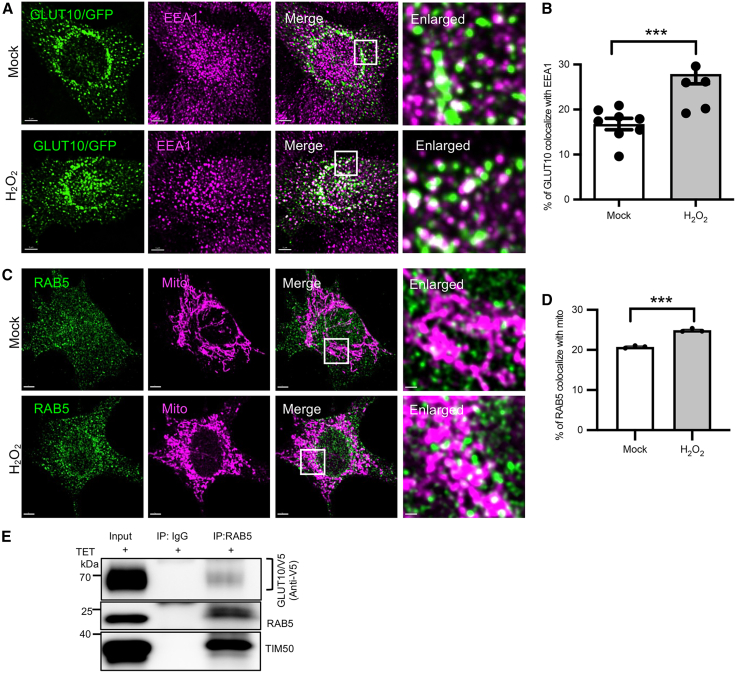
RAB5 mediates GLUT10 mitochondrial targeting (A and B) Oxidative stress increases GLUT10 colocalized with EEA1. (A) Confocal images of GLUT10/GFP colocalized with EEA1. MOVAS cells expressing GLUT10/GFP were treated with 50 μM of H_2_O_2_ for 24 h and stained by immunofluorescence for EEA1. *Green,* GFP; *magenta*, EEA1; *white,* merged. Scale bars, 5 μm. (B) Quantification of GLUT10 colocalized with EEA1, as in A. Data are shown as mean ± SEM, n = total 5–8 independent experiments with 10 cells per group in each experiment. (C and D) Oxidative stress increases RAB5 colocalized with mitochondria. (C) Confocal images of RAB5 colocalized with ATP5A1 (Mito, mitochondrial marker). MOVAS cells were treated with 50 μM of H_2_O_2_ for 24 h and stained by immunofluorescence for RAB5 and ATP5A1. *Green,* RAB5; *magenta*, ATP5A1; *white,* merged. Scale bars, 5 μm. (D) Quantification of GLUT10 colocalized with mitochondria as in F. Data are shown as mean ± SEM, *n* = 3 independent experiments with 10 cells per group in each experiment. (E) Detection of interactions among GLUT10, RAB5, and TIM50. Immunoblot analysis of GLUT10/V5, RAB5, and TIM50 in mitochondria-enriched fractions from GLUT10/V5 expression T-REx-293 cells, before (Input) or after IP using either control IgG or anti-RAB5 antibodies. Blots were probed with anti-V5, anti-RAB5, and anti-TIM50 antibodies. Statistical comparisons were made with two-tailed Student’s *t* test in B and D, ∗∗∗*p* < 0.001.

Given that oxidative stress enhances RAB5 endosome interactions with mitochondria,^33^ we next examined whether GLUT10 traffics from RAB5-positive vesicles to mitochondria under oxidative stress. Confocal imaging revealed that H_2_O_2_ treatment significantly increased colocalization of GLUT10 with EEA1 and promoted RAB5-vesicles colocalization with mitochondria (Figures 7A–7D). Live-cell imaging further revealed GLUT10-containing RAB5 vesicles approaching and associating with mitochondria (Figures S11A–S11D), and line-scan analysis confirmed the spatial overlap among RAB5, GLUT10/GFP, and mitochondrial signals (Figures S11E and S11F). Consistently, subcellular fractionation of GLUT10/V5-expressing T-REx-293 cells demonstrated a gradual increase in GLUT10/V5 abundance within RAB5- and mitochondria-enriched fractions following the induction of GLUT10/V5 expression (Figure S11G and S11H), in agreement with the colocalization of endogenous GLUT10 and RAB5 in the mitochondrial-enriched fractions (Figure S1E). Moreover, co-IP analyses detected interactions among RAB5, GLUT10/V5, and mitochondrial translocase TIM50 in mitochondria-enriched fractions (Figure 7E).

Collectively, these results demonstrate that GLUT10 traffics from the PM through RAB5-dependent endocytosis to EEs, and that proper EE sorting is critical for efficient targeting of GLUT10 to mitochondria.

### Disruption of GLUT10 trafficking and subcellular localization alters intracellular AA homeostasis

**Figure 8:**
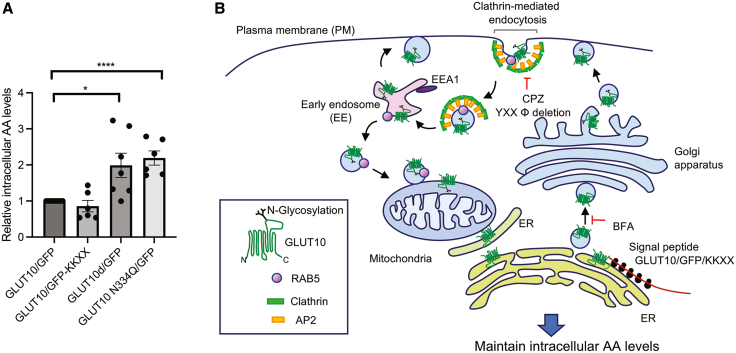
Disrupting intracellular trafficking and subcellular localization of GLUT10 affects intracellular AA levels; proposed model of GLUT10 intracellular trafficking (A) AA hemostasis was analyzed by determining the intracellular AA levels in 293T cells transfected with different versions of GLUT10 and cultured in medium containing 75 μM AA for 2 days. Data are shown as mean ± SEM, *n* = 6 independent experiments. Statistical comparisons were made with a two-tailed Student’s *t* test. ∗*p* < 0.05 and ∗∗∗∗*p* < 0.0001. (B) Model illustrates the transport of GLUT10 from the ER to mitochondria. The SP of GLUT10 targets the protein to the ER/Golgi, where it undergoes N-glycosylation. Treatment with BFA results in the retention of GLUT10 in ER-like structures. GLUT10/GFP/KKXX fusion proteins restrain GLUT10 in the ER system and reduce GLUT10 mitochondrial targeting. Deletion of the YXXΦ motif in GLUT10 or inhibition of clathrin-mediated plasma membrane (PM) endocytosis by CPZ treatment both result in GLUT10 enrichment at the PM and reduced GLUT10 mitochondrial targeting. Endocytosed GLUT10 is localized to RAB5-vesicles and targeted to mitochondria. Notably, GLUT10 in the mitochondrial fraction is N-glycosylated. Overall, we propose that GLUT10 is first targeted to the ER and Golgi for N-glycosylation, then targeted to the PM, endocytosed from PM via its YXXΦ motif- and the clathrin-mediated pathway, and finally targeted to mitochondria through an endosomal-mediated pathway.

To further explore the physiological significance of GLUT10 intracellular trafficking, we investigated how disrupting the process affects intracellular AA homeostasis in 293T cells cultured with AA. Cells expressing GLUT10d/GFP and GLUT10N334Q/GFP, which show enhanced PM localization, exhibited significantly higher intracellular AA levels compared to cells expressing wild-type GLUT10/GFP control (Figure 8A). These findings highlight that the proper intracellular trafficking of GLUT10 is crucial for regulating intracellular AA homeostasis and suggest that PM localization enhances DHA uptake, thereby elevating intracellular AA levels.

## Discussion

Protein targeting to the endomembrane system and mitochondria typically involves distinct pathways; here, we identify a unique trafficking route for GLUT10 that links these compartments. GLUT10 is first delivered from the ER-Golgi to the PM, then internalized to EEs, and finally trafficked to mitochondria via endosomal vesicles (Figure 8B). Our study reveals three key findings: (1) GLUT10 is resides at the PM, where it enhances DHA uptake and maintains intracellular AA homeostasis; (2) dynamic interactions between the endomembrane system and mitochondria facilitate the redistribution of N-glycosylated proteins across cellular compartments; and (3) oxidative stress amplifies this trafficking pathway, increasing PM localization and endocytosis, subsequently promoting mitochondrial targeting, and thereby modulates intracellular AA levels. Collectively, these findings uncover a previously unrecognized mechanism linking endomembrane-mitochondrial interactions to cellular homeostasis and stress resilience, extending GLUT10’s functional relevance beyond ATS.

The clinical phenotypes resulting from loss of GLUT10 function predominantly affect connective tissues, including major arteries and skin abnormalities observed in ATS. Given that GLUT10 is highly expressed in smooth muscle cells^14^^,^^15^ and is also present in fibroblast,^34^^,^^35^ we primarily used ASMCs (A10 and MOVAS), which have a larger cell size, to visualize subcellular localization and dynamics of intracellular trafficking, with fibroblast-like 293T cells providing complementary support. Live-cell imaging of GLUT10/GFP fusion proteins enabled visualization of trafficking dynamics, whereas inducible expression of GLUT10/V5 in T-REx-293 cells minimized potential artifacts associated with continuous overexpression. Key findings were further validated by detecting endogenous GLUT10 via subcellular fractionation and immunoblotting. Although the overall ER-Golgi-PM-mitochondria trafficking route was conserved across systems, variations in N-glycosylation levels, subcellular abundance, trafficking efficiency, and compartmental distribution were observed in different cell types and expression systems, likely reflecting intrinsic cellular characteristics as well as bottlenecks introduced by overexpression or inducible expression approaches.

The intracellular trafficking of GLUT10 emerges as a central determinant of its function. Our results revealed that GLUT10 dynamically redistributes among subcellular compartments rather than residing at a fixed location. This behavior parallels that of GLUT4, which cycles between the trans-Golgi network, endosomes, and storage vesicles and is translocated to the PM in response to insulin to enhance glucose flux.^36^ In contrast to class I transporters, class III GLUTs, including GLUT6, GLUT8, GLUT10, and GLUT12, are predominantly localized to endomembrane compartments, such as lysosomes, ER, and Golgi,^15^^,^^37^^,^^38^ however, the functional roles of these intracellular pools and the mechanisms governing their trafficking have remained poorly understood. Under stress, we observed redistribution of GLUT10 from the ER to mitochondria, consistent with our previous findings.^14^^,^^15^ Importantly, we further detected GLUT10 at the PM, within EEs, and in RAB5-positive vesicles adjacent to mitochondria, revealing a previously unrecognized communication axis between the endomembrane system and mitochondria. Stress enhanced GLUT10 targeting to the PM and EEs, thereby promoting its trafficking to mitochondria. PM localization of GLUT10 increases uptake of DHA and raises intracellular AA levels, providing direct evidence for a functional PM pool of GLUT10 and highlighting its role in maintaining AA homeostasis during stress adaptation.

Our data define an ER-Golgi-PM trafficking route that enables delivery of an N-glycosylated transmembrane protein, GLUT10, associated with mitochondria. Although mitochondrial proteins can carry N-linked glycans, their functions and trafficking routes remain poorly characterized. Proteomic studies have identified N-glycosylated proteins in mitochondrial fractions,^6^^,^^39^ and mutations in enzymes responsible for N-glycan addition or removal cause mitochondrial defects.^40^ Nevertheless, how glycoproteins processed in the ER-Golgi system reach the mitochondria has remained unclear. We demonstrate that N-glycosylated GLUT10 exits from the ER-Golgi, traffics through the PM and EEs, and is subsequently trafficked to mitochondria. Disrupting GLUT10 N-glycosylation increased its PM retention, reduced mitochondrial colocalization, and altered intracellular AA levels. Immunoblot analyses revealed two molecular forms of GLUT10: a higher molecular weight species enriched in mitochondria fractions and the lower molecular weight species enriched in ER fractions (Figure S1E), suggesting preferential mitochondrial association of full glycosylated GLUT10. Under BFA treatment (Figure 4C), indicating that a partially glycosylated pool may access mitochondria directly from the ER. (Figure 2F). While the functional significance of these distinct mitochondrial pools remains to be clarified, our data indicate that ER-Golgi N-glycosylation and PM targeting are required for efficient mitochondrial targeting of mature N-glycosylated GLUT10. These findings reveal a previously unrecognized ER-Golgi-PM pathway for mitochondrial delivery of an N-glycosylated transmembrane protein and suggest that similar mechanisms may apply to other mitochondrial transmembrane glycoproteins.

We observed that GLUT10 carries a tyrosine-based sorting motif (YXXΦ) within its C-terminal tail that may distinguish its trafficking itinerary from other intracellular GLUTs. Other GLUTs rely on distinct sorting signals to regulate their subcellular localization. For example, GLUT4 utilizes an N-terminal FQQI motif together with a C-terminal di-leucine motif for intracellular retention^41^; GLUT12 contains two di-leucine motifs that influence Golgi and PM localization^42^; and GLUT8 uses a single N-terminal di-leucine motif to target late endosomes or lysosomes.^43^ These sorting signals can mediate endocytosis and influence cargo sorting and trafficking to specific subcellular compartments.^43^^,^^44^^,^^45^ YXXΦ motifs typically mediate clathrin-dependent internalization from the PM and guide cargo toward endosomal or lysosomal pathways. Importantly, the precise positioning of a YXXΦ motif related to transmembrane domains, as well as the number of downstream residues of the motif, critically influences compartmental targeting (Table S3).^27^^,^^46^ In GLUT10, the YXXΦ motif resides in the C-terminal cytosolic tail, followed by six residues and separated by 36 residues from the predicted transmembrane domain, an arrangement distinct from previously characterized YXXΦ motifs (Table S3). This unique spatial configuration may contribute to GLUT10 endosomal sorting and highlights how subtle variations in sorting signal architecture can diversify intracellular trafficking routes.

Recent studies have highlighted the functional importance of interactions between mitochondria and endosomes/lysosomes in lipid and ion transfer, along with mitochondrial quality control.^47^ Oxidative stress enhances RAB5-dependent endosome-mitochondria interactions^33^ and precedes mitochondrial outer membrane permeabilization,^48^ implicating RAB5-positive endosomes in adaptive mitochondrial response. In this context, our findings identify RAB5-regulated EEs as a key intermediate in GLUT10 trafficking to mitochondria under oxidative stress. These results support a model in which dynamic endosome-mitochondria interactions enable rapid redistribution of a transmembrane protein in response to cellular redox changes, expanding current understanding of the functional crosstalk between these organelles.

In summary, we provide evidence supporting the existence of a protein-targeting pathway linking the endomembrane system to mitochondria. The dynamic subcellular localization of GLUT10 in the endomembrane system and mitochondria might fine-tune GLUT10-mediated transport of DHA and titrate intracellular and compartmental AA levels in response to environmental conditions. Although the detailed mechanisms of GLUT10 targeting via endosomes-mitochondria interaction require further investigation, our study uncovers a previously unknown layer of intrinsic regulation that controls the subcellular distribution of an N-glycosylated transmembrane protein functional in different subcellular compartments.

### Limitations of the study

A limitation of this study is that fluorescence colocalization lacks sufficient spatial resolution to define the precise nature of the endosome-mitochondria interaction. Accordingly, we cannot distinguish between stable endosome-mitochondria tethering or other forms of close membrane association, and the molecular mechanisms underlying these interactions remain to be defined. Future studies using super-resolution imaging and targeted mechanistic approaches will be required to address these questions.

## Resource availability

### Lead contact

Further information and requests for resources and reagents should be directed to and will be fulfilled by the lead contact, Yi-Ching Lee (yiching@gate.sinica.edu.tw).

### Materials availability

This study did not generate new materials.

### Data and code availability

• All data reported in this paper will be shared by the lead contact upon request.
• The original code/software is freely available online at https://github.com/WeiChenChu/TIRF_vesicle_colocalize_analysis, and https://github.com/WeiChenChu/Glu10_Membrane_Signal_Quantification.
• Any additional information required to reanalyze the data reported in this work paper is available from the lead contact upon request.

## Acknowledgments

We thank the Imaging, Biochemistry and Single Molecule Lab core facilities at the Institute of Cellular and Organismic Biology, Academia Sinica, for the technical support in various imaging and biochemistry instruments. We acknowledge Dr. Marcus Calkins for English editing.

Funding Acknowledgment: This study was supported by grants from 10.13039/501100001869Academia Sinica, Taiwan (AS 105-TP-B04), the Ministry of Science and Technology (MOST), Taiwan (10.13039/501100004663MOST 108-2320-B-001-022), and National Science and Technology Council (NSTC), Taiwan (NSTC 113-2320-B-001-008) to Y.C.L. The funders had no role in study design, data collection, and analysis, the decision to publish, or manuscript preparation.

## Author contributions

Conceptualization, Y.C.L. and A.C.J.; methodology, Y.C.L., A.C.J., YWS., Y.F.J., P.Y.L, C.Y.F., S.C.H., W.C. H., and W. C. C.; investigation, A.C.J., Y.W.S., H.W.L., M.Y.T., Y.F.J., S.C.H., P.Y.L, C.Y.F. W.C. H., and W. C. C.; writing – original draft, Y.C.L. and A.C.J.; all authors review and editing the manuscript; funding acquisition, Y.C.L.; supervision, Y.C.L.

## Declaration of interests

The authors declare no competing interests.

## STAR★Methods

### Key resources table

**Table:** 

REAGENT or RESOURCE	SOURCE	IDENTIFIER
**Antibodies**		

V5	Thermo Fisher Scientific	Cat# R960-25, RRID: AB_2556564
GFP	Abcam	Cat# ab6556, RRID: AB_305564
GLUT10	Abcam	Cat# ab110528, RRID:AB_3099701
GLUT10	Santa Cruz Biotechnology	Cat# sc-398495, RRID:AB_2940967
TIM50	Proteintech	Cat# 22229-1-AP, RRID:AB_2879039
TOM20	Santa Cruz Biotechnology	Cat# sc-11415, RRID:AB_2207533
ATP5A1	Thermo Fisher Scientific	Cat# 459240, RRID:AB_2532234
Calreticulin	Merck Millipore	Cat# 06–661, RRID:AB_11214165
Calnexin	Abcam	Cat# ab 22595 RRID:AB_2069006
KDEL	Enzo Life Sciences	Cat# ADI-SPA-827, RRID:AB_10618036
GM130	Abcam	Cat# ab52649 RRID:AB_880266
beta Actin	Gene Tex	Cat# GTX110564, RRID:AB_10618080
GAPDH	Proteintech	Cat# 10494-1-AP, RRID:AB_2263076
RAB5	Santa Cruz	Cat# sc, 46692, RRID:AB_628191
RAB5A	Proteintech	Cat# 11947-1-AP, RRID:AB_2269388
RAB5B	Santa Cruz Biotechnology	Cat# sc-373725, RRID:AB_10917429
RAB7	Proteintech	Cat# 55469-1-AP RRID:AB_11182831
RAB11	Abcam	Cat# Ab3612 RRID:AB_10861613
EEA1	Abcam	Cat# ab109110, RRID:AB_10863524
Goat anti-mouse IgG (HRP)	Sigma-Aldrich	Cat# A0168, RRID:AB_257867
Goat anti-rabbit IgG (HRP)	Sigma-Aldrich	Cat# A0545, RRID:AB_257896
Goat anti-mouse IgG (AF568)	Thermo Fisher Scientific	Cat# A-11004, RRID:AB_2534072
Goat anti-rabbit IgG (AF 647)	Thermo Fisher Scientific	Cat# A-21245, RRID:AB_2535813

**Bacterial and virus strains**		

DH5a competent cells	YB Biotech	Cat# FYE678

**Chemicals, peptides, and recombinant proteins**		

Mitotracker Red	Thermo Fisher Scientific	Cat# M7512
ER tracker Red	Thermo Fisher Scientific	Cat# E34250
BODIPY™ TR Ceramide (Golgi tracker)	Thermo Fisher Scientific	Cat# D7540
Brefeldin A	Sigma-Aldrich	Cat# B7651
Chlorpromazine	Sigma-Aldrich	Cat# C-8138
Tetracycline	Sigma-Aldrich	Cat# T7660
Hygromycin B	Sigma-Aldrich	Cat# 10843555001
Proteinase K	Thermo Fisher Scientific	Cat# 4333793
Digitonin	Sigma-Aldrich	Cat# D141
Puromycin	Thermo Fisher Scientific	Cat# A1113803
Protein G mag Sepharose Xtra	Cytiva	Cat# 28967070
Lipofectamine 3000	Thermo Fisher Scientific	Cat# L3000001
Percoll	Sigma-Aldrich	Cat# P1644

**Critical commercial assays**		

PNGase F glycan cleavage kit	Thermo Fisher Scientific	Cat# A39245
Mitochondria Isolation Kit for Cultured Cells	Thermo Fisher Scientific	Cat# 89874
Flp-In™ T-REx™ Core Kit	Thermo Fisher Scientific	Cat# K650001
QuikChange Site-Directed Mutagenesis Kit	Stratagene	Cat# 200518
ECL detection kit	Cytiva	Cat# RPN2232
Ascorbic acid assay kit	Abcam	Cat# ab65656

**Deposited data**		

Code for vesicle colocalization	This paper	https://github.com/WeiChenChu/TIRF_vesicle_colocalize_analysis
Code for membrane signal quantification	This paper	https://github.com/WeiChenChu/Glu10_Membrane_Signal_Quantification

**Experimental models: Cell lines**		

Mouse aortic smooth muscle (MOVAS) cells	ATCC	Cat# CRL-2797
Rat aortic smooth muscle cells (A 10)	ATCC	Cat# CRL-1476
HEK293T cells	ATCC	Cat# CRL-11268
Human aortic smooth muscle (hASMC) cells	Cell Applications	Cat# 354-05A

**Oligonucleotides**		

Refer to supplementary information	Table S4	N/A

**Recombinant DNA**		

pEGFP-N1	Clontech	Cat# 6085-1
pcDNA3.1- MitoDsRed	gift from Dr. Ming-Der Pern (College of Life Sciences and Medicine, National Tsing Hua University, Taiwan)	N/A
Rab5a-pmCherryC1	Addgene	Cat# 27679
Rat Rab5- pECFP-C1	This paper	N/A
pEGFP-N1-GLUT10-GFP (GLUT10/GFP)	This paper	N/A
pEGFP-N1-GLUT10-GFP-KKXX (GLUT10/GFP-KKXX)	This paper	N/A
pEGFP-N1-GLUT10 SP-GFP-KDEL (SP/GFP-KDEL)	This paper	N/A
pEGFP-N1-GLUT10d-GFP (GLUT10d/GFP)	This paper	N/A
pEGFP-N1-GLUT1 YXXΦ –GFP (GLUT1 YXXΦ/GFP)	This paper	N/A
pEGFP-N1-GLUT1-GFP (GLUT1/GFP)	This paper	N/A

**Software and algorithms**		

ImageJ	NIH	RRID:SCR_003070
Imaris	Oxford Instruments	RRID:SCR_007370
GraphPad Prism V10	Dotmatics	RRID:SCR_002798
ZEN black	Zeiss	RRID:SCR_018163
Huygens	Scientific Volume Imaging	RRID:SCR_014237
Xcellence	Olympus	N/A
VisionWorksLS	UVP	Version 6.8

### Experimental model and study participant details

#### Cell lines

Mouse aortic smooth muscle (MOVAS) cells (CRL-2797), rat aortic smooth muscle (A10) cells (CRL-1476), and HEK293T cells (CRL-11268) were obtained from ATCC (American Type Culture Collection, ATCC, Manassas, VA, USA) and maintained in Dulbecco’s Modified Eagle Medium (DMEM, Gibco) containing 10% fetal bovine serum (FBS) (Gibco) and 1% penicillin and streptomycin (Gibco). Human aortic smooth muscle cells (hASMC, 354-05A, Cell Applications) were maintained in SMC growth medium (311–500, Cell Applications). All cell lines were authenticated by suppliers and were confirmed to be negative for mycoplasma contamination. Cells were cultured at 37°C in a humid atmosphere with 5% CO_2_.

### Method details

#### Plasmid construction

The KDEL-tagged EGFP expression vector (GFP/KDEL) was generated by amplifying full-length EGFP with a reverse primer containing a KDEL-encoding sequence. The EGFP of the pEGFP-N1 expression vector (Clontech, Mountain View, CA, USA) was then replaced with EGFP-KDEL. The GLUT10/GFP and GLUT10/GFP/KKXX expression vectors were generated by insertion of the full-length mouse *Slc2a10* cDNA (NM_130451.1) into the pEGFP-N1 expression vector (GLUT10/GFP) or GFP/KKXX (GLUT10/GFP/KKXX) expression vector. The SP/GFP/KDEL expression construct was generated by PCR amplification of the region encoding the first 40 amino acids of GLUT10 (containing the SP) and inserted into GFP/KDEL expression vector.

The GLUT10d/GFP construct was constructed by deleting the last 10 amino acids comprising the YXXΦ motif of GLUT10 (GLUT10d/GFP) by oligonucleotide-directed mutagenesis (QuikChange Site-Directed Mutagenesis Kit, Stratagene, Santa Clara, CA, USA). The modified gene was then inserted to pEGFP-N1 expression vector. The GLUT1/GFP construct was constructed by inserting full-length mouse *Slc2a1* cDNA (NM_011400.3) into the pEGFP-N1 expression vector. The GLUT1/YXXΦ/GFP construct was created by replacing the last 10 amino acid of C-terminus of GLUT1 with the last 10 amino acids of the GLUT10 YXXΦ using two-step PCR. In Step one, the DNA fragment of *Slc2a1* from 892 bp to 915bp and the DNA fragment of *Slc2a10* (NM_130451.1) from 1548 bp to 1697 bp, which have overlapping sequences in the homologous region, were amplified. The two PCR products from Step one were then used as templates to generate a chimeric DNA fragment containing the 892 bp to 915 bp fragment of *Slc2a1* and 1548 bp −1679 bp of *Slc2a10* using the 5′ primer for *Slc2a1* fragment and the 3′ primer for *Slc2a10* amplification. GLUT1 cDNA of the GLUT1/GFP construct was replaced by the chimeric DNA fragment to generate the GLUT1/YXXΦ/GFP construct.

The Mito/dsRed construct in the pcDNA3.1 backbone, containing the mitochondrial targeting sequence from *COX8A* fused to dsRed, was a kind gift from Dr. Ming-Der Pern (College of Life Sciences and Medicine, National Tsing Hua University). The Rab5a-pmCherryC1 plasmid (Addgene plasmid #27679) was obtained from Addgene. The RAB5/CFP expression vector was generated by inserting full-length rat *Rab5* cDNA (AF027935.1) into the pECFP-C1 expression vector (Clontech, USA). The primer pairs used for construct generation are shown in the Supplementary materials (Table S4).

#### Generation of stable cell lines

T-REx-293 cells expressing GLUT10/V5 were generated using mouse *Slc2a10* (NM_130451) cDNA and Flp-In T-REx System (K650001, Invitrogen). A stable clone was selected with 200 μg/mL of Hygromycin B. GLUT10/V5 expression was induced by 5 μg/mL tetracycline. Cell transfections were carried out using Lipofectamine 3000 reagent (L3000001, Invitrogen). Stably transfected *shRab5a* and *shRab5b*, *shRab7* or *shLuc* MOVAS cells were generated by lentivirus transduction and were selected in the culture medium in the presence of 2 μg/mL puromycin (Invitrogen). shRNAs targeting mouse *Rab5a* (5′-CGCTTTGTGAAAGGCCAATTT-3′), *Rab5b* (5′-GCACTTTAATTGATGGTAGTT-3′), *Rab7* (5′-TGCTGTGTTCTGGTGTTTGAT-3′), and *Luc* control (5′-ATCACAGAATCGTCGTATGCA-3′) in PLKO vectors were purchased from the National RNAi Core Facility at Academia Sinica, Taipei, Taiwan.

#### Immunofluorescence staining

Cells were grown on coverslips and fixed in 4% paraformaldehyde. The cells were then washed and stained with primary antibodies and fluorophore-labeled secondary antibodies. Coverslips were mounted onto glass slides using VECTASHIELD anti-fade mounting medium (H-1000-10, Vector labs).

#### Confocal microscopy

Confocal images were acquired on a Laser Scanning Microscope, LSM 880 (ZEISS, Germany) using Plan-Apochromat 63x/1.4 oil immersion objective with ZEN 2.3 SP1 (black edition) software. Green and red fluorophores were excited using the 488 nm argon laser source and 561 nm diode pump solid-state laser, respectively. For live-cell imaging, the cells were grown in 35-mm glass-bottom dishes and placed in the live-cell incubation chamber. The chamber was maintained at 37°C and 5% CO_2_ to maintain normal growth conditions during the imaging process.

#### Total internal reflection fluorescent (TIRF) microscopy

TIRF images were captured using the TIRFM system built on an inverted microscope (Olympus IX81) equipped with a high-sensitivity EMCCD camera (iXon3 897, Andor Technology) and a UPONAPO 60× OTIRF objective lens (NA: 1.49; Olympus) coupled with Xcellence software (Olympus). 488 and 532 nm solid lasers were used to excite GFP and mCherry fluorophores, respectively. TIRF penetration depth was set at 100 nm. To capture real-time trafficking of GLUT10/GFP and RAB5A/mCherry, live cell images were recorded at a rate of 1 frame/10 s (6 frames/min). Recordings were made for 10 min before adding H_2_O_2_ and then another 10 min after adding H_2_O_2_.

#### Image analysis

The acquired images were processed with Huygens software (Scientific Volume Imaging) for deconvolution. Co-localization analysis was performed using Imaris 9.9.1 (Oxford instruments). In brief, intensity-based analysis was used to calculate the percentage of colocalization between two channels. The intensity threshold for each channel was adjusted manually to best represent the fluorescence signals. Colocalized was quantified as a percentage of volume A that overlaps with volume B. For example, mitochondrial colocalization of GLUT10 was determined by calculating the percentage of GLUT10/GFP-positive pixels (green) that overlap with mito-DsRed positive pixels (magenta), relative to the total pixel volume of GLUT10/GFP-positive signals. Dynamics of GLUT10/GFP vesicles in cells were characterized by tracking the positions of individual vesicles at different time points using Imaris software.

The fluorescence intensity of the whole cell or the plasma membrane was quantified using Fiji^49^ with a custom ImageJ macro. Briefly, a z-projection using the “Sum Slices” method was applied to the GFP channel images. A duplicate of the projection image was created and processed to generate a binary mask using Gaussian blur and Huang thresholding.^50^ The mask was further refined by applying the “fill-hole” function and performing size filtering using the “analyze particles” function, which resulted in a whole cell mask. The cytosol mask was then derived by iteratively applying the “Erode” operation to the whole cell mask for 10 cycles. The cell membrane mask was subsequently created by subtracting the cytosol mask from the whole cell mask. Selections for measurements were established for specific regions within the resultant masks and saved as Regions of Interest (ROIs) in FIJI. GFP intensity measurements were conducted on the original projection image, based on the ROI list. (Source code: https://github.com/WeiChenChu/Glu10_Membrane_Signal_Quantification).

To measure the fluorescence intensity of the plasma membrane area of GLUT10/GFP and RAB5/mCherry in time-series TIRF images, we used TrackMate^51^ in conjunction with the Cellpose Cyto3 model^52^ and a custom python code. Briefly, the RAB5 channel image was pre-processed with a median filter (radius = 4) to reduce noise. The filtered image was then processed using TrackMate with the Cellpose Cyto3 model to segment the plasma membrane region. The resulting label image was converted into an ROI list corresponding to each time frame, using the “Labels to 2D ROI Manager” function in the BioVoxxel 3D Box plugin (https://biovoxxel.github.io/bv3dbox/). Signal intensity within each ROI at each time point was measured using the FIJI ROI Manager.

To determine the colocalization of GLUT10/GFP and RAB5A/mCherry, time-series TIRF images were analyzed to assess vesicle colocalization over time. Initially, a cell mask was generated from the RAB5A/mCherry images using TrackMate-Cellpose (Cyto3 model) after applying a median filter with a radius of 4 pixels; this mask defined ROIs and was exported as a time-series mask image. In cases where the Cellpose analysis was not working well but the cell maintained a consistent shape across different time points, a manually defined mask image was used. The GLUT10/GFP, RAB5A/mCherry, and cell mask images were processed using custom Python code (available from the GitHub repository: https://github.com/WeiChenChu/TIRF_vesicle_colocalize_analysis) for colocalization analysis. Briefly, for each time point, GLUT10-GFP images were processed with a Difference of Gaussian (DoG) filter to reduce noise and background while enhancing vesicular structures. The DoG-filtered GLUT10 images were multiplied by the cell mask to isolate intracellular regions and subsequently segmented using Voronoi Otsu Labeling. RAB5A/mCherry images underwent similar DoG filtering and cell mask application, followed by thresholding using the triangle method to generate binary RAB5 masks. Colocalization was assessed by calculating the mean intensity overlap between GLUT10 labels and Rab5 masks (as overlap score, “1” means the GLUT10 label is full overlap with the Rab5 mask); vesicles with an overlap score between 0.5 and 1.0 were classified as colocalized. Those between 0.01 and 0.5 were classified as partially colocalized, and those with zero overlap were classified as non-colocalized. The numbers of vesicles in each category were quantified across time points, and a colocalization ratio (colocalized vesicles divided by total GLUT10 vesicles) was calculated. Data were exported to Excel files for further analysis.

#### Immunogold and APEX2 staining electron microscopy (EM)

Immunogold labeling of permeabilized whole-mount cells for EM was performed following established procedures.^53^ Briefly, cells were grown on sapphire discs (16770158, Leica) and cryo-fixed using a high-pressure freezer (HPM100, Leica). Then, the cells were subjected to freeze substitution in an AFS2 system (AFS2, Leica) with subsequent rehydration and post-fixing. Immunogold detection was performed using anti-GFP (ab6556, Abcam), followed by 1.4 nm NANOGOLD-gold cluster labeling (#2004, Nanoprobes) and silver amplification (#2012, Nanoprobes).

The APEX2 labeling and EM sample preparation were performed as previously reported.^54^ MOVAS cells were transfected with GLUT10/APEX2 vector or APEX2 vector control. Cells were fixed and then overlaid with a solution of DAB and H_2_O_2_. APEX2 catalyzed the oxidation of DAB to generate a locally deposited DAB polymer. After labeling, cells were stained with Osmium tetroxide (OsO_4_) and Uranyl acetate (UA) to improve sample preservation and contrast. Following resin-embedding, cells were trimmed and cut into 80-nm sections using an ultramicrotome (Leica EM UC7).

Electron micrographs were acquired using FEI Tecnai G2 F20 S-TWIN Transmission electron microscope (Thermo Fisher Scientific) operating at 120 kV and equipped with a 2 K × 2 K CCD camera (US1000, Gatan) at ICOB Biological Electron Microscopy Core Facility, Academia Sinica. Brightness and contrast adjustments were applied uniformly to the entire image, and no region-specific or selective modifications were performed. The regions shown in the figure panels represent enlarged views selected from the original images. Original uncropped images are provided in the supplementary materials.

#### Subcellular fractionation

Subcellular fractionation was performed following a published protocol^55^ with minor modifications. The flow-chart for subcellular fractionations are presented in Supplementary information (Figure S1C). In detail, approximately 1 × 10^8^ cells were harvested and suspended in 2 mL of sucrose homogenization medium. Cells were lysed by homogenization using a pre-cooled Dounce homogenizer. Homogenate was centrifuged at 600 ×*g* for 10 min, and the centrifugation was repeated until no pellet was visible. The supernatant was then further centrifuged three times at 10,000 ×*g* at 4°C for 10 min to obtain a pellet. The pellet was further separated on 30% Percoll by centrifugation at 95,000 ×*g* to obtain F1 and F2. The supernatant from the previous step was further concentrated at 200,000 ×*g* for 60 min at 4°C to obtain ER enriched fraction (F3). The fractions were resuspended in SDS sample buffer, and organelle enrichment was analyzed by immunoblotting with specific organelle markers.

#### Isolation of mitochondria-enriched fractions

Mitochondria-enriched fractions were isolated using the mitochondria isolation kit for cultured cells (89874, Thermo Scientific). In brief, approximately 2 × 10^7^cells were homogenized using the reagents provided and centrifuged at 700 ×*g* at 4°C for 10 min. The supernatant was further centrifuged at 12,000 ×*g*, 4°C for 15 min to obtain the mitochondria-enriched fraction. Evidence for the enrichment of the mitochondrial fractions is shown in Figure S7.

#### Immunoprecipitation and protein identification

GLUT10-interacting proteins were co-immunoprecipitated using V5 antibody (R960-25, Invitrogen) in GLUT10/V5 T-REx-293 cells expressing GLUT10/V5 after induction with 5 μg/mL tetracycline for 24 h. Cells were lysed in lysis buffer (300 mM NaCl, 50 mM Tris pH 7.5, 5% glycerol, 1% NP-40, and 1× protease inhibitor). The lysates were pre-cleaned with Protein G Mag Sepharose Xtra (Cytiva), and IP was performed with Protein G Mag Sepharose-conjugated V5 antibody. For the IP of GLUT10-containing vesicles, GLUT10/V5 expressing T-REx-293 cells were subjected to subcellular fractionation to collect the vesicle-enriched fraction. After pre-cleaning with Protein G Mag Sepharose Xtra (Cytiva), the GLUT10-containing vesicles were pulled down with Protein G Mag Sepharose-conjugated V5 antibody. The GLUT10-interacting proteins and proteins from the GLUT10-containing vesicles were further subjected to MS analysis by Orbitrap Fusion Lumos in the Proteomics Mass Spectrometry Common Facility, Institute of Biological Chemistry, Academia Sinica. Also, mitochondria-enriched fractions from GLUT10/V5 expressing T-REx-293 cells were subjected to IP using RAB5 antibody to pull down RAB5-associated mitochondria and analyzed for V5 and TIM50 by immunoblotting.

#### Proteinase K and digitonin treatment

Proteinase K digestion was performed according to the published protocol with minor modifications.^19^ Mitochondria-enriched fractions isolated from cells were resuspended in mitochondria suspension buffer (10 mM Tris-base pH 7.4, 320 mM sucrose, 1 mM EDTA). Twenty milligrams of mitochondria in 200 μL of TD buffer (49.99 mM Tris base, 274.13 mM NaCl, 20.12 mM KCl, 13.95 mM Na_2_HPO_4_) were incubated with 1 μg/mL of proteinase K (Thermo Scientific) for 1 h at room temperature (RT) (22°C–27°C). The digestion was stopped by adding 2 μL of 100 mM PMSF. For digitonin treatment, mitochondria fractions isolated from cells were resuspended in 200 μL of digitonin solution (7 mg/mL) and incubated at RT with constant mixing for 1 h. Proteins were separated by SDS-PAGE and subjected to western blot analysis.

#### Western blots

Total protein lysates or organelle fractions were separated on SDS-PAGE and transferred onto a polyvinylidene difluoride (PVDF) membrane (10600023, Amersham, Cytiva). The blots were probed with primary and secondary antibodies as listed in the antibodies section. Protein signals were detected using an enhanced chemiluminescence (ECL) kit (RPN2232, Amersham, Cytiva) and imaged with the BioSpectrum 600 detection system (UVP). Image acquisition and processing were performed using the VisionWorksLS software (UVP, Version 6.8). Chemiluminescent molecular weight markers and the protein bands were captured simultaneously. Images of pre-stained molecular weight markers were acquired separately from the same membrane and aligned for presentation without altering band positions. No splicing or selectively modifications were performed. When necessary, brightness and contrast adjustments were applied uniformly across the entire image.

#### Measurement of ascorbic acid (AA) accumulation

For DHA uptake, intracellular AA levels were measured by high-performance liquid chromatography (HPLC) as described previously.^14^ Cells were incubated in low glucose DMEM (5 mM) without FBS and containing 5 mM DHA at 37°C for 30 min. Total cell lysates were used for AA measurement. For intracellular AA homeostasis assay, AA levels were determined with an Ascorbic acid assay kit (ab65656, Abcam) according to the manufacturer’s instructions. Cells were grown in culture medium containing 75 μM AA for 48 h; AA levels were measured from whole cell lysates.

### Quantification and statistical analysis

Statistical significance between groups was analyzed by two-tailed unpaired Student’s *t* test in GraphPad Prism 10 (GraphPad Software, La Jolla, CA, USA). Data are presented as mean ± SEM. A *p*-value less than 0.05 was considered statistically significant. Statistical significance was defined as ∗*p* < 0.05, ∗∗*p* < 0.01, and ∗∗∗*p* < 0.001.

## Supplemental information

Document S1. Figures S1–S12 and Tables S1–S4

Document S2. 20260325 WB and EM UNCROPPED IMAGES with replicates
