# Supplementary material for: Plasma membrane mediated GLUT10 mitochondrial targeting regulates intracellular ascorbic acid homeostasis
Source: iScience. 2026 Apr 25;29(6):115891. doi: 10.1016/j.isci.2026.115891 (PMC13196150; doi:10.1016/j.isci.2026.115891)
Supplement: Document S1. Figures S1–S12 and Tables S1–S4 [file mmc1.pdf]

iScience, Volume *29*

Supplemental information

Plasma membrane mediated GLUT10 mitochondrial targeting regulates intracellular ascorbic acid homeostasis

Anu Chirackal Jose, Yu-Wei Syu, Hao-Wen Lai, Ming-Yuan Tsai, Yi-Fan Jiang, Shao-Chun Hsu, Po-Yen Lin, Wan-Chen Huang, Wei-Chen Chu, Chi-Yu Fu, and Yi-Ching Lee

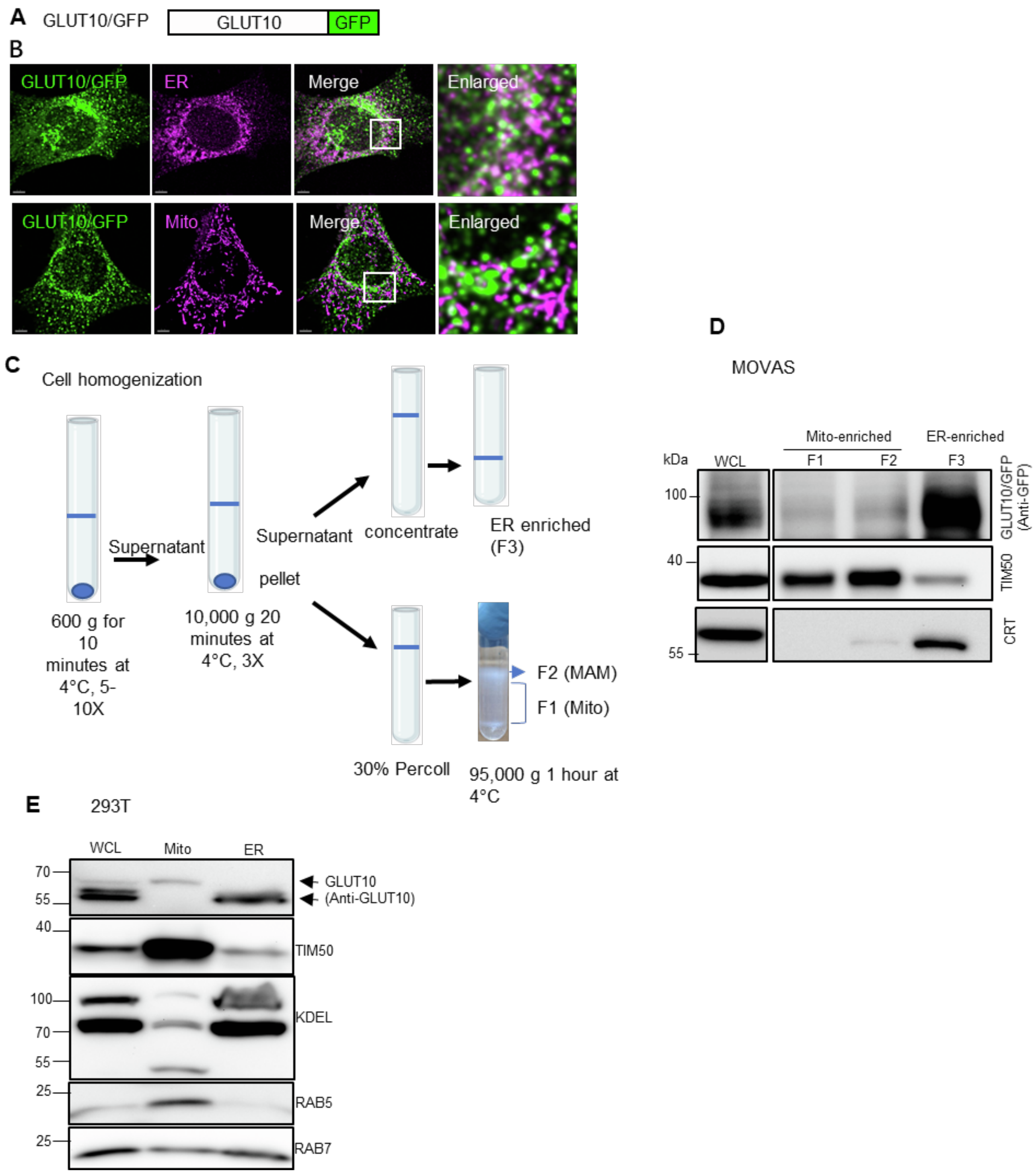

Figure S1. Analysis of GLUT10 subcellular distribution.

(**A**) Schematic of GLUT10/GFP fusion protein. (**B**) Confocal images showing colocalization of GLUT10/GFP with subcellular compartment markers in MOVAS cells. Immunofluorescence (IF) staining was performed using calreticulin (ER marker) and ATP5A1 (Mitochondrial marker). *Green,* GLUT10/GFP; *magenta*, IF signals; *white,* merged. Scale bars, 5 μm. **(C)** Flowchart illustrating subcellular fractionations. (**D**) Immunoblot analysis of GLUT10/GFP levels in subcellular fractions from GLUT10/GFP-expressing MOVAS cells. Immunoblots were probed with anti-GFP for GLUT10/GFP; anti-TIM50 (mitochondrial marker); anti-calreticulin (CRT) (ER marker). (**E**)

Immunoblots of endogenous GLUT10 in different subcellular fractions from 293T cells, including whole cell lysate (WCL), mitochondrial-enriched fractions (mito), and ER-enriched fractions. Blots were probed with anti-GLUT10; anti-KDEL (ER marker); anti-TIM50 (mitochondrial marker); anti-RAB5; and anti-RAB7.

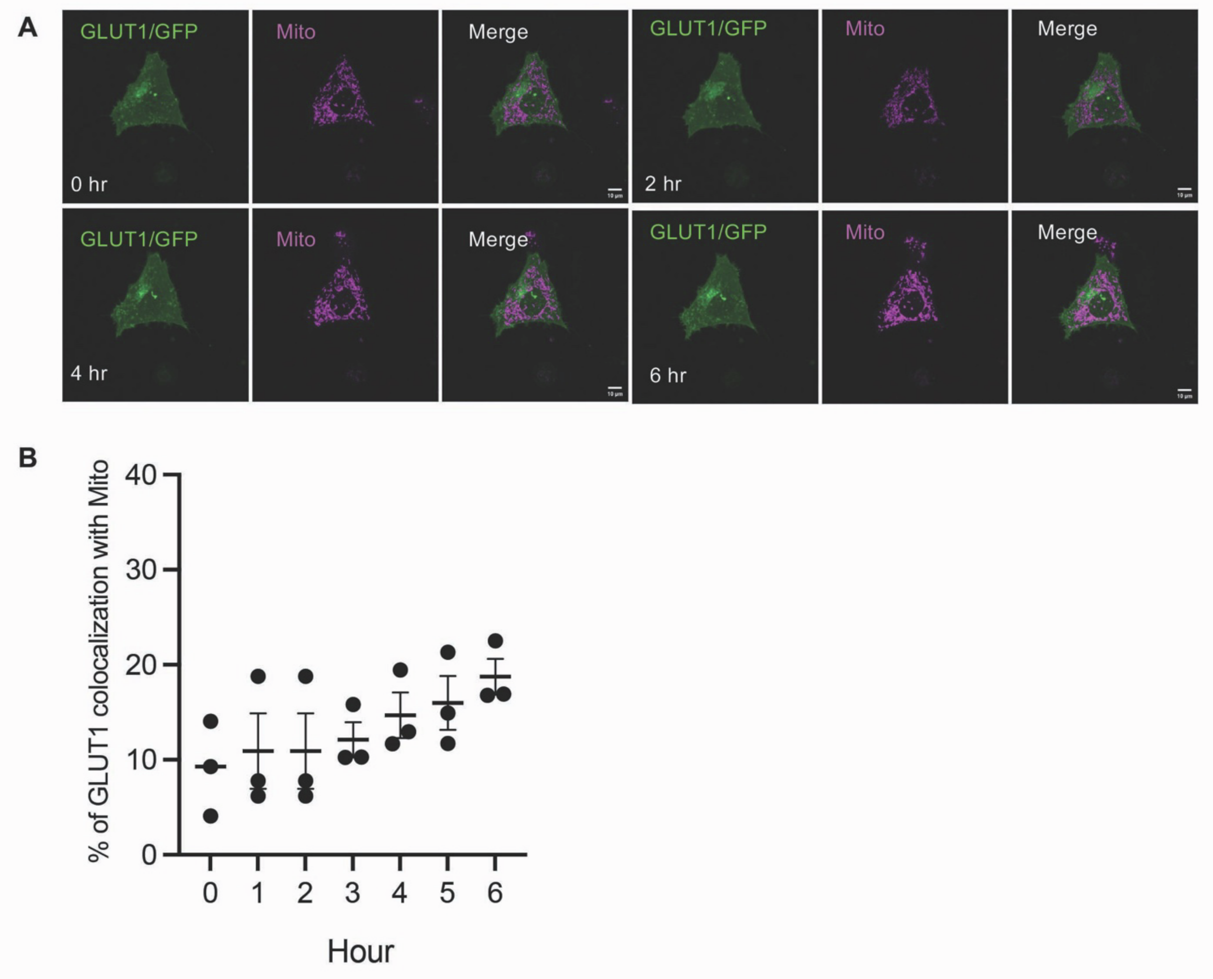

Figure S2. H_2O_2 induced oxidative stress did not affect GLUT1 mitochondria localization.

(**A**) Confocal images show colocalization of GLUT1/GFP and mitochondria in live cells. A10 cells expressing both GLUT1/GFP and Mito/DsRed were treated with 100 µM H_2O_2; imaging was performed every 1 h for 6 h. Scale bar, 10 µm. (**B**) Quantification of mitochondrial colocalization. Percentage of GLUT1/GFP signals colocalized with Mito/DsRed calculated from three cells across three independent experiments. Data represent the mean ± SEM.

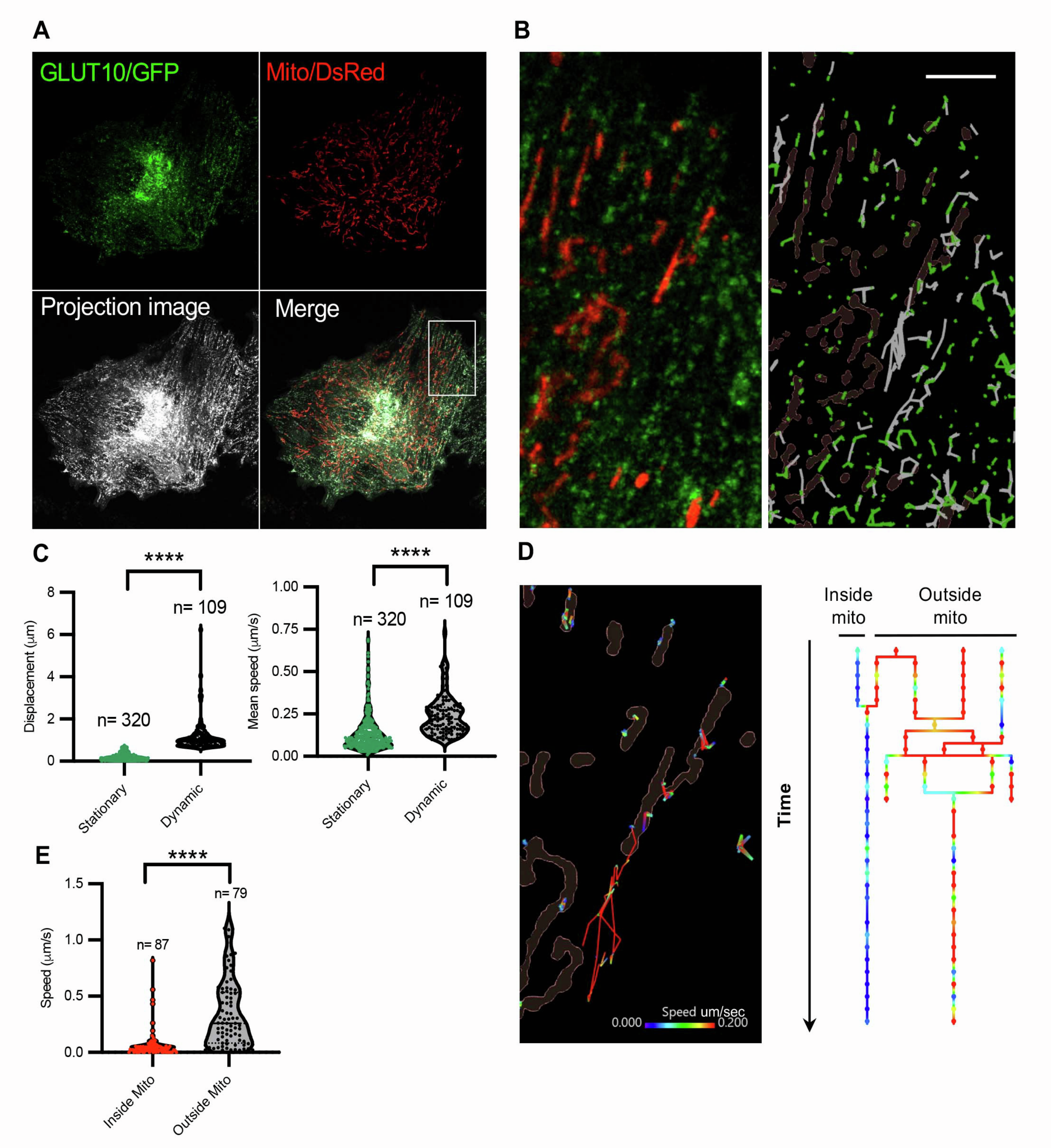

Figure S3. Kinetics of interaction between GLUT10-containing vesicles and mitochondria in response to H_2O_2 stimulation.

(**A**) Time series of confocal images shows GLUT10-containing vesicle trafficking in live A10 cells expressing both GLUT10/GFP and Mito/DsRed; cells were treated with 100 μM H_2O_2 and imaged at 1-s intervals for 30 s (Upper panels). The projection image was created from 10 sequential frames and shows the trafficking trajectories of GLUT10 vesicles (Lower panels). (**B**) Stationary (green) and dynamic (gray) GLUT10-vesicle trajectories were identified in the rectangle shown in **A**. A total of 429 trajectories of GLUT10 vesicles were analyzed. Scale bar: 5 μm. (**C**) The track displacement

lengths and mean speeds over 30 s for stationary (green) and dynamic (white) trajectories of GLUT10-containing vesicles, as in **B**. Track displacement less than 0.67 μm over 30 s was defined as stationary. Track displacement longer than 0.67 μm over 30 s was defined as dynamic. (**D**) Representative trajectories of GLUT10-containing vesicles show the reduction of instantaneous speed after entering mitochondria. The speed of GLUT10-containing vesicle trafficking was color coded. (**E**) The mean speeds of GLUT10-containing vesicle trafficking inside and outside mitochondria. A total of 166 GLUT10-containing vesicle trajectories were analyzed, as in **A**. In **C** and **E**, data represent the mean ± SEM, n = trajectories analyzed in each group, as indicated in the figure. Statistical comparisons were made by two-tailed Student's t-test, ****$P < 0.0001$.

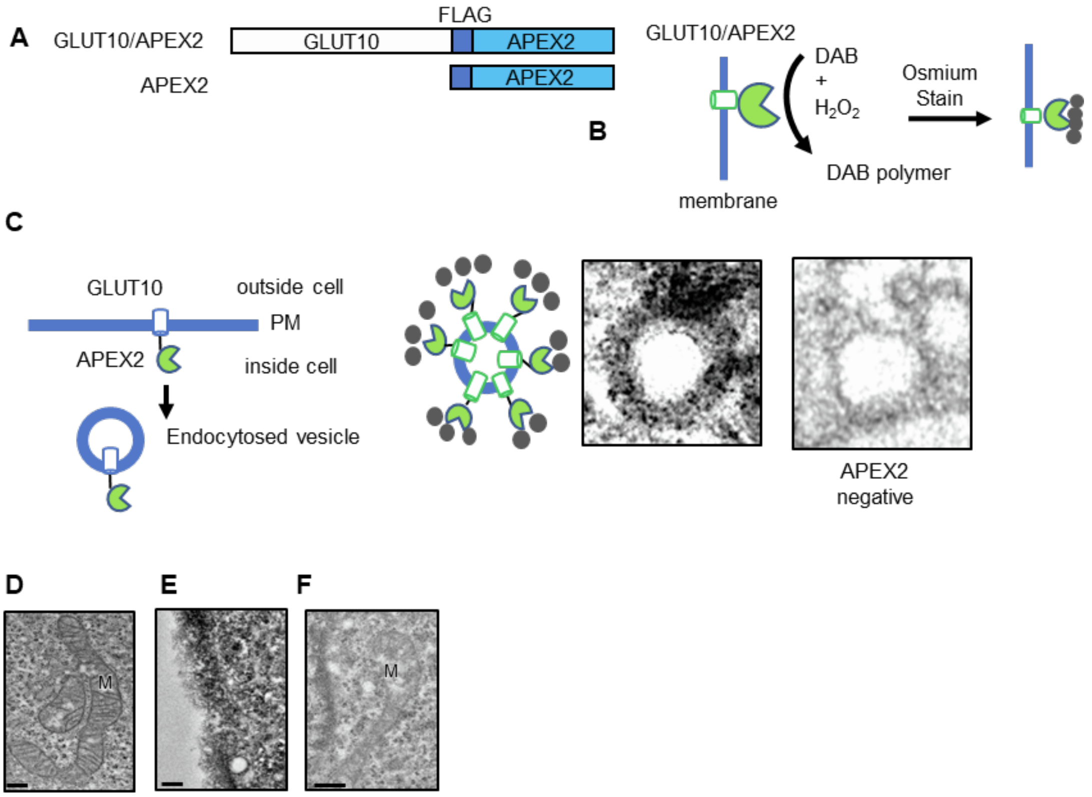

Figure S4. Electron microscopy (EM) characterization of GLUT10/APEX2 subcellular localization.

(**A**) Schematic of constructs expressing GLUT10/APEX2 and APEX2 control. (**B**) APEX2 catalyzes diaminobenzidine (DAB) polymerization in the presence of H_2O_2. The DAB polymer reacts with osmium to provide contrast for EM, indicating the localization of GLUT10/APEX2. (**C**) The schematic diagram depicts endocytosed GLUT10/APEX2-positive vesicles of enlarged EM images of GLUT10/APEX2-positive and negative endocytosed vesicles from Fig. 2J. EM image of MOVAS cells with (**D**) standard osmium fixation showing mitochondrial morphology, (**E**) APEX2 only control stained with DAB, and (**F**) negative control, without DAB staining. Scale bars, 100 nm.

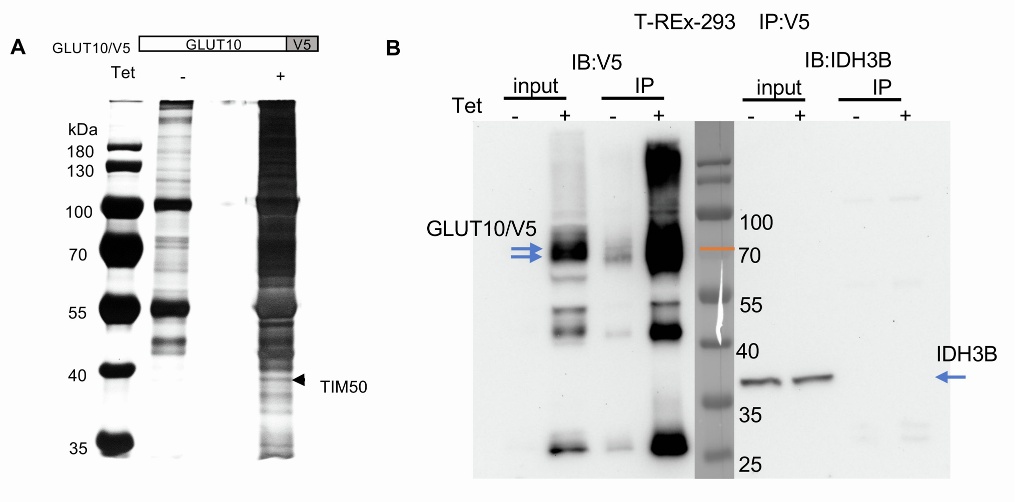

Figure S5. Isolation of GLUT10-interacting proteins.

(**A**) SDS-PAGE analysis of GLUT10/V5-interacting proteins pulled down with V5 from T-REx-293 cells with or without induction of GLUT10/V5 expression. Proteins were stained with Coomassie Blue. Arrow indicates TIM50, which was identified by MS analysis. (**B**) GLUT10 /V5 does not directly interact with IDH3B. Immunoblots detecting GLUT10/V5 and IDH3B protein levels in cell lysates from GLUT10/V5 expressing T-REx-293 cells before IP (Input) and after IP with V5.

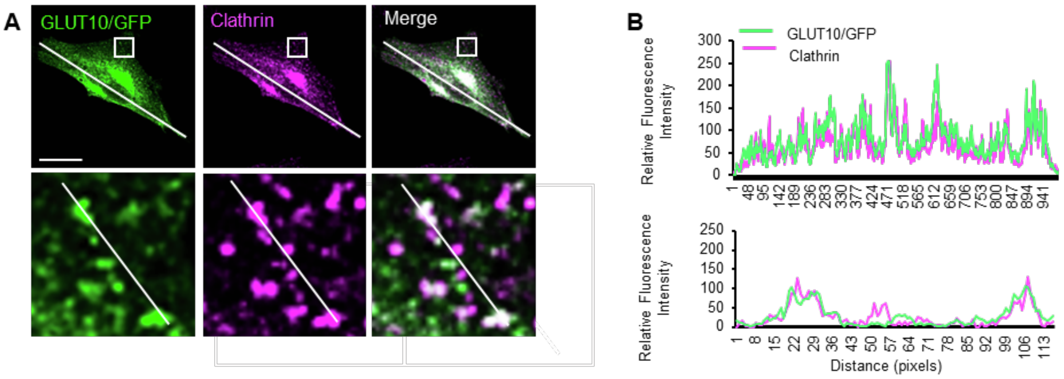

Figure S6. GLUT10 colocalizes with clathrin.

(**A**) Confocal images of GLUT10/GFP colocalized with immunofluorescence (IF)-labeled clathrin in GLUT10/GFP-expressing A10 cells. *Green,* GLUT10/GFP; *Magenta*, clathrin; *white,* merged. Scale bar, 25 µm. (**B**) Intensity plots of line-scan analysis from (**A**) demonstrate the colocalization of GLUT10/GFP and clathrin.

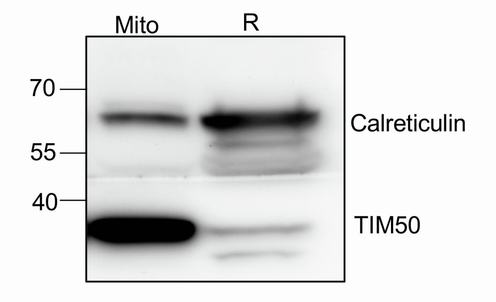

Figure S7. Efficiency of mitochondria isolation kit. Immunoblots of TIM50 and calreticulin protein levels in mitochondria-enriched (Mito) and remaining (R) fractions isolated from GLUT10/V5 expressing T-REx-293 cells using the mitochondria isolation kit. Calreticulin, ER marker; TIM50, mitochondrial marker.

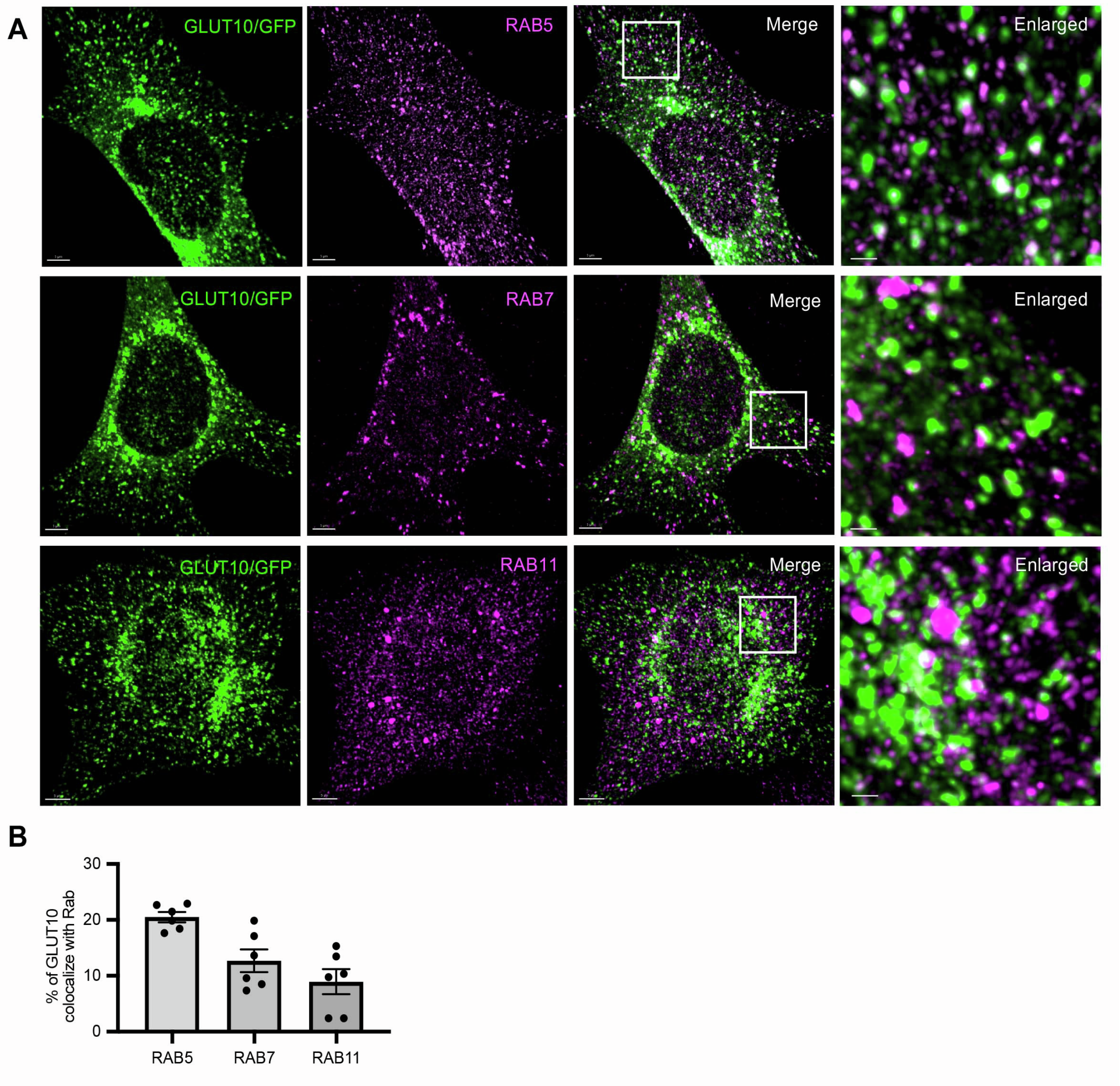

Figure S8. GLUT10 colocalizes with RAB5, RAB7 and RAB11.

(A) Representative confocal images showing colocalization of GLUT10/GFP with RAB5, RAB7 or RAB11 in GLUT10/GFP expressing MOVAS cells. Cells were IF stained for RAB5, RAB7 or RAB11. *Green,* GLUT10/GFP; *magenta*, RAB5, RAB7 or RAB11; *white,* merged. Scale bar, 5 μm. (**B**) Quantification of the percentages of GLUT10/GFP colocalized with RAB5, RAB7 and RAB11 from (**A**). Data are shown as mean ± SEM, n = total 5-6 cells from 3 independent experiments for each group.

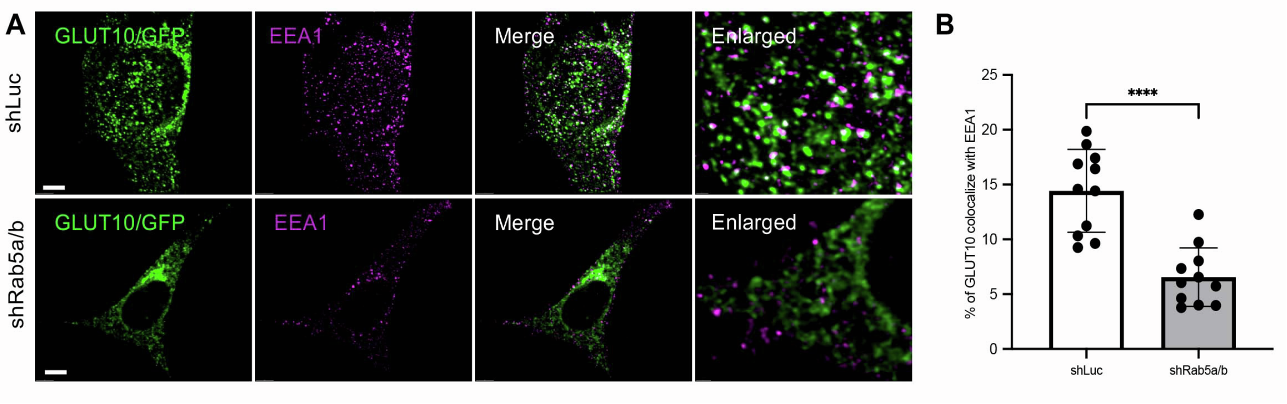

Figure S9. RAB5AB double knockdown reduces GLUT10/GFP colocalization with EEA1 in MOVAS cells.

(**A**) Confocal images showing colocalization of GLUT10/GFP with EEA1 in control (shLuc) and RAB5AB double knockdown (shRab5a/b) MOVAS cells. *Green,* GLUT10/GFP; *magenta*, IF for early endosome marker EEA1; *white,* merged. Scale bar, 5 μm. (**B**) Quantification of the percentage of GLUT10 colocalized with EEA1, as in **A**. Data are presented as mean ± SEM, n = 11cells per group from 3 independent experiments. Statistical significance was determined by two-tailed Student's t-test. ****$P < 0.0001$.

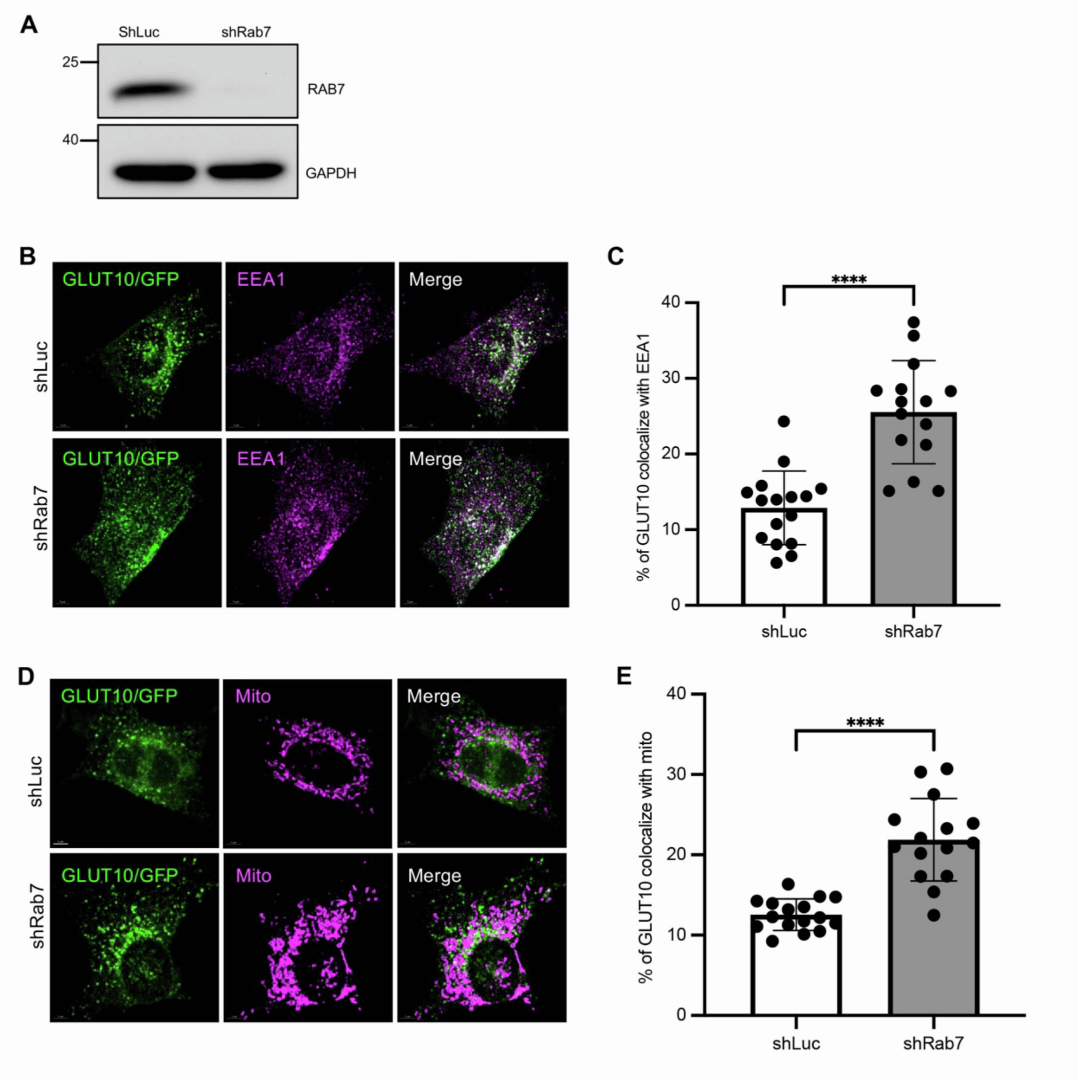

Figure S10. Knockdown of RAB7 causes GLUT10 accumulation in EEs and increase GLUT10 colocalization with mitochondria.

(**A**) Validation of RAB7 knockdown. Immunoblot to analyze of RAB7 levels in control (shLuc) and RAB7 knockdown (shRab7) MOVAS cells. (**B**) Confocal images showing colocalization of GLUT10/GFP with EEA1 in control (shLuc) and RAB7 knockdown (shRab7) MOVAS cells. *Green,* GLUT10/GFP; *magenta*, IF for early endosome marker EEA1; *white,* merged. Scale bar, 5 µm. (**C**) Quantification of the percentage of GLUT10 colocalized with EEA1, as in **B**. Data are shown as mean ± SEM, n = total 10-15 cells for each group from 3 independent experiments. (**D**) Confocal images of GLUT10/GFP colocalized with mitochondria in control (shLuc) and RAB7 knockdown (shRab7) MOVAS cells. *Green,* GFP; *magenta*, IF for mitochondrial marker ATP5A1; *white,* merged. Scale bar, 5 µm. (**E**) Quantification of the percentage of GLUT10/GFP colocalized with mitochondria, as in **D**. Data are shown as mean ± SEM, n = total 10-15 cells for each group from 3 independent experiments. Statistical significance was determined by two-tailed Student's t-test. ****$P < 0.0001$.

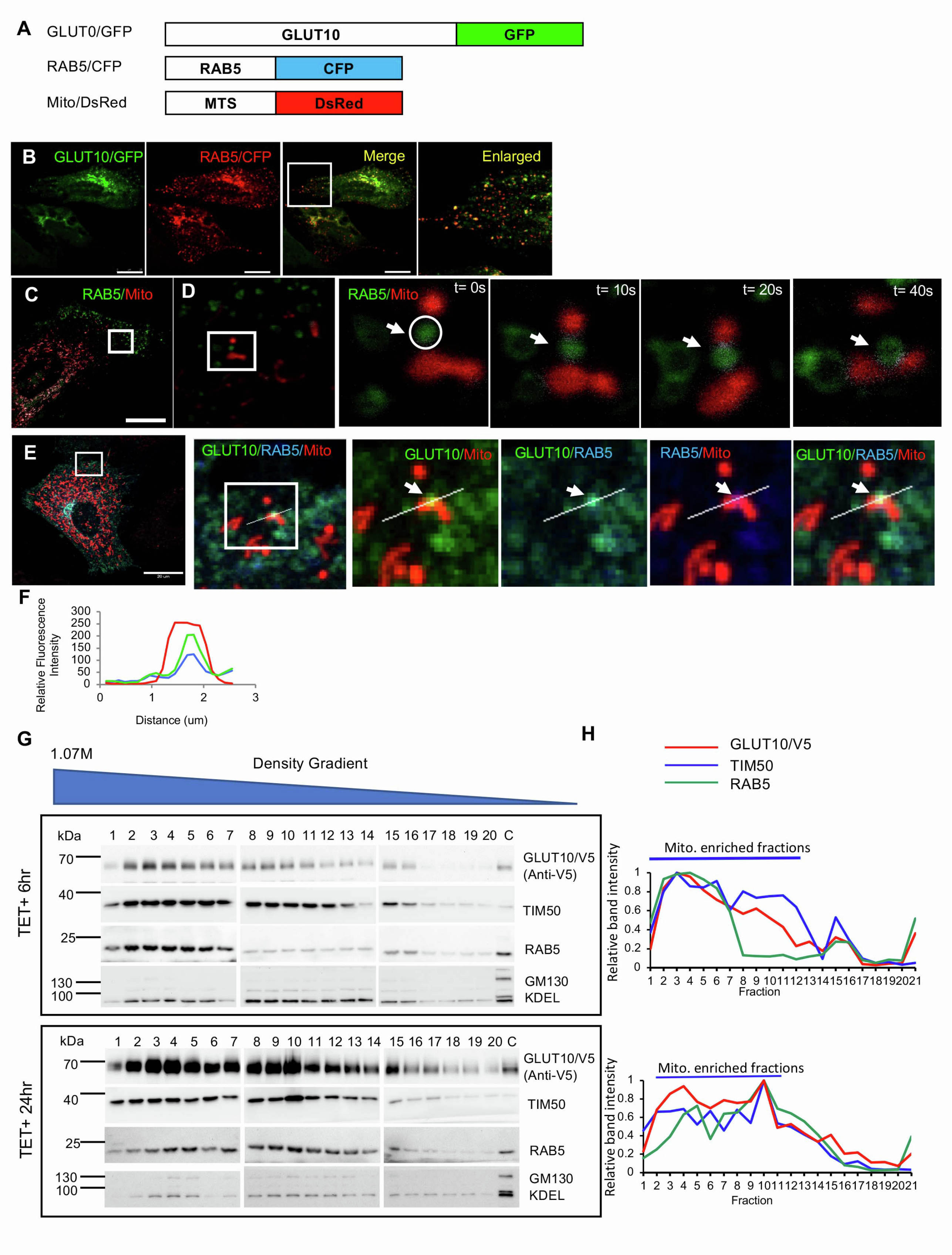

Figure S11. Colocalization of GLUT10 with RAB5 and mitochondria.

(**A**) Schematic representation of expression constructs for GLUT10/GFP, RAB5/CFP and Mito/DsRed fusion proteins. (**B-E**) Confocal images of live A10 cells expressing GLUT10/GFP,

RAB5/CFP, and Mito/DsRed. (**B**) Confocal images showing colocalization of GLUT10 with RAB5. *Green,* GLUT10/GFP; *red*, RAB5/CFP; *yellow,* merged. Scale bar, 10 μm. (**C** and **D**) Confocal images tracking RAB5/CFP and Mito/DsRed. *Green,* RAB5/CFP; *red*, Mito/DsRed. Scale bar, 20 μm. (**D**) Magnified time-lapse confocal images from **C** tracking the targeting of RAB5/CFP to mitochondria in live A10 cells treated with 100 μM H_2O_2, imaged every 10 s from t = 0-40 s. *Green,* RAB5/CFP; *red*, Mito/DsRed; *white,* merged. (**E**) Confocal images and line-scan analyses showing colocalization of GLUT10, RAB5 and mitochondria. *Green,* GLUT10/GFP; *blue,* RAB5/CFP; *red*, Mito/DsRed. Scale bar, 20 μm. (**F**) Intensity plots from the line-scan analyses of the magnified images in **E** demonstrating colocalization of GLUT10/GFP, RAB5/CFP and Mito/DsRed. (**G**) Immunoblots detecting protein levels of GLUT10/V5, TIM50, RAB5, GM130, and KDEL in subcellular organelle fractions (separated on a Percoll density gradient from GLUT10/V5 expressing T-REx-293 cells). Organelle markers: RAB5 for RAB5-positive vesicles; TIM50 for mitochondria; GM130 for Golgi, and KDEL for ER. (**H**) Quantification of the relative intensities of GLUT10/V5, TIM50, and RAB5 in different subcellular organelle fractions from **G**.

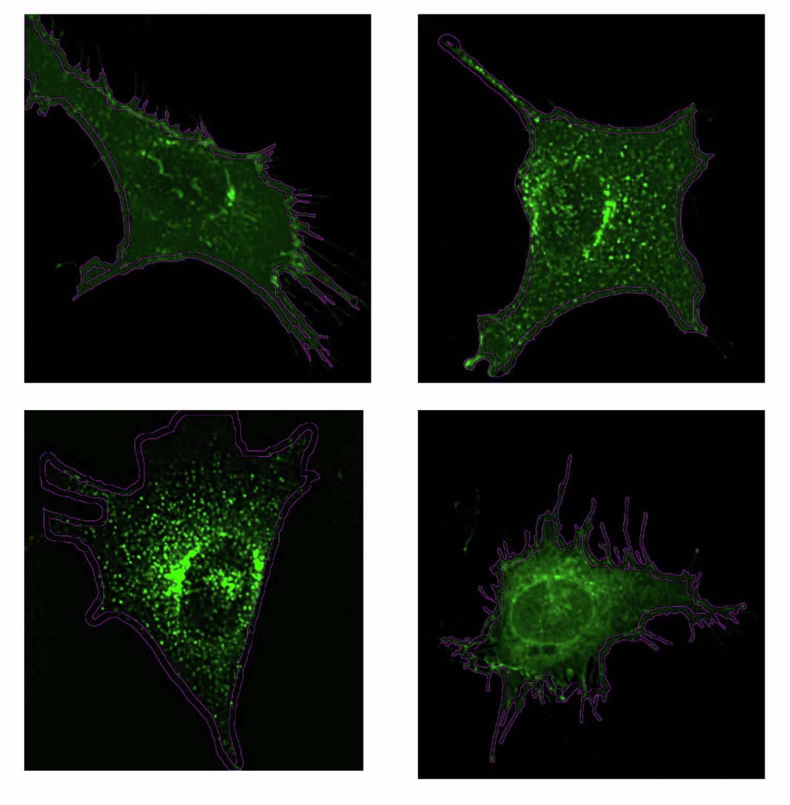

Figure S12. Representative images of defined plasma membrane compartment for GFP intensity calculation. Outer border defines the whole cell. Inner border defines the cytosol. Space between the outer and inner border defines the plasma membrane of the cell. The borders were generated using a custom ImageJ macro, as described in Supplementary materials.

Table S1: Mitochondrial proteins interacting with GLUT10. GLUT10-interacting proteins were identified and annotated as mitochondrial proteins through Gene Ontology (GO) annotation.

No.	Gene	Protein	Sub-mitochondrial localization
1.	*AARS1*	Alanine--tRNA ligase, cytoplasmic	ND
2.	*AARS2*	Alanine--tRNA ligase, mitochondrial	ND
3.	*ABCB7*	Iron-sulfur clusters transporter ABCB7, mitochondrial	MIM
4.	*ABCD3*	ATP-binding cassette sub-family D member 3	ND
5.	*ACAA2*	3-ketoacyl-CoA thiolase, mitochondrial	MM
6.	*ACAD9*	Complex I assembly factor ACAD9, mitochondrial	MIM
7.	*ACADVL*	Very long-chain specific acyl-CoA dehydrogenase, mitochondrial	MIM
8.	*ACAT2*	Acetyl-CoA acetyltransferase, cytosolic	ND
9.	*ACBD3*	Golgi resident protein GCP60	ND
10.	*ACOT7*	Cytosolic acyl coenzyme A thioester hydrolase (Isoform 1)	ND
11.	*ACSL1*	Long-chain-fatty-acid--CoA ligase 1	MOM
12.	*ACSL3*	Long-chain-fatty-acid--CoA ligase 3	MOM
13.	*ACSL4*	Long-chain-fatty-acid--CoA ligase 4	MOM
14.	*ADH5*	Alcohol dehydrogenase class-3	ND
15.	*AFG3L2*	AFG3-like protein 2	MIM
16.	*AGPS*	Alkyldihydroxyacetonephosphate synthase, peroxisomal	ND
17.	*ALDH18A1*	Delta-1-pyrroline-5-carboxylate synthase	MIM
18.	*AP3B1*	AP-3 complex subunit beta-1	ND
19.	*ATP5F1A*	ATP synthase subunit alpha, mitochondrial	MIM
20.	*ATP5F1C*	ATP synthase subunit gamma, mitochondrial	MIM
21.	*ATP7B*	Copper-transporting ATPase 2	ND
22.	*ATPAF1*	ATP synthase mitochondrial F1 complex assembly factor 1	ND
23.	*CAPN1*	Calpain-1 catalytic subunit	ND
24.	*CCAR2*	Cell cycle and apoptosis regulator protein 2	MM
25.	*CDK1*	Cyclin-dependent kinase 1	ND
26.	*CLPX*	ATP-dependent Clp protease ATP-binding subunit clpX-like, mitochondrial	ND
27.	*COASY*	Bifunctional coenzyme A synthase	MM, MOM
28.	*DARS2*	Aspartate--tRNA ligase, mitochondrial	MM
29.	*DDX1*	ATP-dependent RNA helicase DDX1	ND
30.	*DHX30*	ATP-dependent RNA helicase DHX30	Mitochondrial nucleoid
31.	*DHX36*	ATP-dependent DNA/RNA helicase DHX36	ND
32.	*DLAT*	Dihydrolipoyllysine-residue acetyltransferase component of pyruvate dehydrogenase complex, mitochondrial	MM
33.	*DNAJC11*	DnaJ homolog subfamily C member 11	MOM
34.	*ECI2*	Enoyl-CoA delta isomerase 2	ND
35.	*ETFA*	Electron transfer flavoprotein subunit alpha, mitochondrial	MM
36.	*EXD2*	Exonuclease 3'-5' domain-containing protein 2	MOM, MM
37.	*FASTKD2*	FAST kinase domain-containing protein 2, mitochondrial	MM, Mitochondrial nucleoid
38.	*FASTKD5*	FAST kinase domain-containing protein 5, mitochondrial	MM, Mitochondrial nucleoid

39.	*FLVCR1*	Heme transporter FLVCR1 (mitochondria isoform)	Membrane
40.	*GFM1*	Elongation factor G, mitochondrial	MM
41.	*GFM2*	Ribosome-releasing factor 2, mitochondrial	MM
42.	*GJA1*	Gap junction alpha-1 protein	ND
43.	*GLS*	Glutaminase kidney isoform, mitochondrial	MM
44.	*GPN1*	GPN-loop GTPase 1	ND
45.	*GUF1*	Translation factor GUF1, mitochondrial	MIM
46.	*HADHA*	Trifunctional enzyme subunit alpha, mitochondrial	MIM
47.	*HAX1*	HCLS1-associated protein X-1	MM
48.	*HEATR1*	HEAT repeat-containing protein 1	ND
49.	*HIP1R*	Huntingtin-interacting protein 1-related protein	ND
50.	*HSP90AB1*	Heat shock protein HSP 90-beta	ND
51.	*IDH3B*	Isocitrate dehydrogenase [56] subunit beta, mitochondrial	MM
52.	*KARS1*	Lysine--tRNA ligase (Mitochondrial isoform)	MM
53.	*KIFBP*	KIF-binding protein	ND
54.	*KLC2*	Kinesin light chain 2	ND
55.	*LONP1*	Lon protease homolog, mitochondrial	MM
56.	*LRPPRC*	Leucine-rich PPR motif-containing protein, mitochondrial	Mitochondrial nucleoid
57.	*MAPK1*	Mitogen-activated protein kinase 1	ND
58.	*MCCC1*	Methylcrotonoyl-CoA carboxylase subunit alpha, mitochondrial	MM
59.	*MFN1*	Mitofusin 1	MOM
60.	*MIPEP*	Mitochondrial intermediate peptidase	MM
61.	*MRPL38*	Large ribosomal subunit protein mL38	MIM
62.	*MRPL45*	Large ribosomal subunit protein mL45	MIM
63.	*MRPS22*	Small ribosomal subunit protein mS22	MIM, mitochondrial ribosome
64.	*MRPS28*	Small ribosomal subunit protein bS1m	MIM
65.	*MSTO1*	Protein misato homolog 1	MOM
66.	*MTCH2*	Mitochondrial carrier homolog 2	MOM
67.	*MTFR1*	Mitochondrial fission regulator 1	ND
68.	*MTHFD1*	C-1-tetrahydrofolate synthase, cytoplasmic	ND
69.	*MTOR*	Serine/threonine-protein kinase mTOR	MOM
70.	*MTPAP*	Poly(A) RNA polymerase, mitochondrial	ND
71.	*MTX3*	Metaxin-3	MOM
72.	*MUL1*	Mitochondrial ubiquitin ligase activator of NFKB	MOM
73.	*NCBP1*	Nuclear cap-binding protein subunit 1	ND
74.	*NDUFA10*	NADH dehydrogenase [ubiquinone] 1 alpha subcomplex subunit 10, mitochondrial	MIM, MM
75.	*NDUFS1*	NADH-ubiquinone oxidoreductase 75 kDa subunit, mitochondrial	MIM, IMS, MM
76.	*NOL6*	Nucleolar protein 6	ND
77.	*NRDC*	Nardilysin	ND
78.	*PC*	Pyruvate carboxylase, mitochondrial	MM
79.	*PDE2*	cGMP-dependent 3',5'-cyclic phosphodiesterase	MIM, MOM, MM
80.	*PEX5*	Peroxisomal targeting signal 1 receptor	ND
81.	*PGAM5*	Serine/threonine-protein phosphatase PGAM5, mitochondrial	MOM, MIM
82.	*PHB2*	Prohibitin-2	MIM, MOM
83.	*PRKACA*	cAMP-dependent protein kinase catalytic subunit alpha	ND
84.	*PTCD3*	Small ribosomal subunit protein mS39	MIM
85.	*PYCR1*	Pyrroline-5-carboxylate reductase 1, mitochondrial	MM

86.	*PYCR2*	Pyrroline-5-carboxylate reductase 2	MM
87.	*QARS1*	Glutamine--tRNA ligase	MM
88.	*QTRT1*	Queuine tRNA-ribosyltransferase catalytic subunit 1	MOM
89.	*RACK1*	Small ribosomal subunit protein RACK1	ND
90.	*RAF1*	RAF proto-oncogene serine/threonine-protein kinase	MOM
91.	*RAP1GDS1*	Rap1 GTPase-GDP dissociation stimulator 1	ND
92.	*RARS2*	Probable arginine--tRNA ligase, mitochondrial	MM
93.	*RHOT1*	Mitochondrial Rho GTPase 1	MOM
94.	*RHOT2*	Mitochondrial Rho GTPase 2	MOM
95.	*RPS3*	Small ribosomal subunit protein uS3	MIM, MM
96.	*SARM1*	NAD(+) hydrolase SARM1	MOM
97.	*SDHA*	Succinate dehydrogenase [ubiquinone] flavoprotein subunit, mitochondrial	MIM
98.	*SFXN1*	Sideroflexin-1	MIM
99.	*SFXN2*	Sideroflexin-2	MOM/MIM
100.	*SLC25A1*	Tricarboxylate transport protein, mitochondrial	MIM
101.	*SLC25A3*	Solute carrier family 25 member 3	MIM
102.	*SLC25A6*	ADP/ATP translocase 3	MIM
103.	*SLC27A3*	Long-chain fatty acid transport protein 3	Membrane
104.	*SQSTM1*	Sequestosome-1	ND
105.	*STOML2*	Stomatin-like protein 2, mitochondrial	MIM
106.	*SYNE2*	Nesprin-2	ND
107.	*TARS2*	Threonine--tRNA ligase, mitochondrial	MM
108.	*TDRKH*	Tudor and KH domain-containing protein	ND
109.	*TIMM50*	Mitochondrial import inner membrane translocase subunit TIM50	MIM
110.	*TOMM40*	Mitochondrial import outer membrane translocase subunit TOM40	MOM
111.	*TOMM70*	Mitochondrial import outer membrane translocase subunit TOM70	MOM
112.	*TRAP1*	Heat shock protein 75 kDa, mitochondrial	MM, MIM, IMS
113.	*TRUB1*	Pseudouridylate synthase TRUB1	ND
114.	*TSFM*	Elongation factor Ts, mitochondrial	MM
115.	*USP15*	Ubiquitin carboxyl-terminal hydrolase 15	ND
116.	*USP48*	Ubiquitin carboxyl-terminal hydrolase 48	ND
117.	*VARS2*	Valine--tRNA ligase, mitochondrial	ND
118.	*VDAC1*	Voltage-dependent anion-selective channel protein 1	MOM
119.	*VDAC2*	Voltage-dependent anion-selective channel protein 2	MOM
120.	*VDAC3*	Voltage-dependent anion-selective channel protein 3	MOM
121.	*YME1L1*	ATP-dependent zinc metalloprotease YME1L1	MIM

MM, Mitochondrial matrix; MIM, Mitochondrial inner membrane, IMS, Mitochondrial intermembrane space; MOM, Mitochondrial outer membrane; ND, Sub-mitochondrial localization not determined

Table S2. Proteins identified from GLUT10-containing vesicles and analyzed by GO enrichment analysis by g:Profiler: CC terms. A total of 210 identified proteins were analyzed in CC terms.

Term Name<br>Term ID	– log10<br>Adjusted *P* value	Intersections
vesicle<br>GO:CC:0031982	37.19	ACTR10,ACTR1B,AGTRAP,AHCY,ALDH7A1,ALDOA,ANP32E,ANXA2,APPL1,ATG9A,ATP5F1B,ATP6V0A2,ATP6V0D1,AZGP1,BCAP31,BPIFB1,BROX,CACYBP,CANX,CAPZA1,CBR1,CCT6A,CD81,CDK1,CFL2,CLIC1,CMTM6,COPA,COPB1,COPB2,COPE,COTL1,CPD,DNAJC7,EIF3H,ENPP4,FAM3C,FSCN1,GDI2,GGCT,GLO1,GOLPH3,GPI,GPR107,GSTK1,HMGB1,HPRT1,HRNR,HSP90AA1,HSPA8,ITM2B,KIAA0319L,M6PR,MPI,MTHFD1,NAPA,NEBL,OCLN,PCMT1,PDCD5,PDCD6IP,PDIA3,PIP,PPIB,PRDX1,PTPA,RAB1A,RAB1B,RAB2A,RAB2B,RAB31,RAB35,RAB5B,RAB5C,RAB6A,RAB7A,RAB8A,RABAC1,RARS1,RCC2,RPL12,RPN1,S100A7,SCAMP1,SCAMP2,SCAMP4,SLC12A9,SLC1A5,SLC35F6,SLC38A1,SLC9A8,SNAP29,STMN1,STX10,STX12,STX16,STX6,STX7,STX8,SYAP1,TAGLN2,TCP1,TEX264,TFRC,TGOLN2,TKT,TMEM165,TMEM168,TMEM9,TNPO1,TPI1,TXNDC17,USO1,VAMP2,VAMP3,VAMP4,VAMP8,VCP,VPS35,VTI1A,WDR11,YIPF3,YIPF6,YWHAB
endomembrane system<br>GO:CC: 0012505	19.12	ACTR10,ACTR1B,AGTRAP,AIMP1,ALDOA,ANXA2,APPL1,ARFGAP1,ATG9A,ATP2C1,ATP6V0A2,ATP6V0D1,BCAP31,BPNT2,BROX,CACYBP,CANX,CDK1,CLCC1,CLIC1,CMTM6,COPA,COPB1,COPB2,COPE,COTL1,ENPP4,ERLIN1,ERLIN2,FAM3C,GDI2,GOLM1,GOLPH3,GPI,GPR107,GPR108,HMGB1,HRNR,HSP90AA1,HSPA8,ITM2B,KIAA0319L,M6PR,NOSIP,NSF,PARP1,PDCD6IP,PDIA3,PDXDC1,PPIB,RAB1A,RAB1B,RAB2A,RAB2B,RAB31,RAB35,RAB5B,RAB5C,RAB6A,RAB7A,RAB8A,RABAC1,RCC2,RPN1,RTCB,S100A7,SCAMP1,SCAMP2,SCAMP4,SLC35A2,SLC35B2,SLC35C2,SLC9A8,SNAP29,STIP1,STX10,STX12,STX16,STX6,STX7,STX8,SYAP1,TCP1,TEX264,TFRC,TGOLN2,TKT,TMEM115,TMEM165,TMEM168,TMEM87A,TMEM9,TMX1,TPPP,UBIAD1,UNC45A,USO1,VAMP2,VAMP3,VAMP4,VAMP7,VAMP8,VCP,VPS35,VTI1A,WDR11,YIPF3,YIPF4,YIPF6
SNARE complex<br>GO:CC:0031201	12.50	NAPA,SNAP29,STX10,STX12,STX16,STX6,STX7,STX8,VAMP2,VAMP3,VAMP4,VAMP8,VTI1A
transport vesicle<br>GO:CC:0030133	10.69	ATP6V0D1,COPA,COPB1,COPB2,COPE,M6PR,RAB1A,RAB1B,RAB5B,RAB6A,RAB7A,RAB8A,RABAC1,SCAMP1,STX12,STX16,STX6,STX7,TGOLN2,TMEM168,USO1,VAMP2,VAMP3,VAMP4,VTI1A,YIPF3
Endosome<br>GO:CC:0005768	10.17	ANXA2,APPL1,ATG9A,ATP6V0A2,ATP6V0D1,CMTM6,GOLPH3,GPR107,HMGB1,HSPA8,ITM2B,M6PR,PDCD6IP,PDIA3,RAB1A,RAB31,RAB35,RAB5B,RAB5C,RAB7A,RAB8A,RCC2,SCAMP1,SCAMP2,SCAMP4,SLC9A8,STX12,STX6,STX7,STX8,TFRC,TGOLN2,TMEM165,TMEM9,VAMP3,VAMP4,VAMP8,VPS35,VTI1A
endocytic vesicle<br>GO:CC:0030139	9.18	APPL1,ATP6V0A2,ATP6V0D1,HSP90AA1,M6PR,OCLN,PDIA3,RAB31,RAB35,RAB5B,RAB7A,RAB8A,STX12,STX6,STX7,STX8,TFRC,TGOLN2,VAMP2,VAMP3,VAMP4,VAMP8

clathrin-coated vesicle GO:CC:0030136	7.10	ATP6V0D1,BCAP31,GPR107,HSPA8,M6PR,RAB35,RAB8A,SCAMP1,STX6,TFRC,TGOLN2,VAMP2,VAMP3,VAMP4,VAMP8,VTI1A
recycling endosome GO:CC:0055037	6.49	ATG9A,CMTM6,PDIA3,RAB35,RAB8A,SCAMP1,SCAMP2,SCAMP4,STX12,STX6,STX7,STX8,TFRC,VAMP3,VAMP8
early endosome GO:CC:0005769	4.98	ANXA2,APPL1,ATP6V0D1,CMTM6,GPR107,RAB1A,RAB31,RAB5B,RAB5C,RCC2,STX12,STX6,STX7,STX8,TFRC,TMEM165,VAMP3,VAMP8,VPS35

Table S3. YXXΦ-type signals and subcellular compartment targeting.

Targeting	Sequence	Protein
Receptor internalization	[a]4–19-YXXΦ-10–40-Tm	Transferrin receptor, asialoglycoprotein receptor H1, GLUT4
Lysosomal-endosomal membrane	Tm6–9-YXXΦ	CD164, LAMP3
Lysosomal-endosomal membrane (antigen presenting)	Tm6–9-YXXΦ-1	CD1, CD1b, CD1c, CD1d
Intracellular receptor sorting	Tm25–43-YXXΦ-16-132	CI-MPR, CD-MPR
TGN-endosome	Tm26-YXXΦ-4	TGN38
Endosome	Tm36-YXXΦ-6	GLUT10

[a] Numbers denote amino acid residues before and after the YXXΦ motif. Tm, transmembrane domain; YXXΦ, phenylalanine/tyrosine-based motif; GLUT4, glucose transporter 4; LAMP3, lysosomal-associated membrane protein 3; CI-MPR, cation-independent mannose-6-phosphate receptor; CD-MPR, cation-dependent mannose-6-phosphate receptor; TGN38, *trans*-Golgi network 38. The organization of this table is based on a review by Bonifacino and Traub [27].

Table S4: Oligonucleotide primers used in the study

Target	Oligonucleotide sequence
Mouse *Slc2a10* amplification for GLUT10/GFP construction:	Forward, 5'-CTC GAG ATG GGC CTT CGC CCA GCT GTC CT-3'<br>Reverse, 5'-GGA TCC GAG GAG GCT GAG GAG ACA TC-3'
Signal peptide amplification for SP/GFP/KDEL construction:	Forward, 5'-GAT CTC GAG ATG GGC CTT CGC CCA GCT GTC CT-3'<br>Reverse, 5'-GCA AGG ATC CCC GAA GTT CAG CTG GAG TGG-3'
KDEL-tagged GFP:	Forward, 5'-CGG GAT CCA CCG GTC GCC ACC-3'<br>Reverse, 5'-GCT GAT TAT GAT GCG GCC GCT TAG AGT TCA TCC TTG TAC AGC TC-3'
Mouse *Slc2a1* amplification for GLUT1/GFP construction:	Forward, 5'-CTC GAG ATG GAT CCC AGC AGC AAG AAG-3'<br>Reverse, 5'-GAA TTC GCA CTT GGG AGT CCG CCC C-3'
Mouse *Slc2a1* fragment 892 bp to 915 bp amplification:	Forward, 5'-CTGAAG AAGCTT CGAGGGACAGCC-3'<br>Reverse, 5'-TTTGGTTTCAGGAAC TTTGAAGTAGG-3'
Mouse *Slc2a10* fragment 1548 bp to 1697 bp amplification:	Forward, 5'-TTC ACC TAC TTC AAA GTT CCT GAA ACC AAA GGA CAG-3'<br>Reverse, 5'-GC GAATTC G GGA GGC TGA GGA GAC ATC CAG-3'
Rat *Rab5* cDNA amplification for RAB5/CFP construct	Forward: 5'-CCGGAATTCCATGGCTAATCGAGGAG-3'<br>Reverse: 5'-CGCGGATCCTTAGTTACTACAACACT-3'
GLUT10d/GFP construction:	Forward, 5'-CGC ATC GGT ATT CAG TCG GAT CCA CCG GTC-3'<br>Reverse, 5'-GAC CGG TGG ATC CGA CTG AAT ACC GAT GCG-3'
