# Supplementary material for: Plasma membrane mediated GLUT10 mitochondrial targeting regulates intracellular ascorbic acid homeostasis
Source: iScience. 2026 Apr 25;29(6):115891. doi: 10.1016/j.isci.2026.115891 (PMC13196150; doi:10.1016/j.isci.2026.115891)

All the western blot images used in the manuscript are cropped directly from the raw data captured from the UVP imaging system

Figure 2A-F

TEM images in the .dm3 file format opened in the imageJ and adjusted the brightness and contrast using imageJ

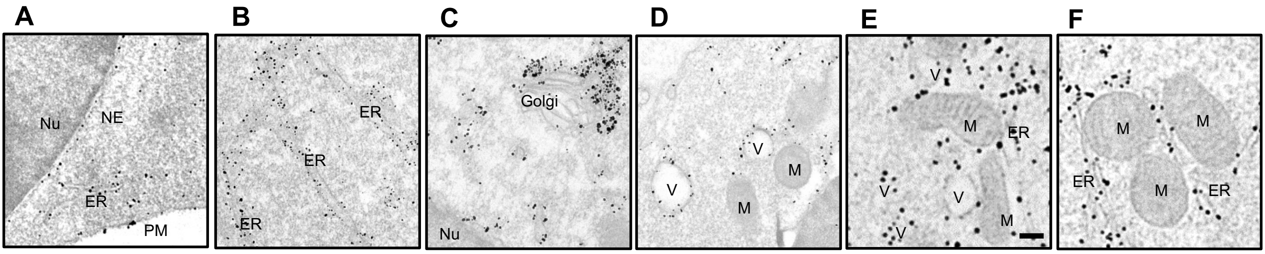

Image 2A

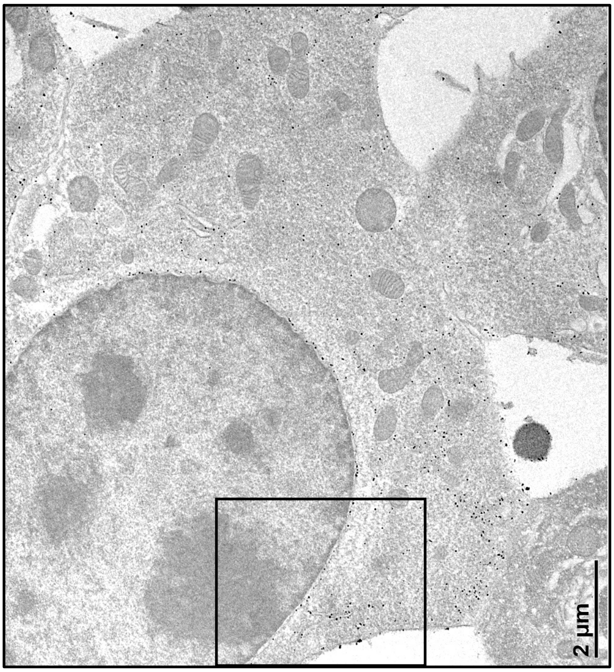

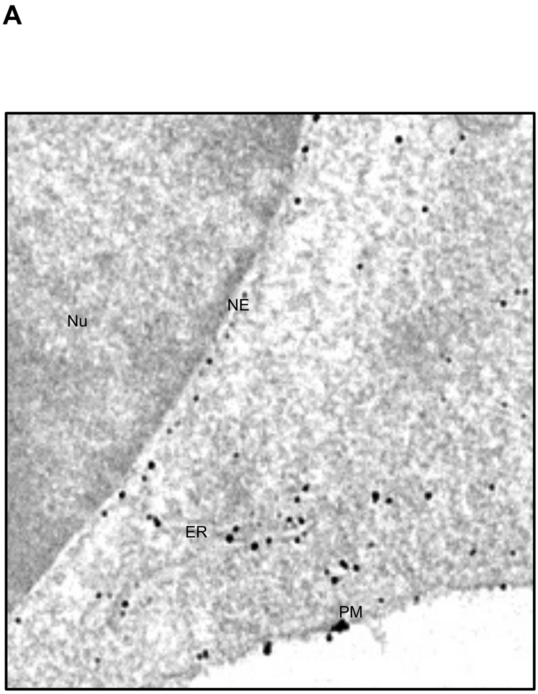

Image 2B

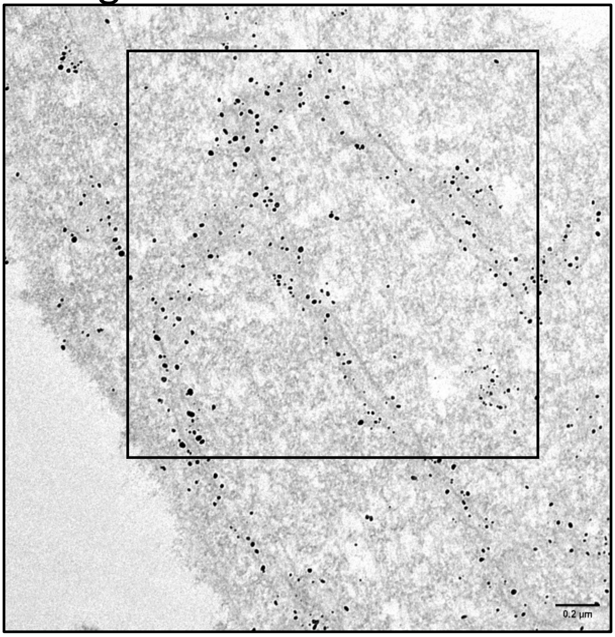

B

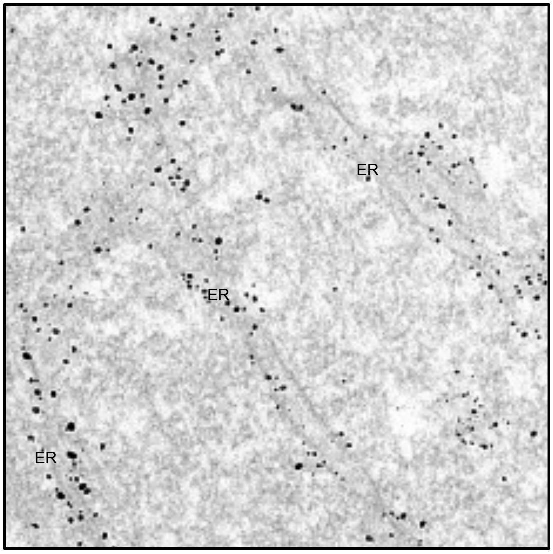

Image 2C

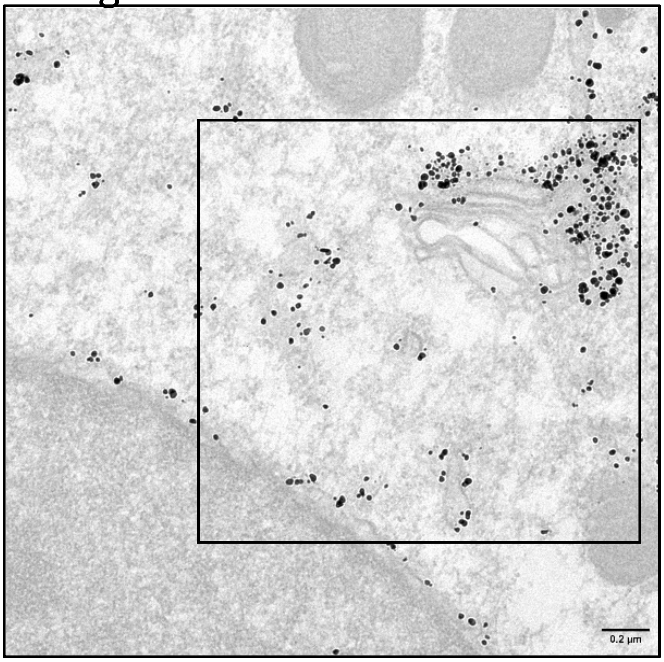

C

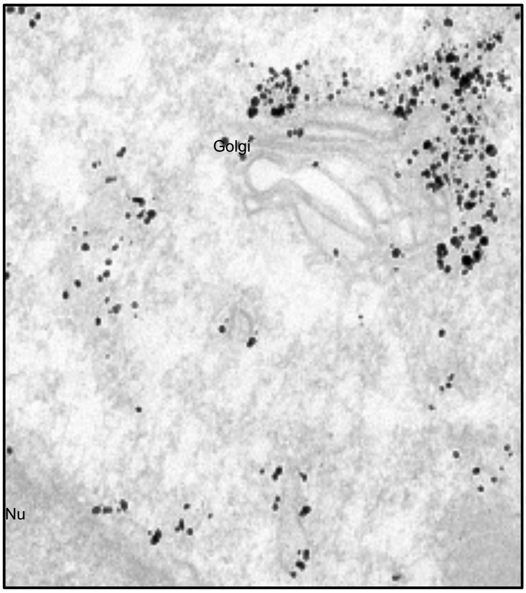

Image 2D

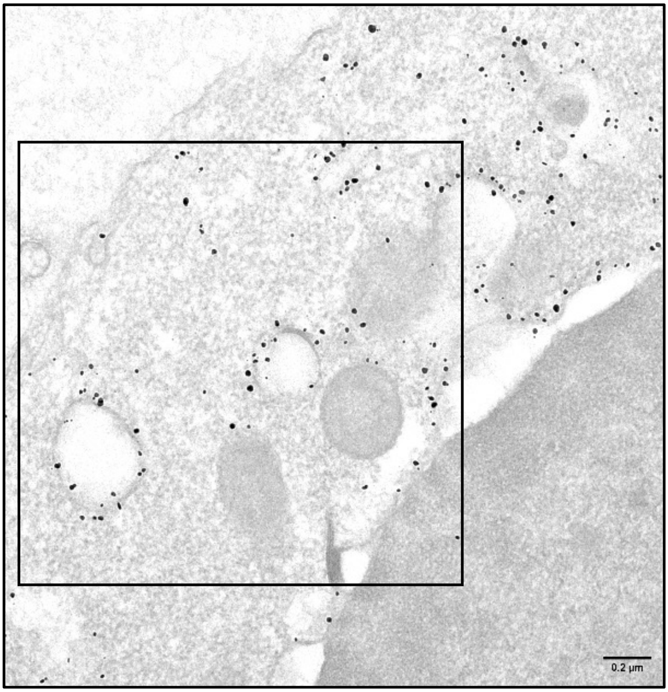

D

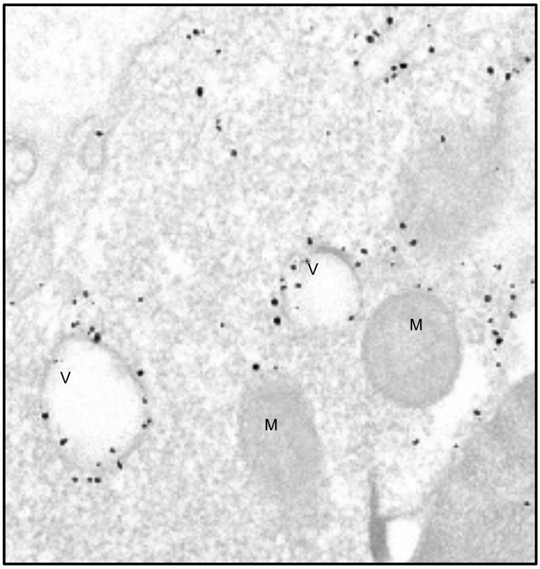

Image 2E

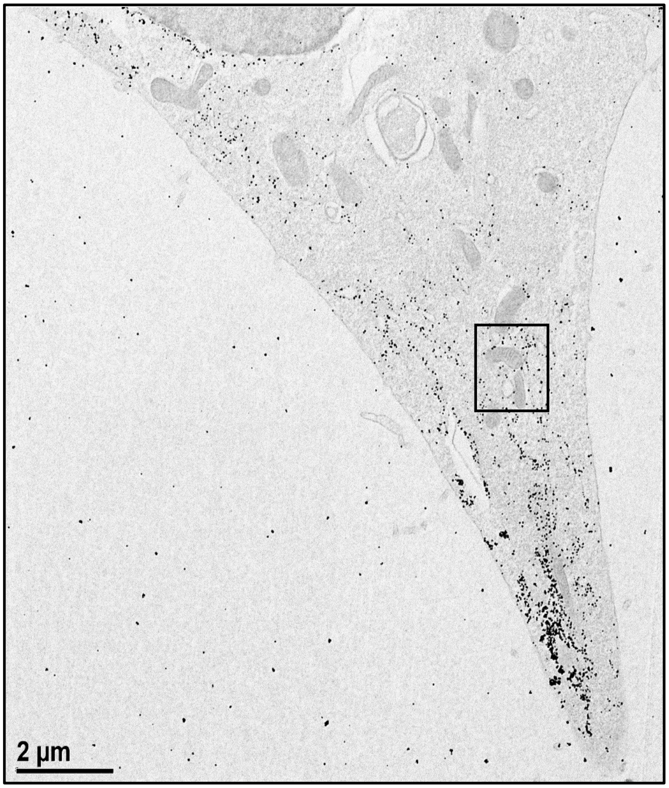

E

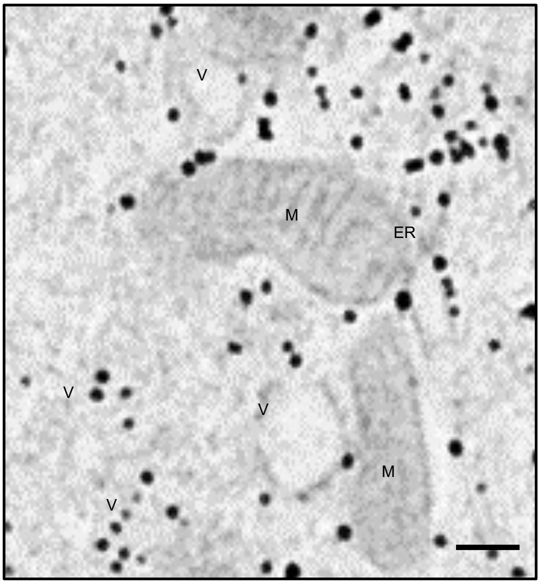

Image 2F

F

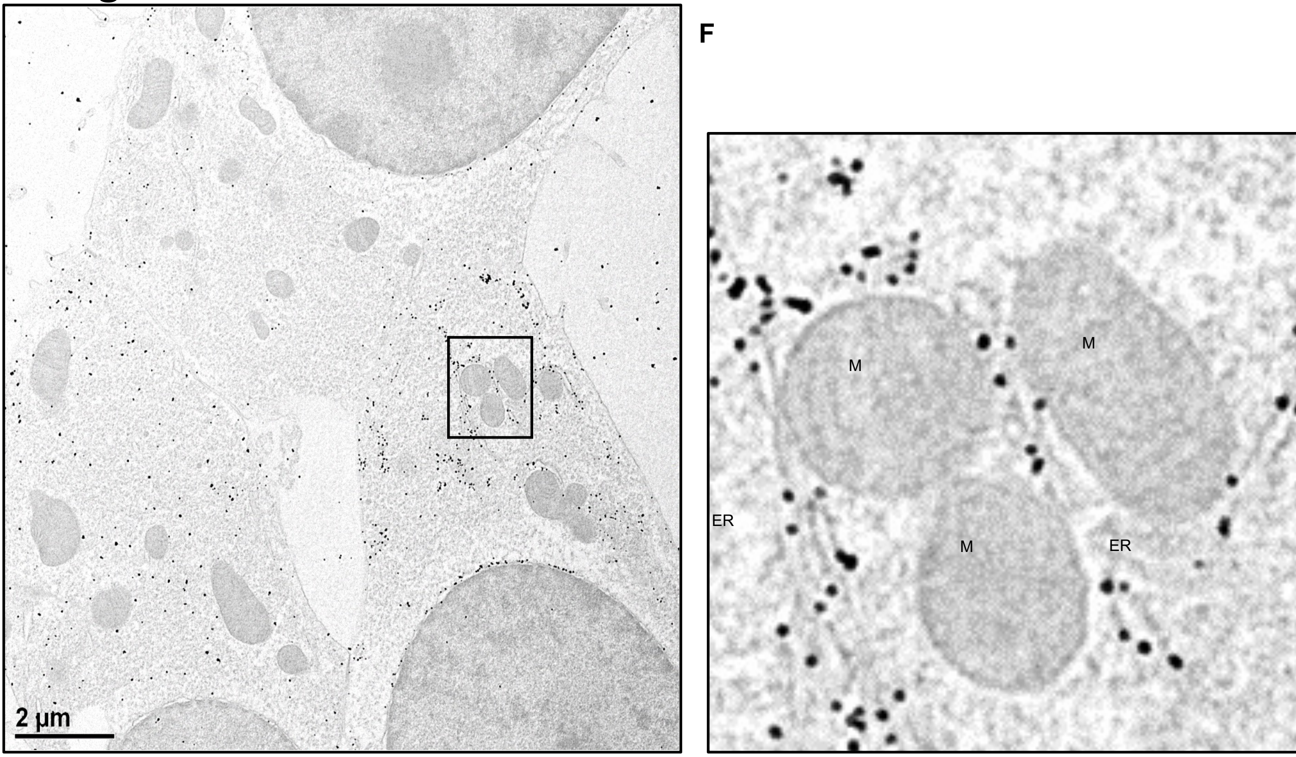

Fig 2M (GLUT10/V5 and TIM50)

RAW IMAGES OF MARKER AND PROTEIN BLOTS

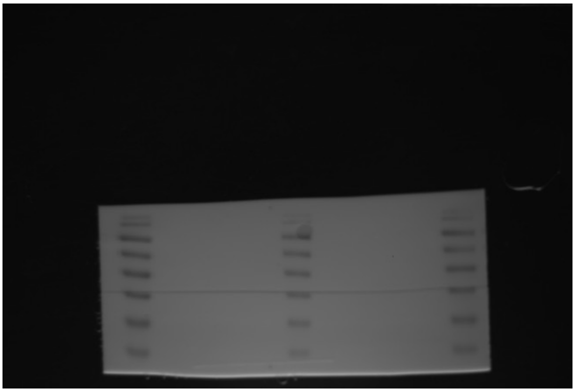

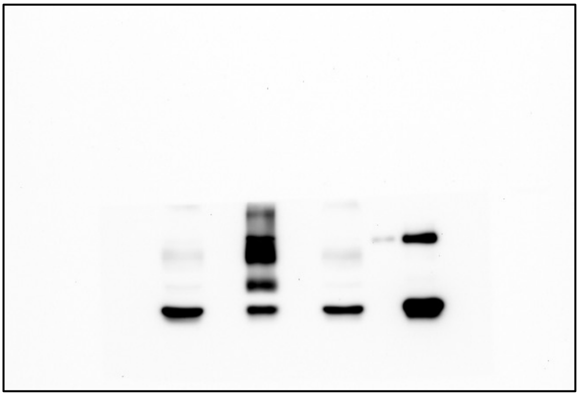

MERGED IMAGES OF MARKER AND PROTEIN

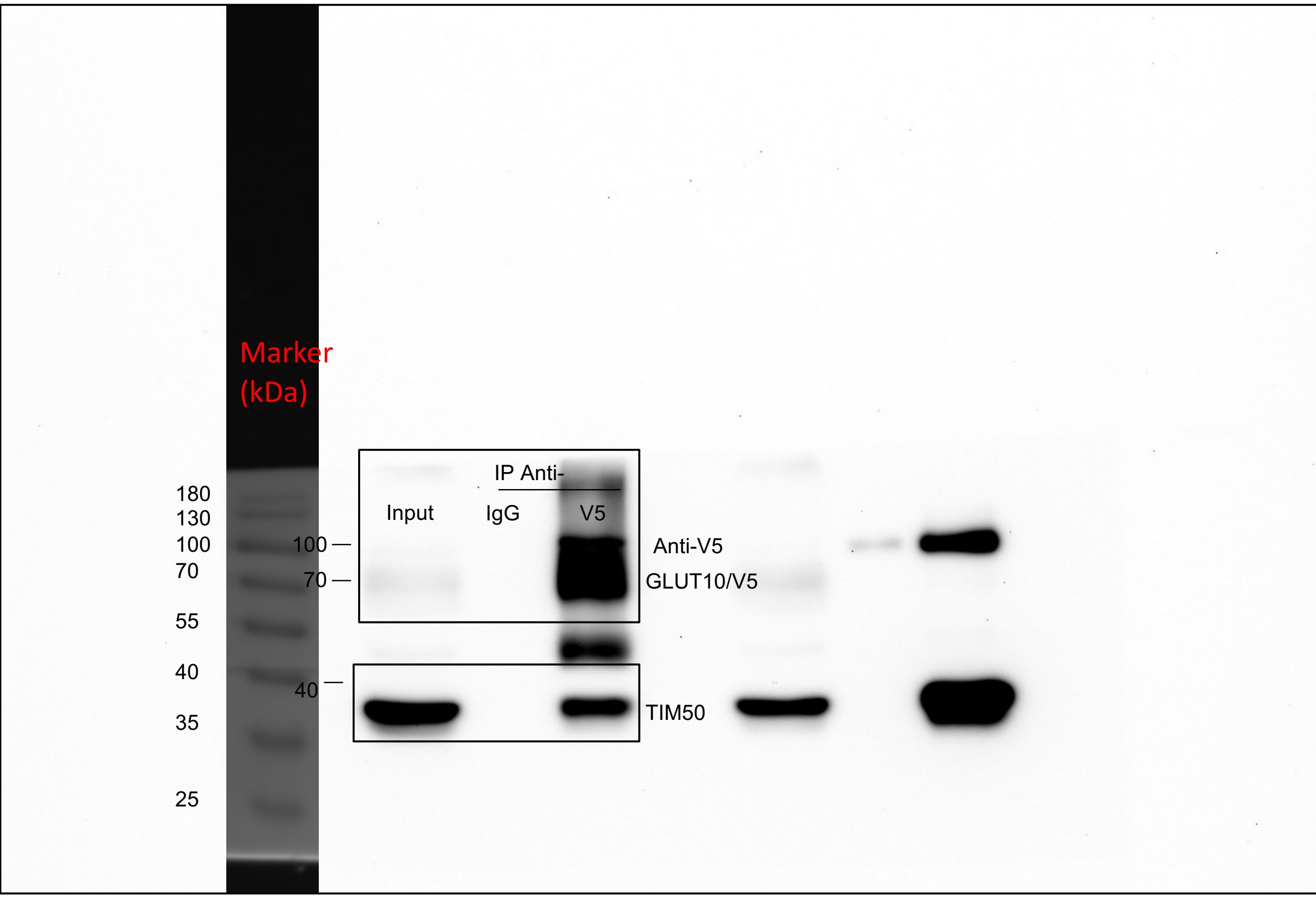

Fig 2M (GLUT10/V5 and TIM50) Replicate

RAW IMAGES OF PROTEIN BLOTS WITH MARKED kDa

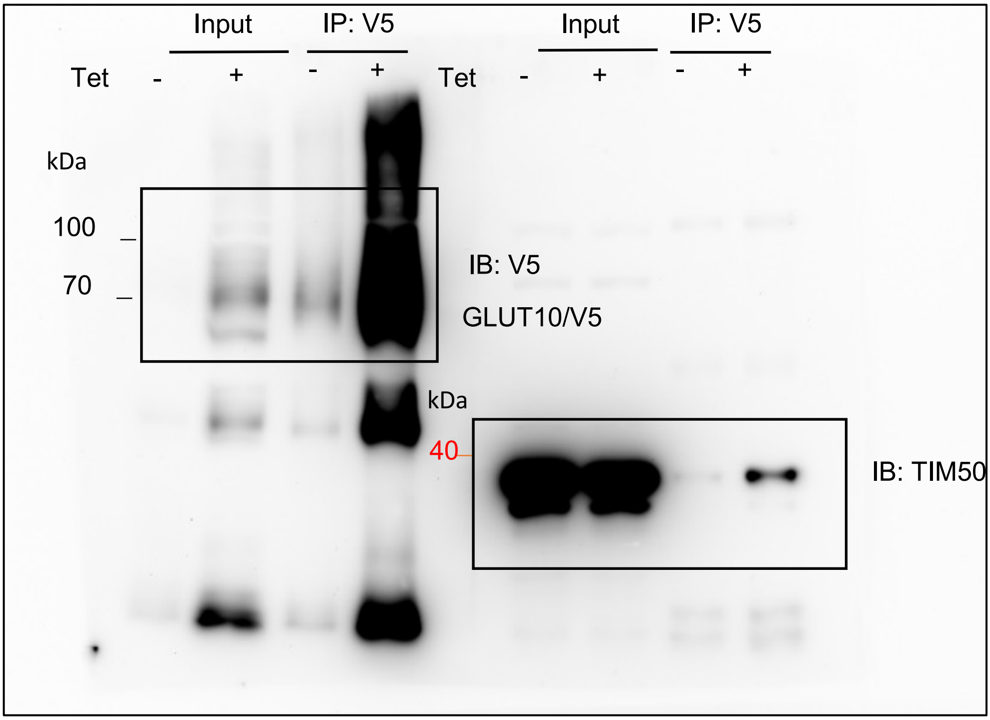

Fig 2N (GLUT10/V5)

RAW IMAGES OF MARKER AND PROTEIN BLOTS

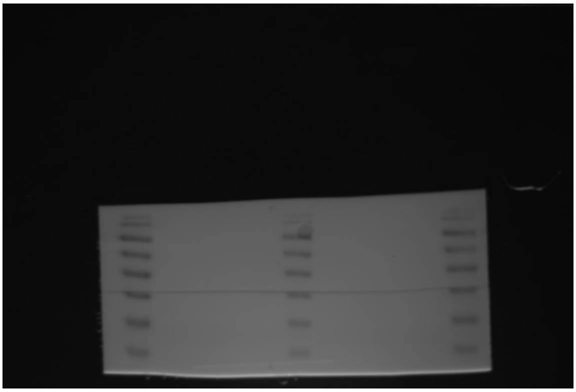

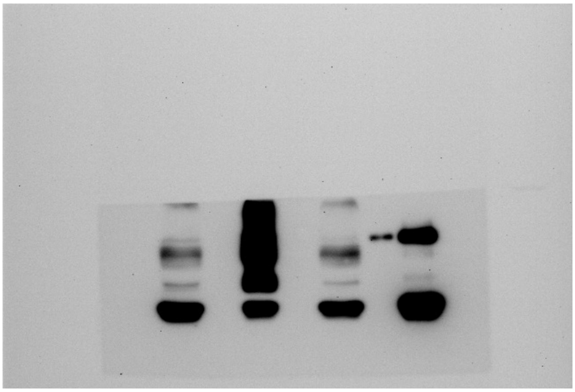

MERGED IMAGES OF MARKER AND PROTEIN

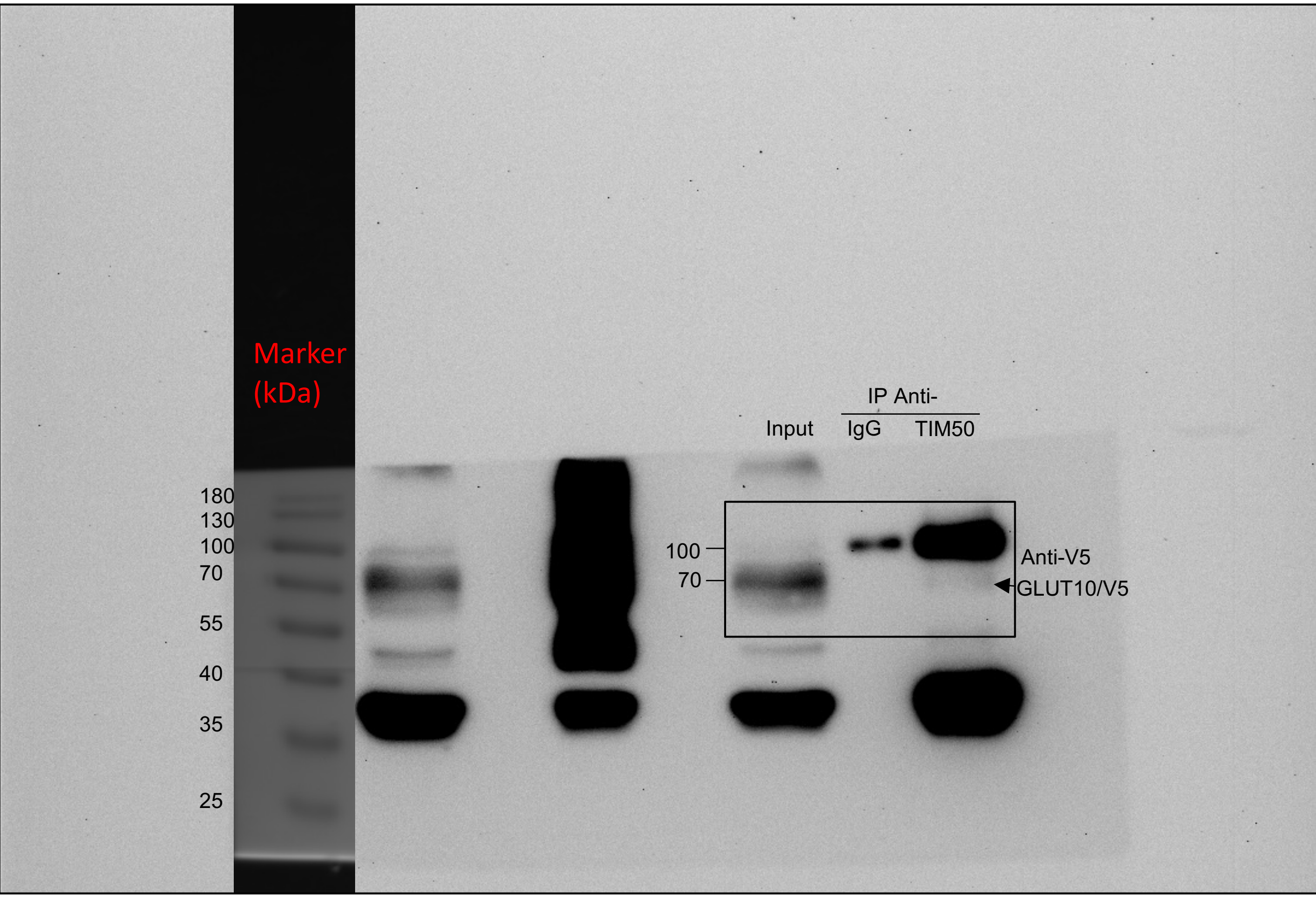

Fig 2N (TIM50)

RAW IMAGES OF MARKER AND PROTEIN BLOTS

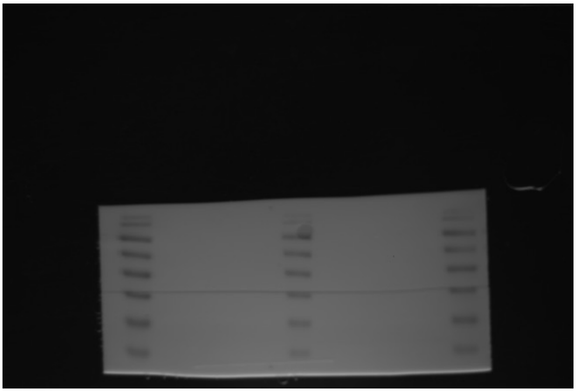

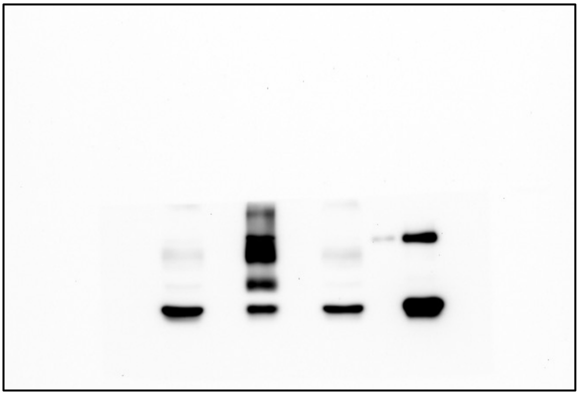

MERGED IMAGES OF MARKER AND PROTEIN

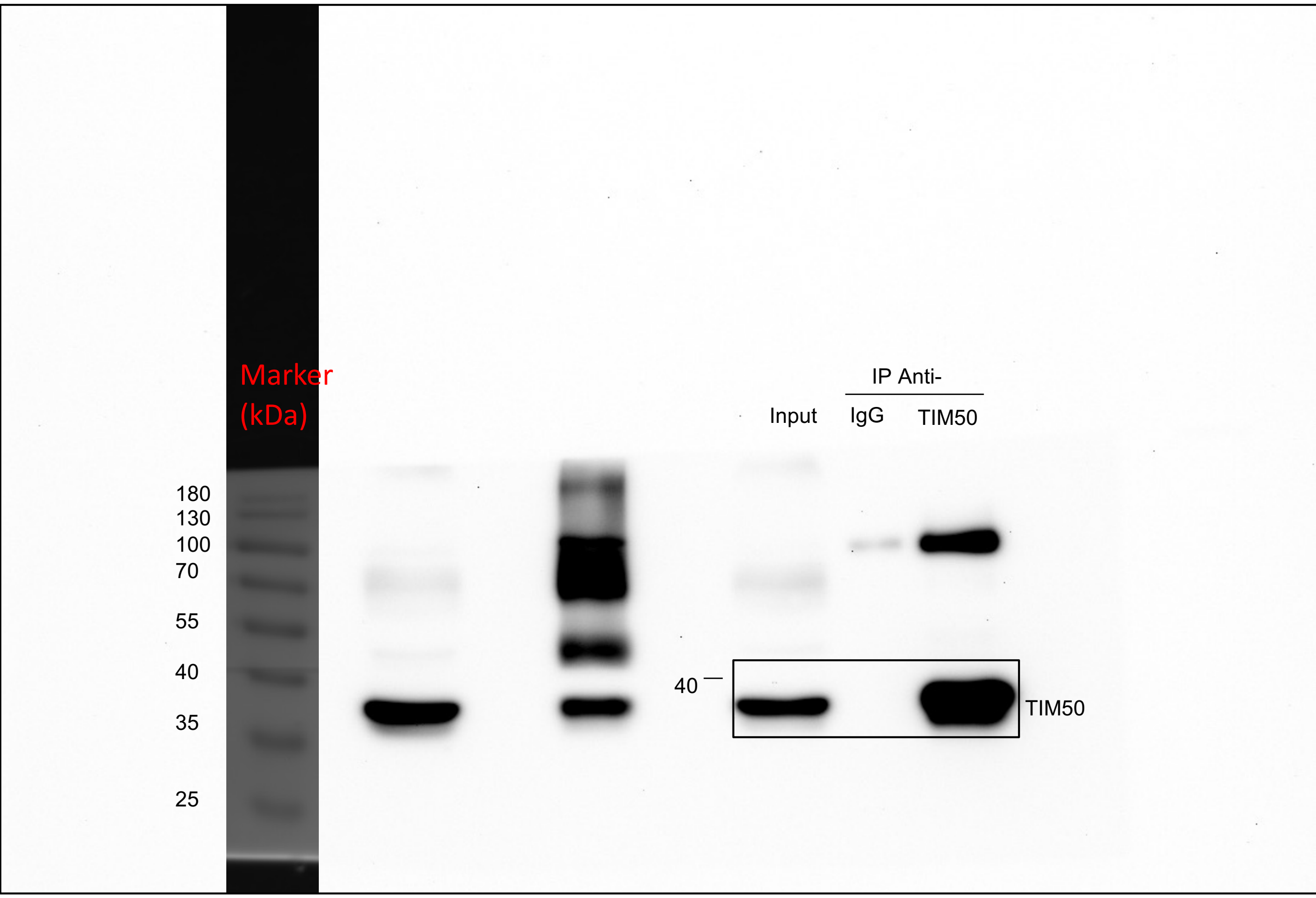

Fig 2N (GLUT10/V5 and TIM 50) Replicate

RAW IMAGES OF PROTEIN BLOTS WITH MARKED kDa

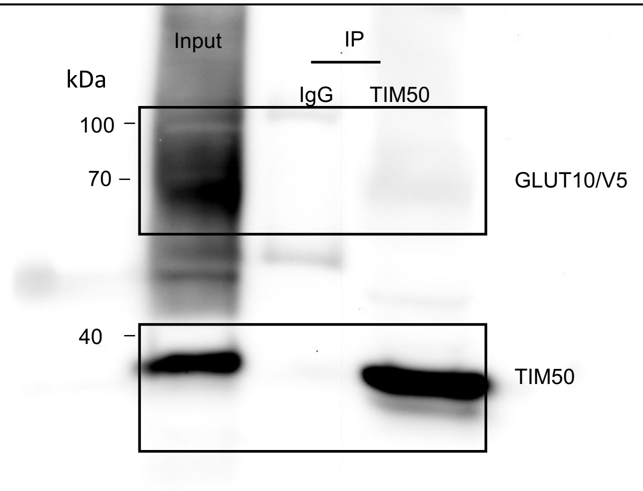

Fig 2O (hGLUT10/TOM20)

RAW IMAGES OF MARKER AND PROTEIN BLOTS

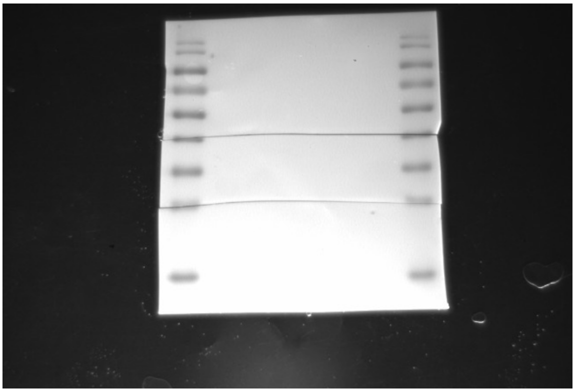

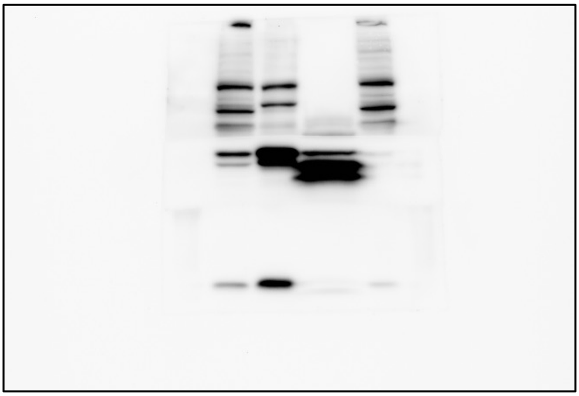

MERGED IMAGES OF MARKER AND PROTEIN

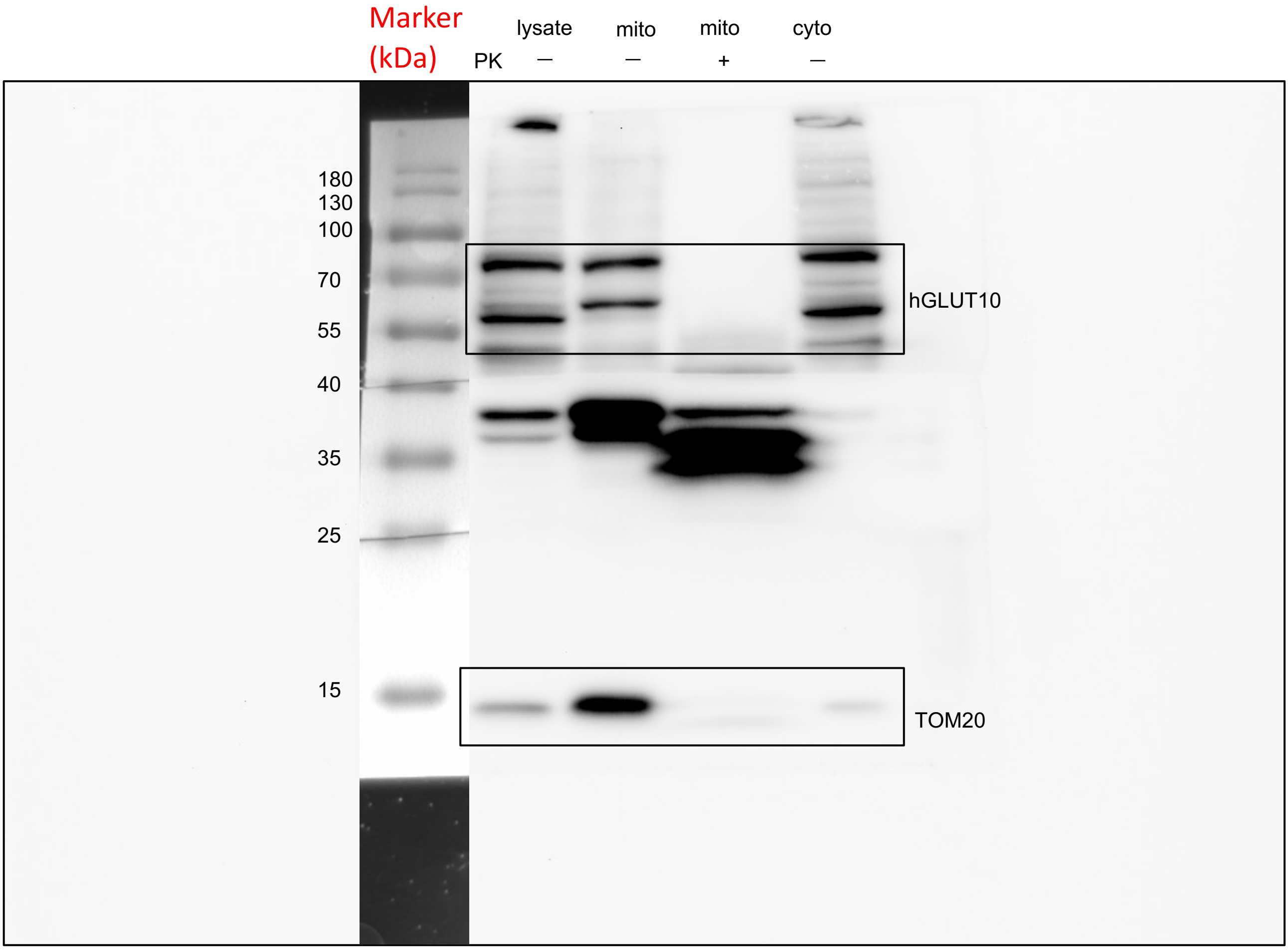

Fig 2O (hGLUT10) (replicate)

RAW IMAGES OF PROTEIN BLOTS with marker

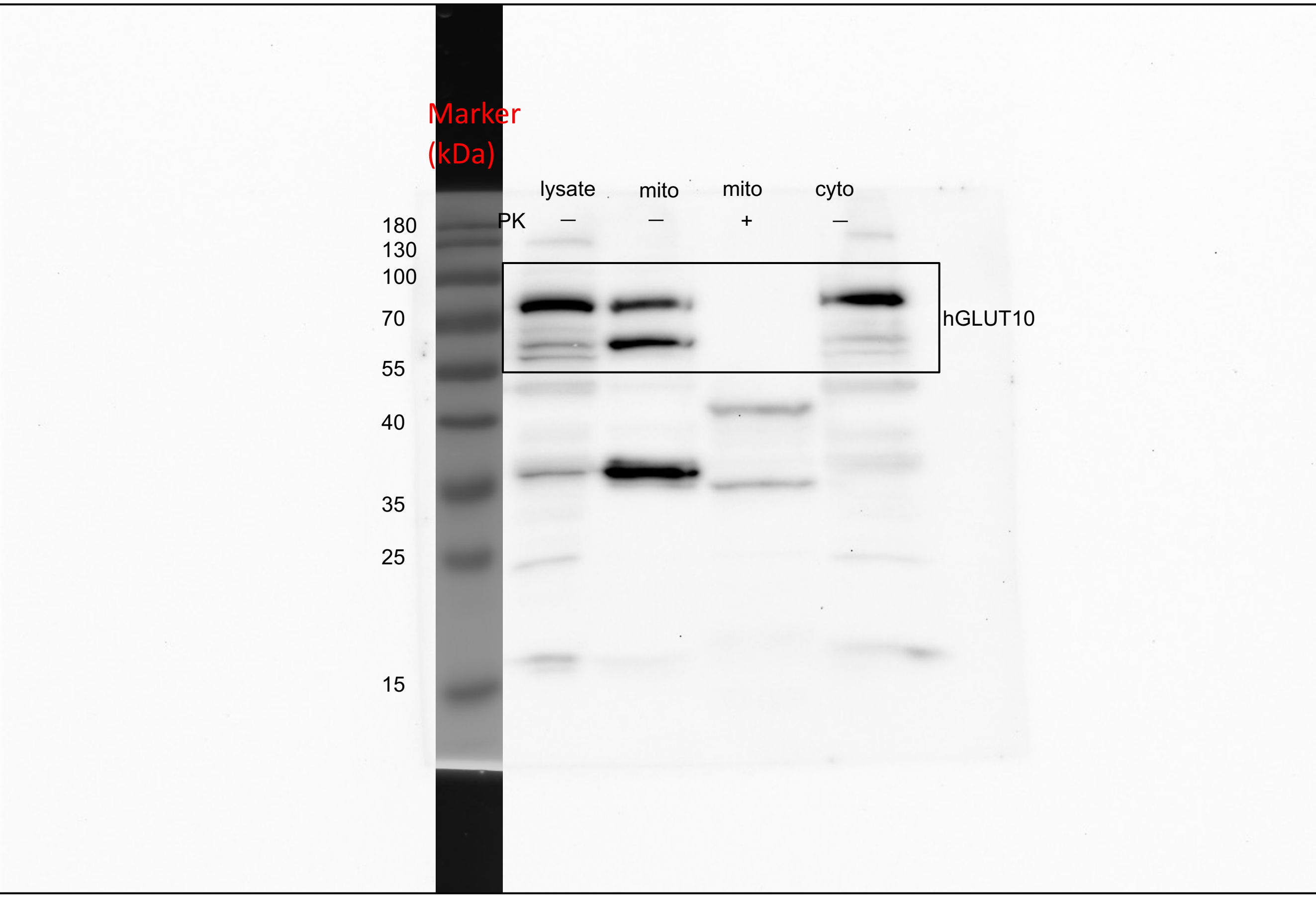

Fig 2O (ATP5A1)

RAW IMAGES OF MARKER AND PROTEIN BLOTS

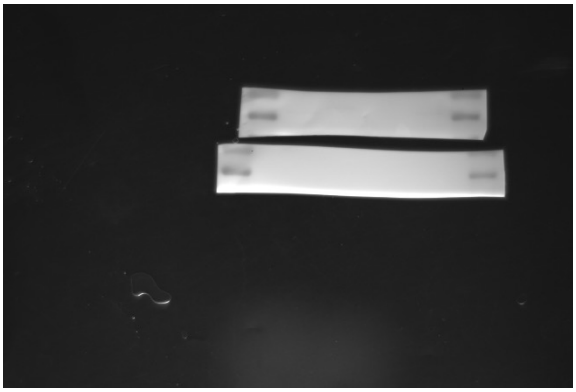

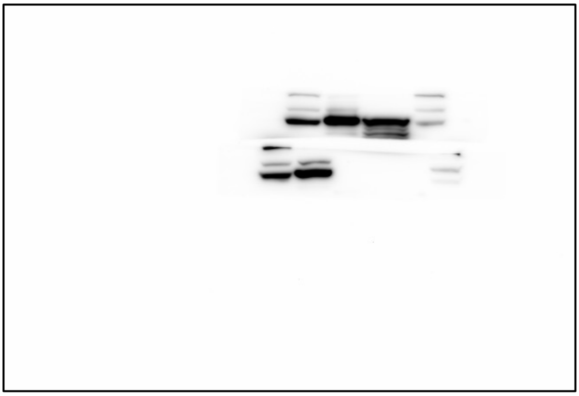

MERGED IMAGES OF MARKER AND PROTEIN

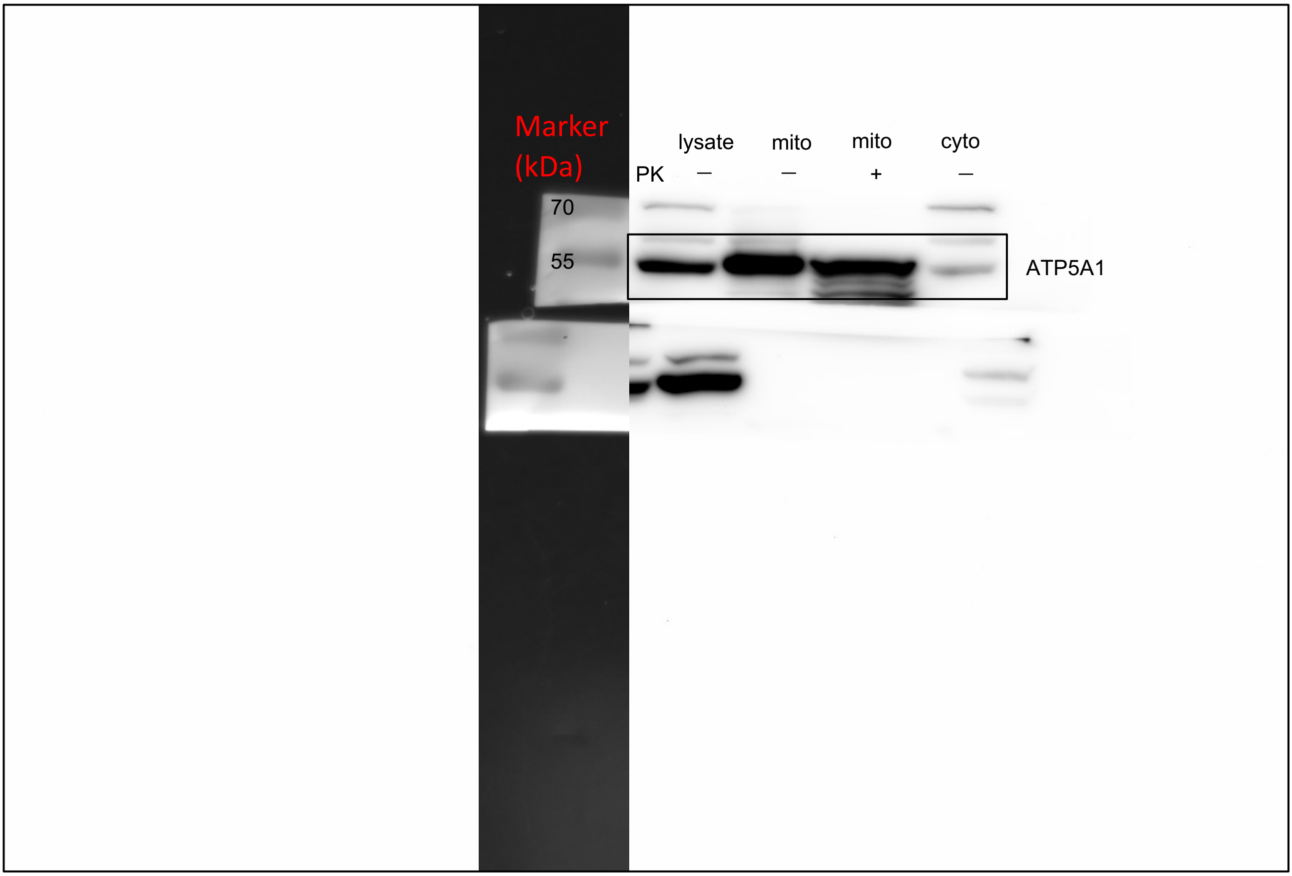

Fig 2O (Calnexin)

RAW IMAGES OF MARKER AND PROTEIN BLOTS

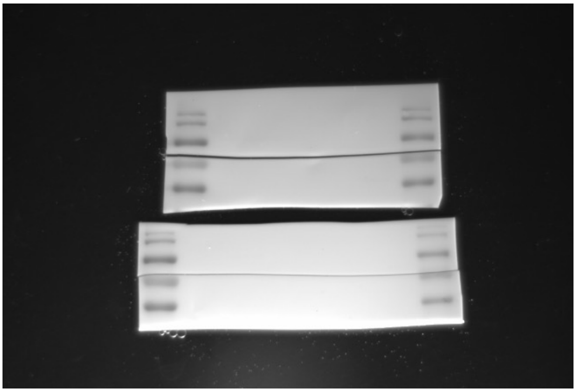

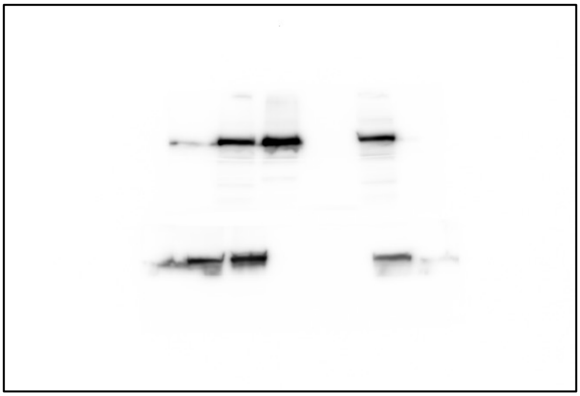

MERGED IMAGES OF MARKER AND PROTEIN

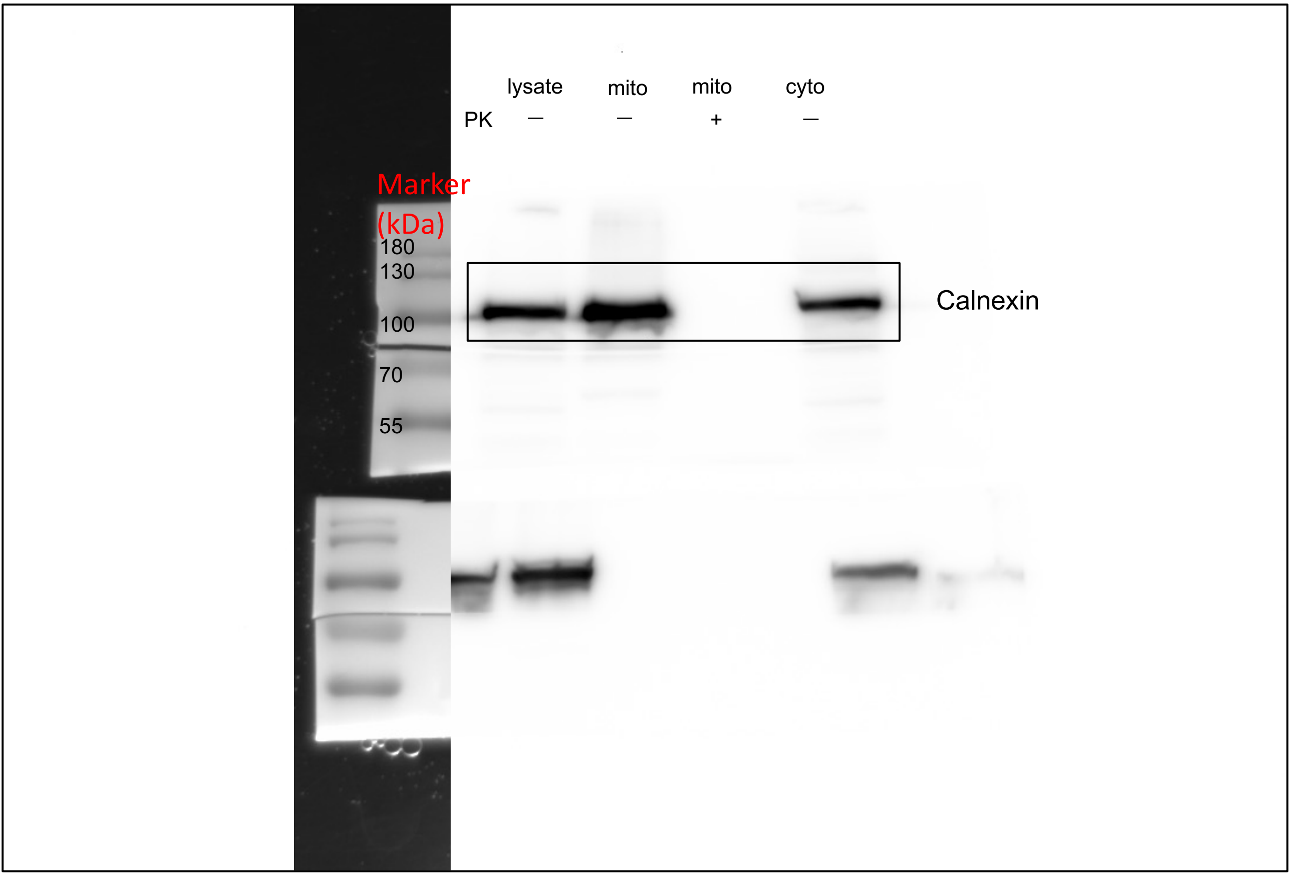

Fig 2P (GLUT10/V5)

RAW IMAGES OF MARKER AND PROTEIN BLOTS

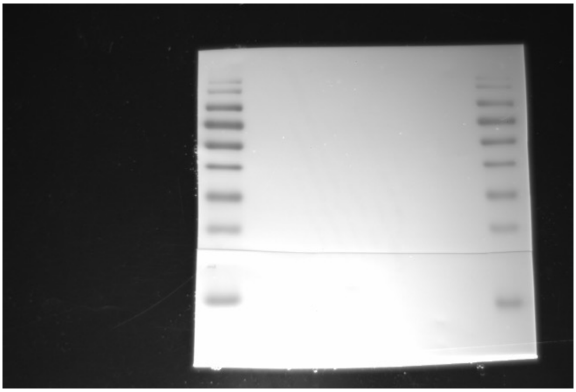

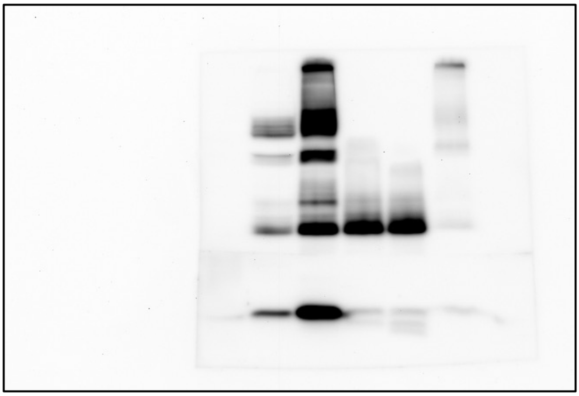

MERGED IMAGES OF MARKER AND PROTEIN

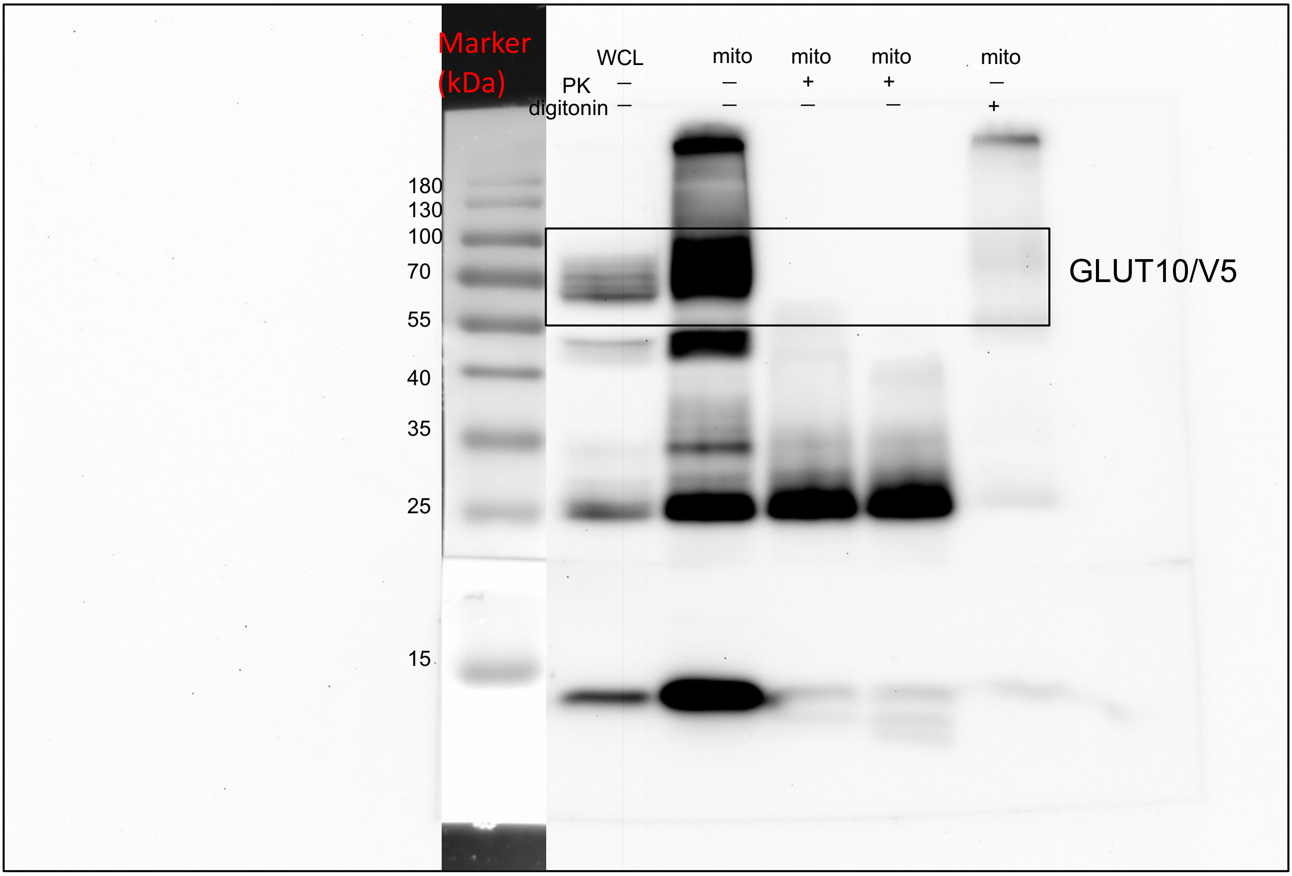

Fig 2P (TOM20)

RAW IMAGES OF MARKER AND PROTEIN BLOTS

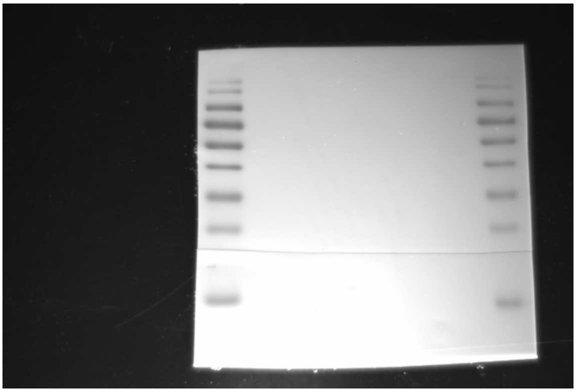

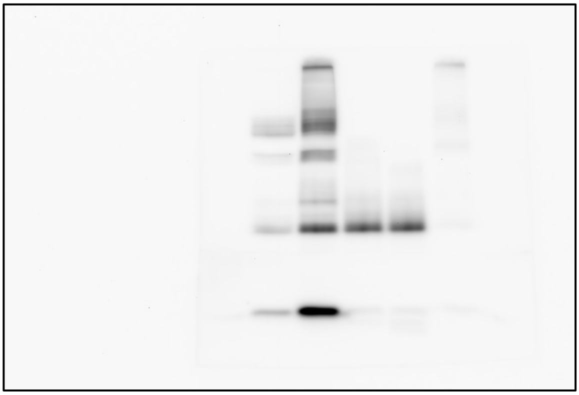

MERGED IMAGES OF MARKER AND PROTEIN

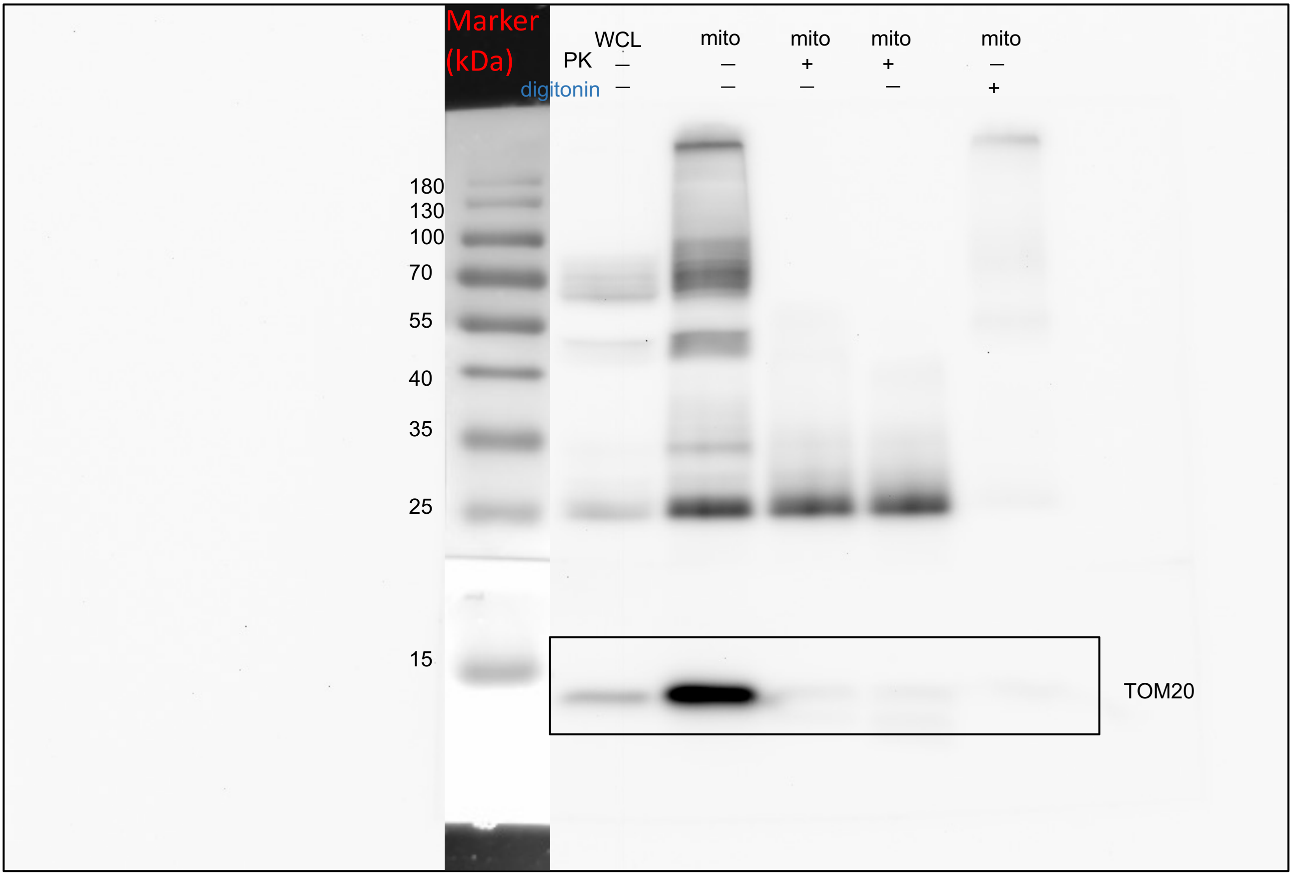

Fig 2P (TIM50)

RAW IMAGES OF MARKER AND PROTEIN BLOTS

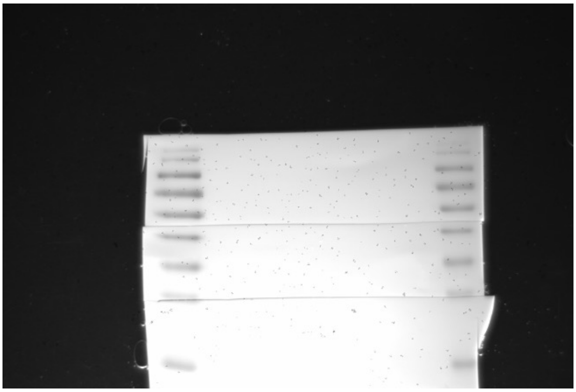

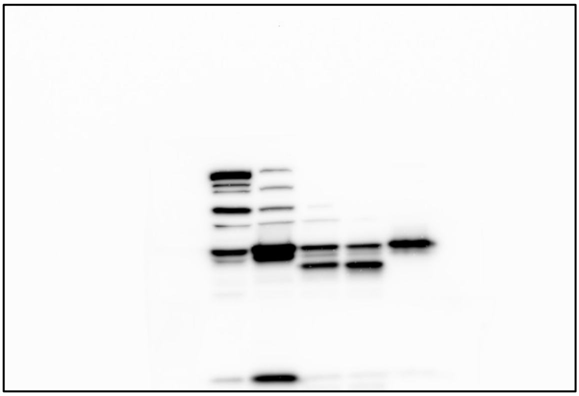

MERGED IMAGES OF MARKER AND PROTEIN

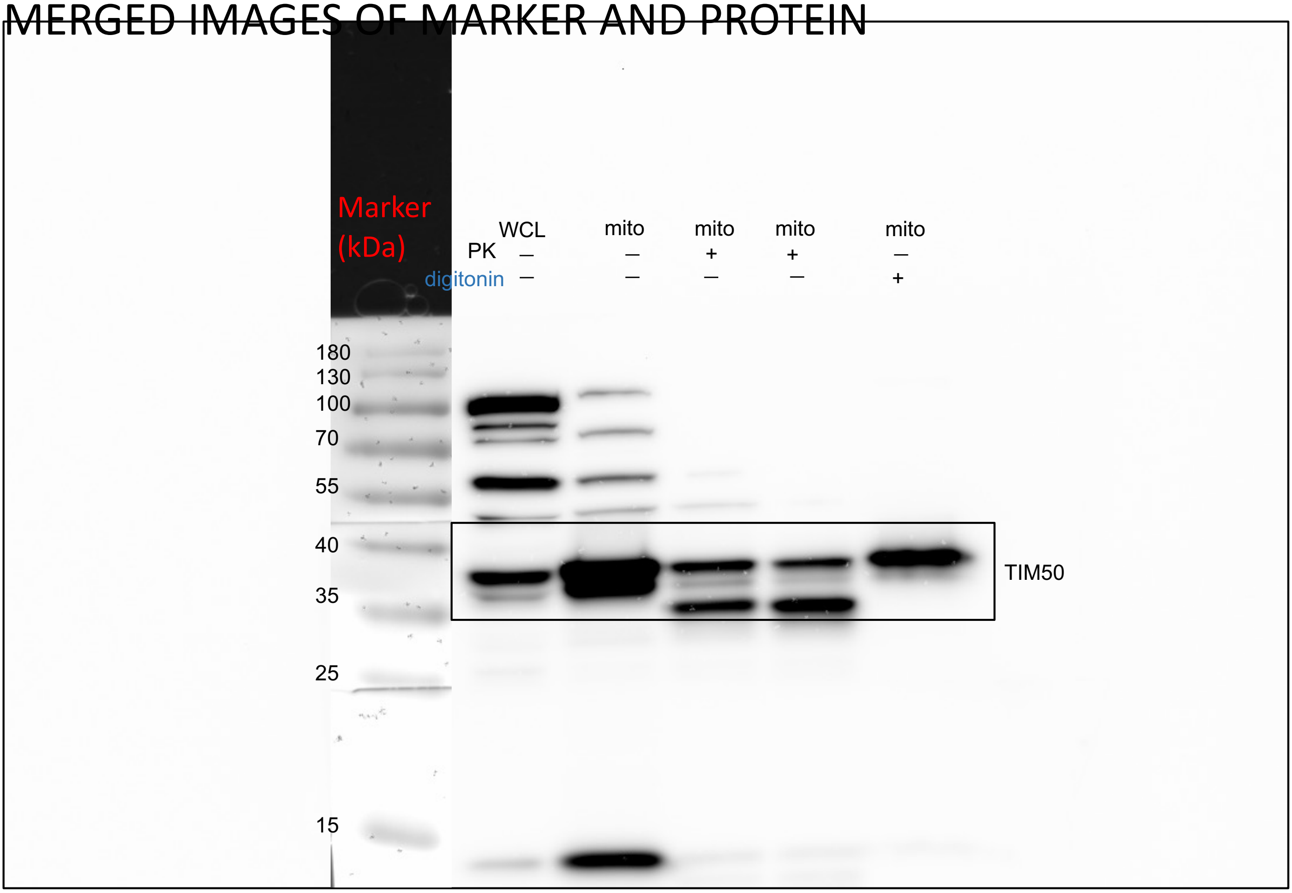

Fig 2P (GLUT10/V5, TIM 50 and TOM 20) Replicate

RAW IMAGES OF PROTEIN BLOTS with marker

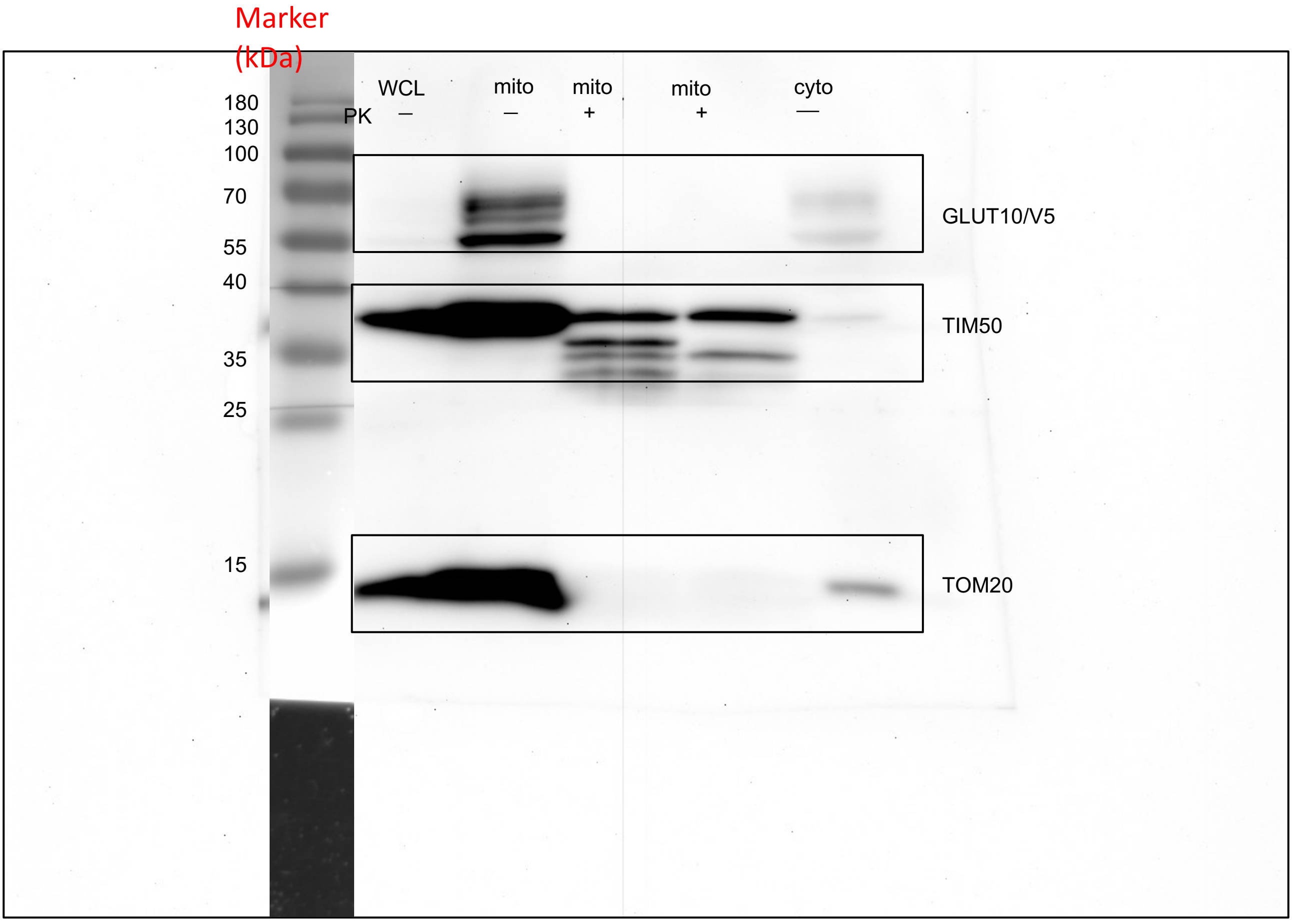

Fig 2P (Calnexin)

RAW IMAGES OF MARKER AND PROTEIN BLOTS

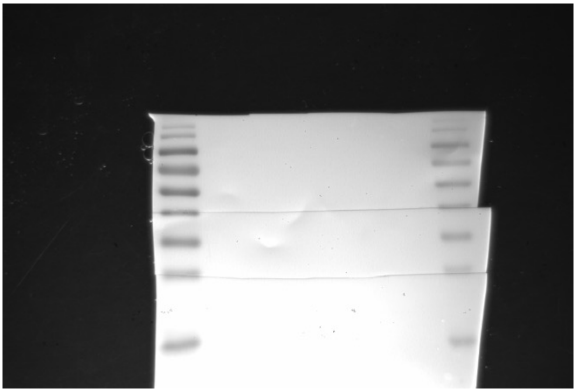

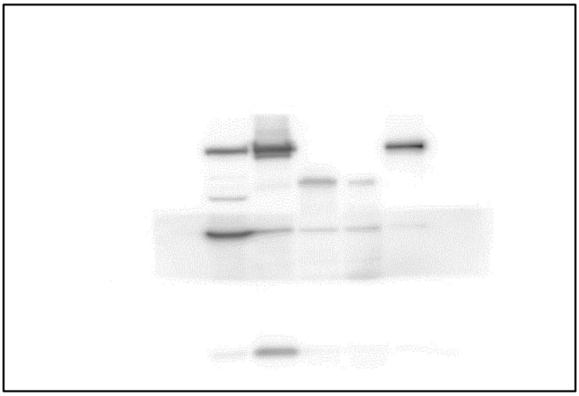

MERGED IMAGES OF MARKER AND PROTEIN

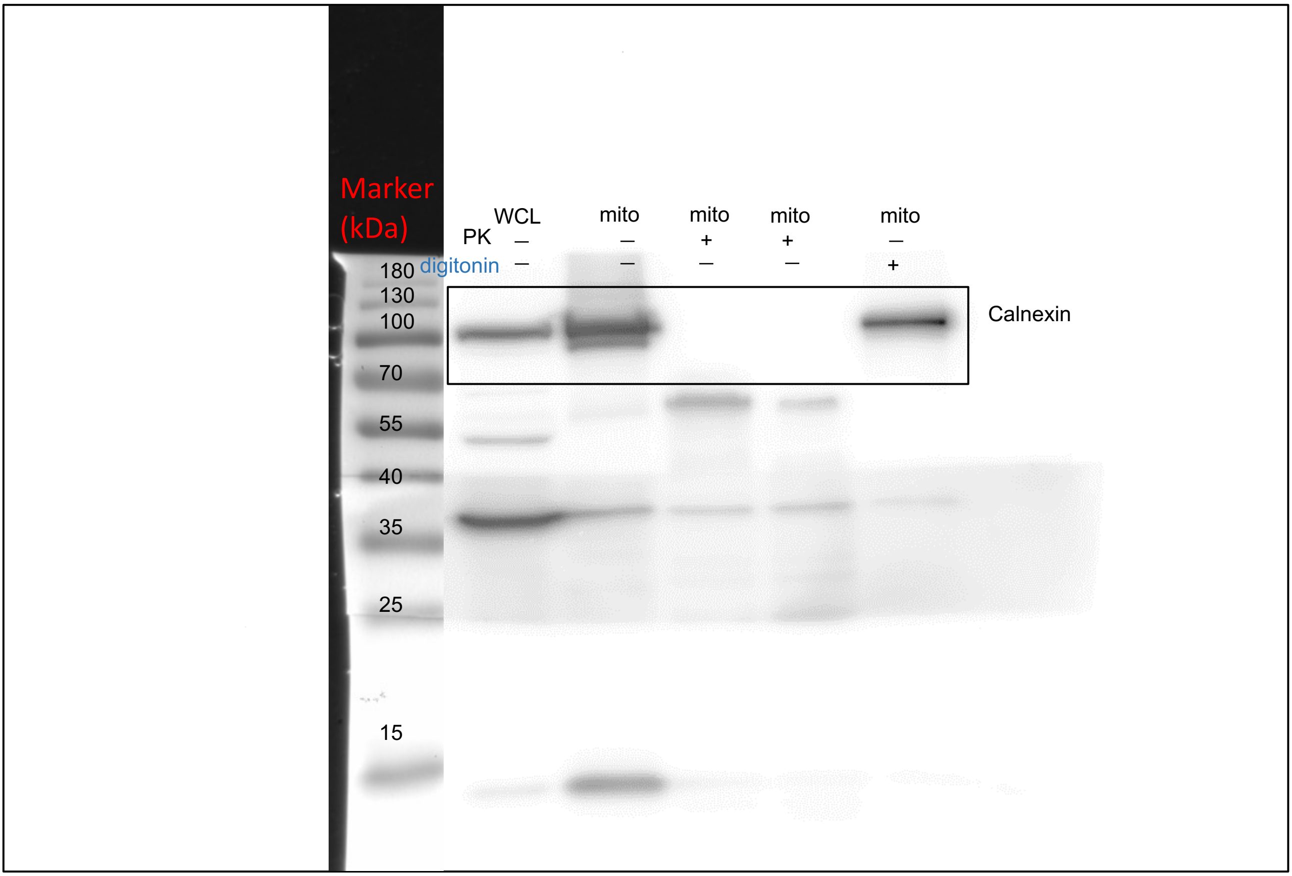

Figure 4A (GLUT10/GFP and HSP60)

RAW AND REPLICATED MERGED IMAGES OF MARKER AND PROTEIN BLOTS

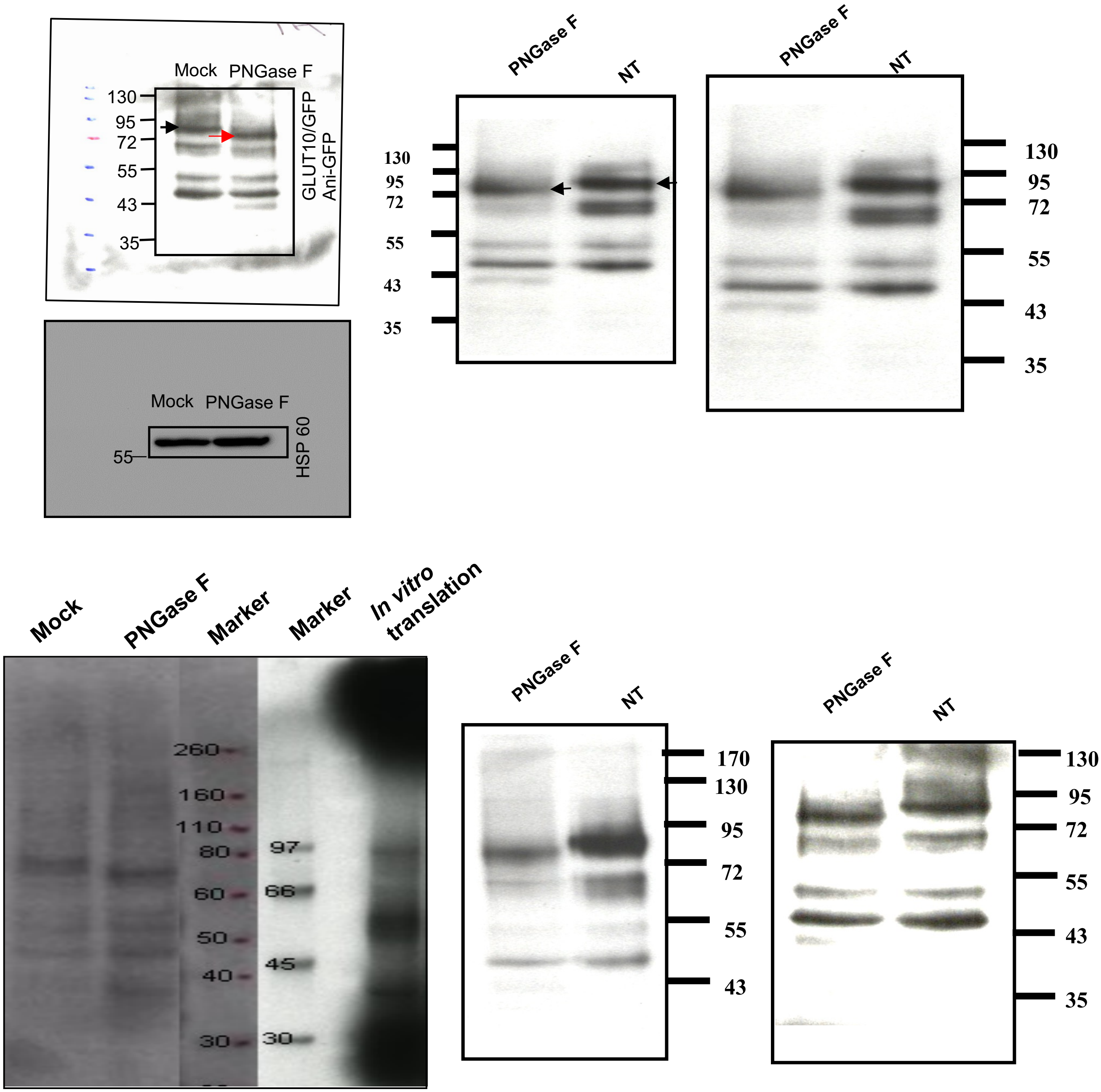

Figure 4C (GLUT10/V5 and TIM50)

RAW IMAGES OF MARKER AND PROTEIN BLOTS

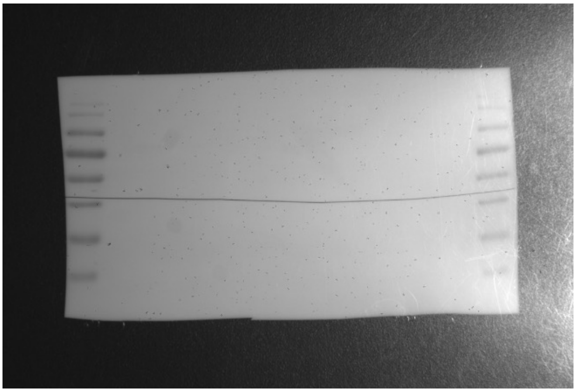

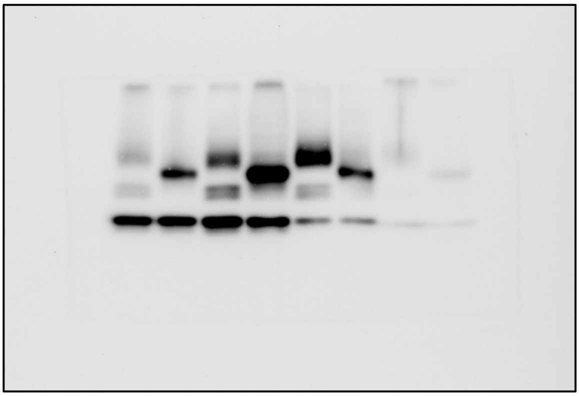

MERGED IMAGES OF MARKER AND PROTEIN

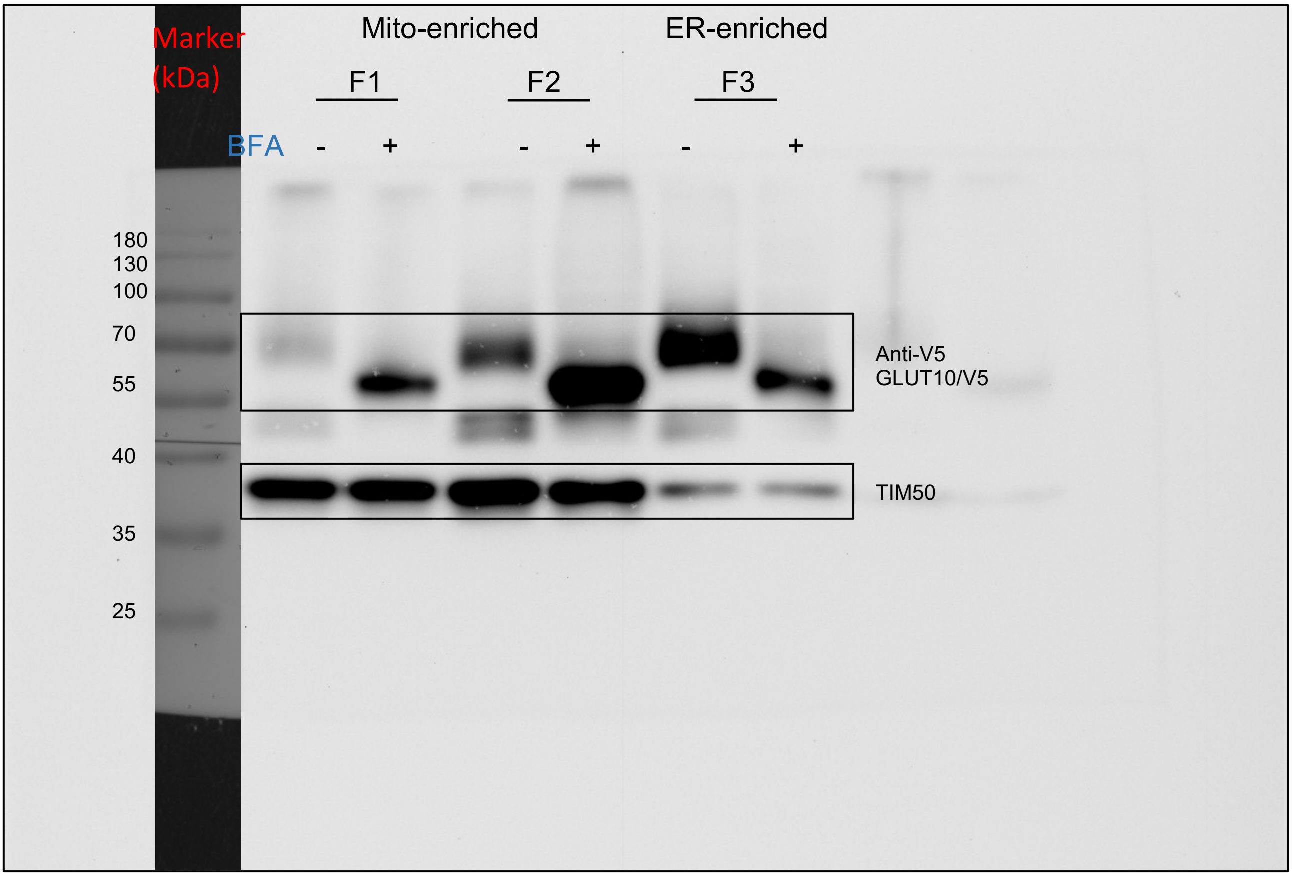

Figure 4C (GLUT10/V5 and TIM50) (replicate)

RAW IMAGES OF PROTEIN BLOTS with marker

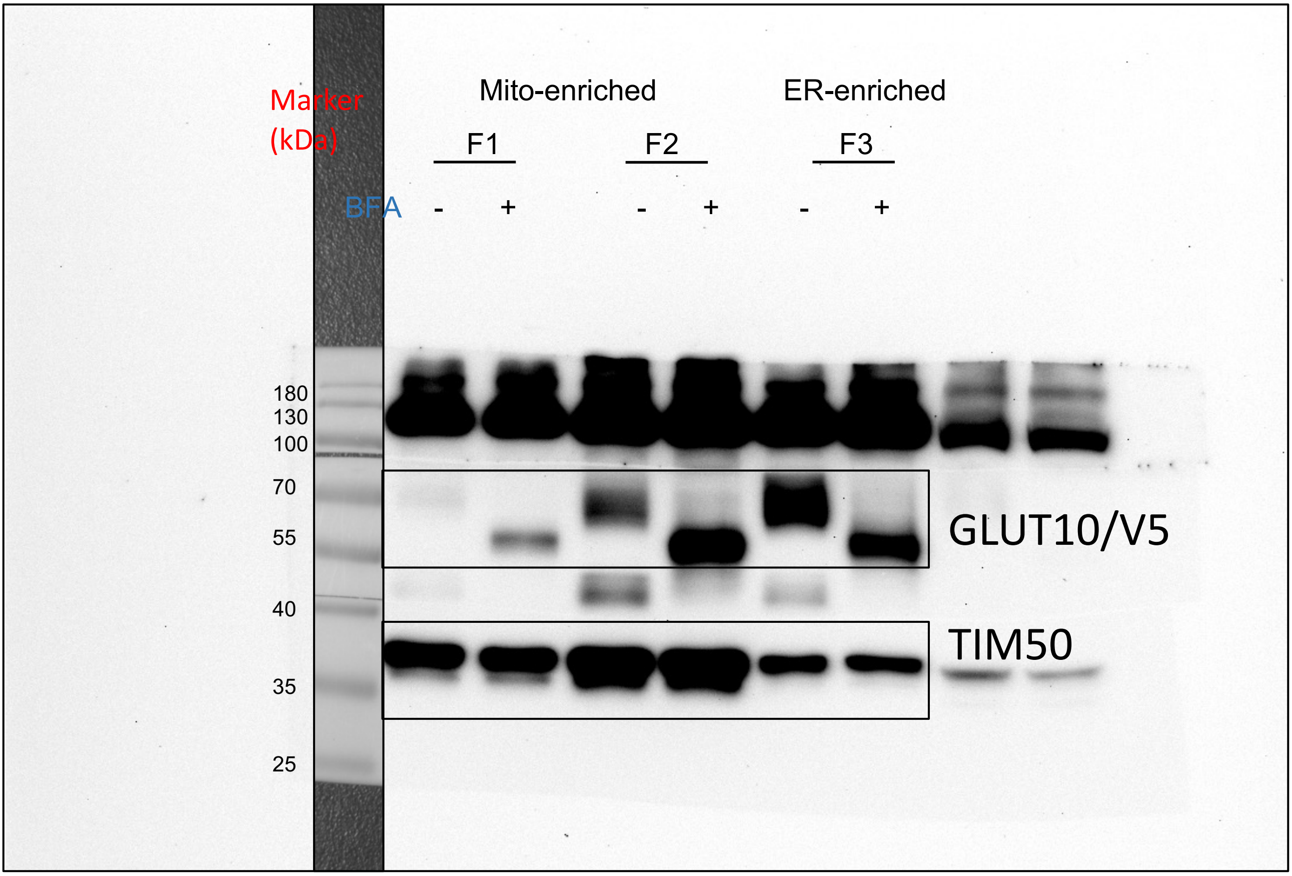

Figure 4C (CALRETICULIN, CRT)

RAW IMAGES OF MARKER AND PROTEIN BLOTS

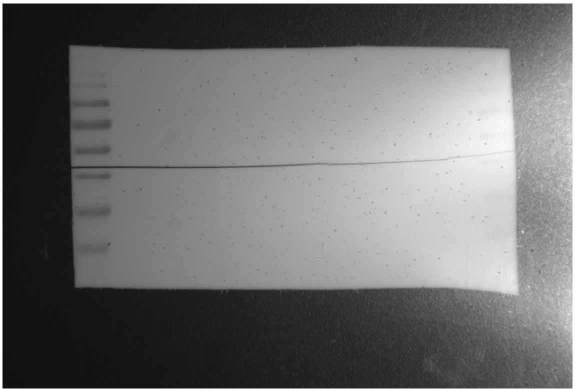

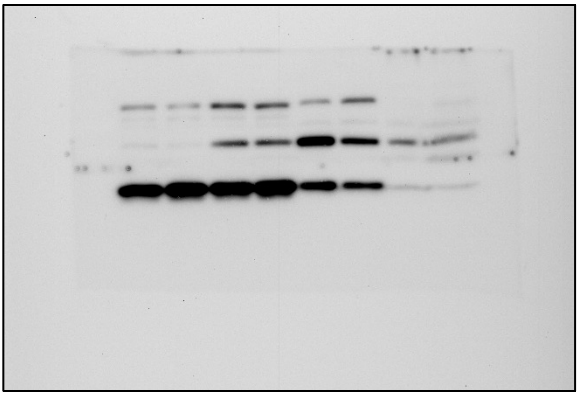

MERGED IMAGES OF MARKER AND PROTEIN

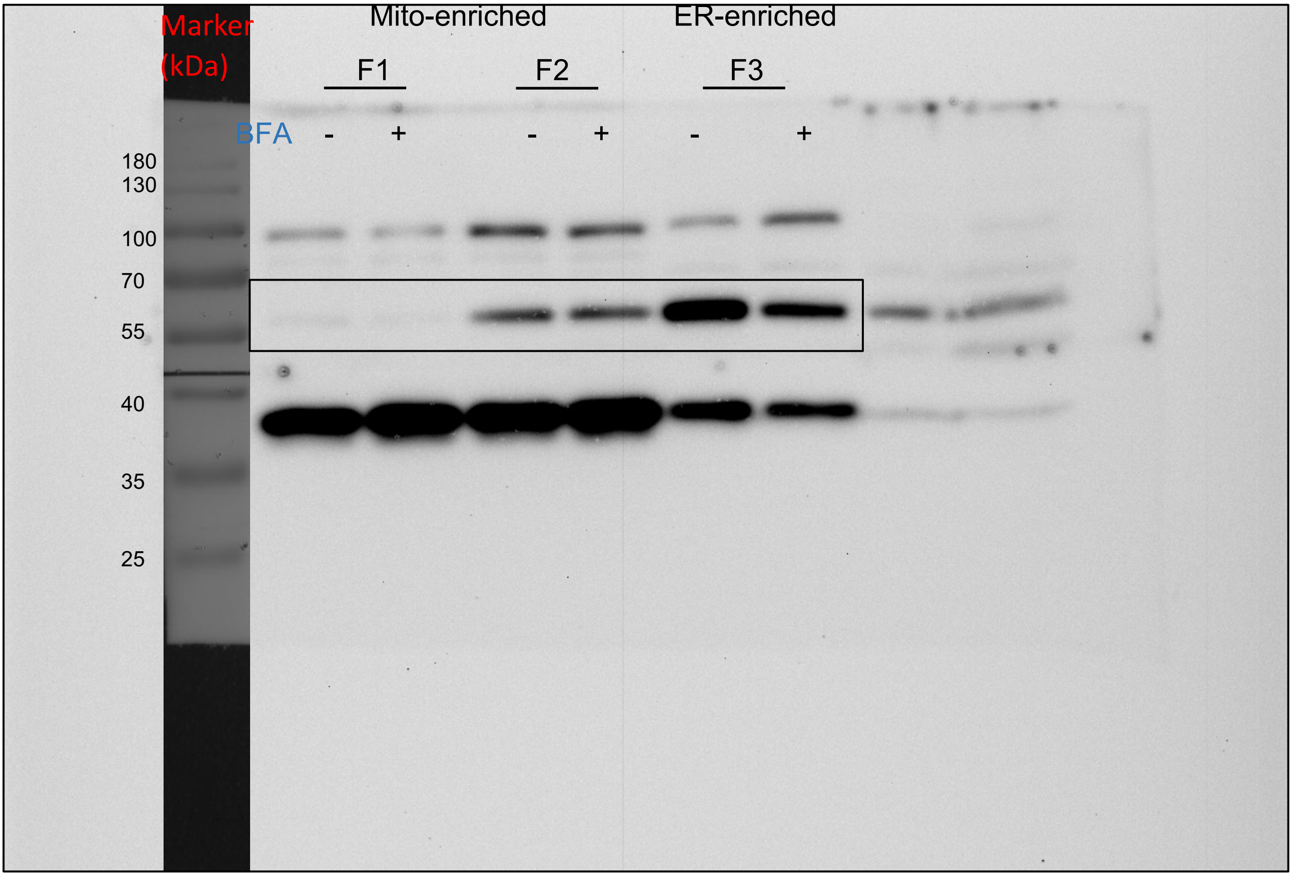

Figure 4C (CALRETICULIN, CRT), replicate

RAW IMAGES OF PROTEIN BLOTS with marker

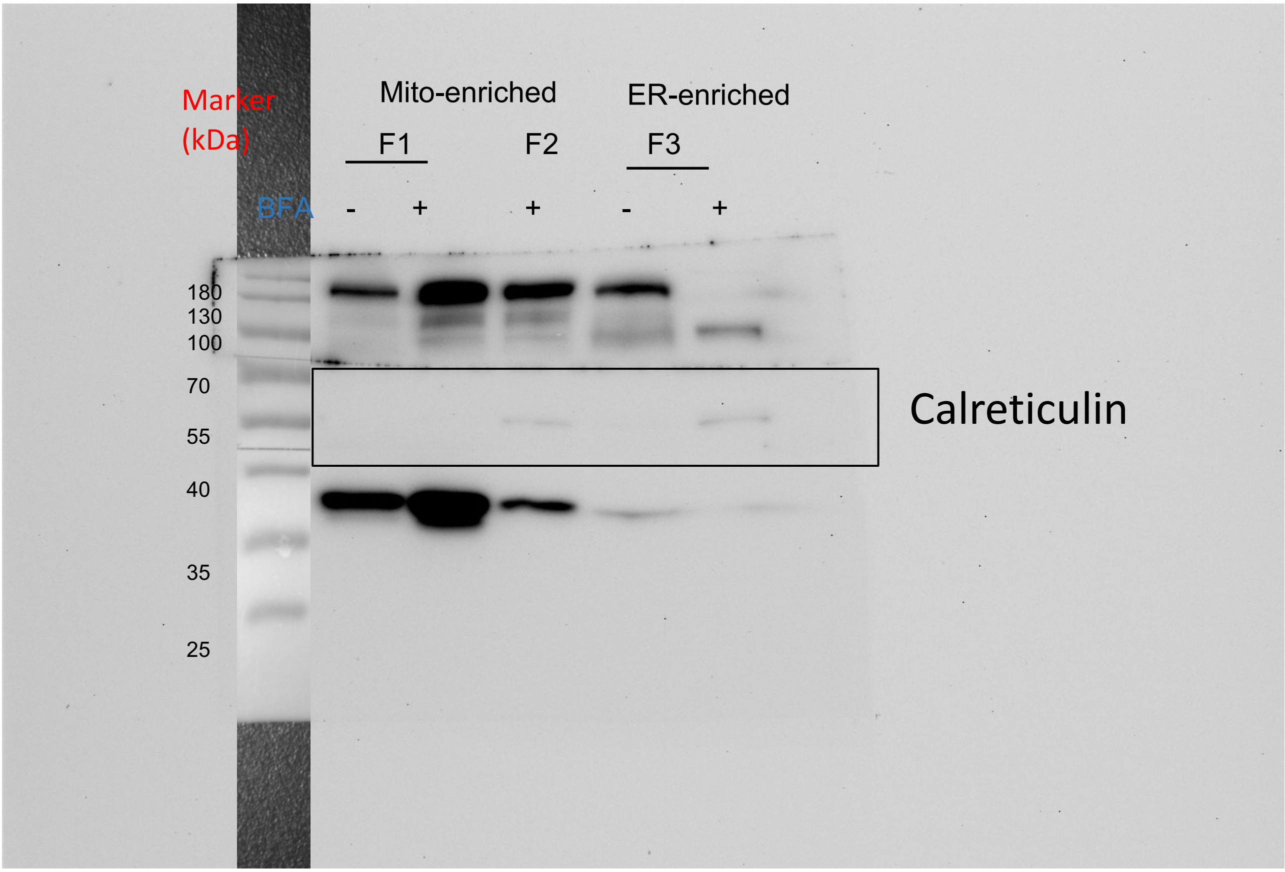

Figure 5M (GLUT10/V5)

RAW IMAGES OF MARKER AND PROTEIN BLOTS

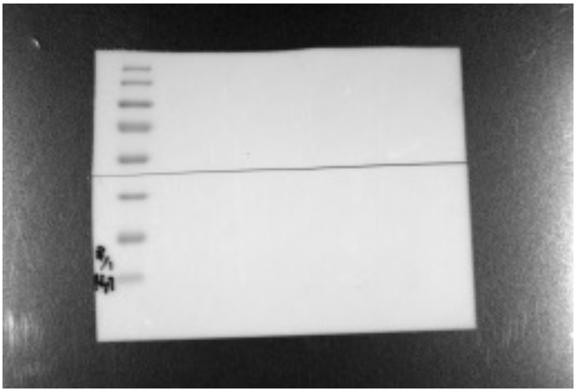

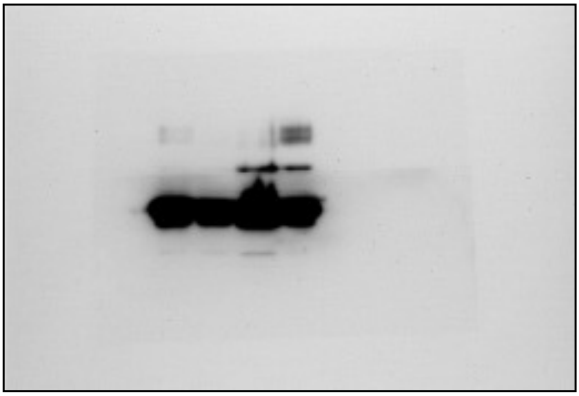

MERGED IMAGES OF MARKER AND PROTEIN

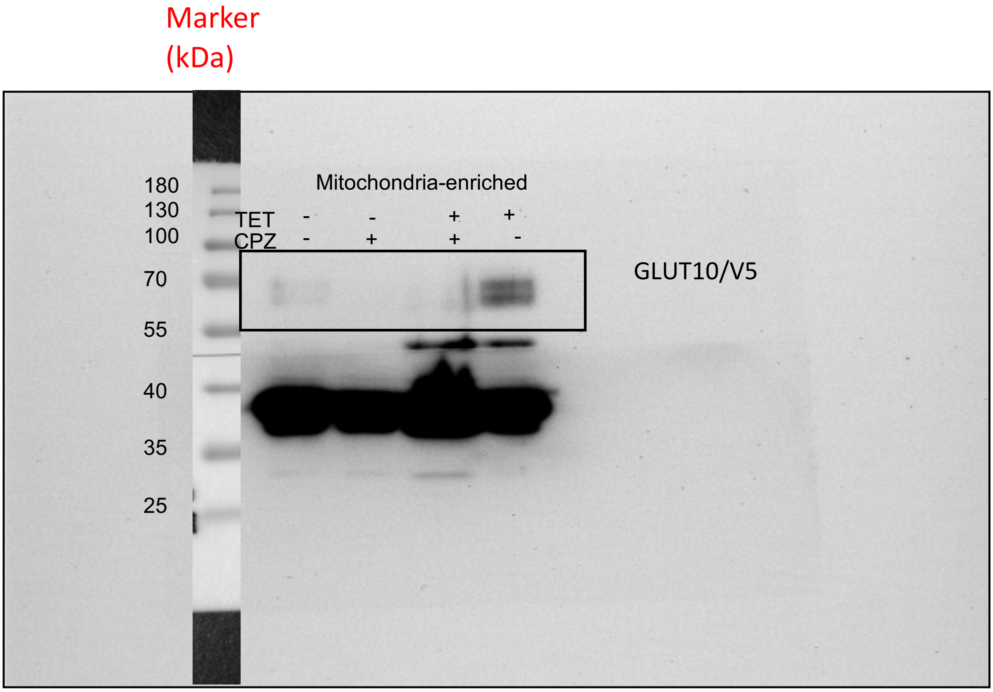

Figure 5M (TIM 50)

RAW IMAGES OF MARKER AND PROTEIN BLOTS

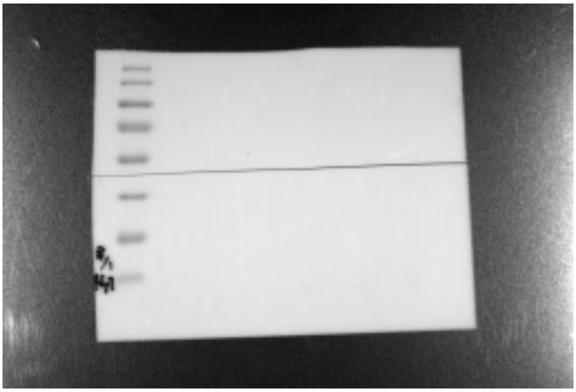

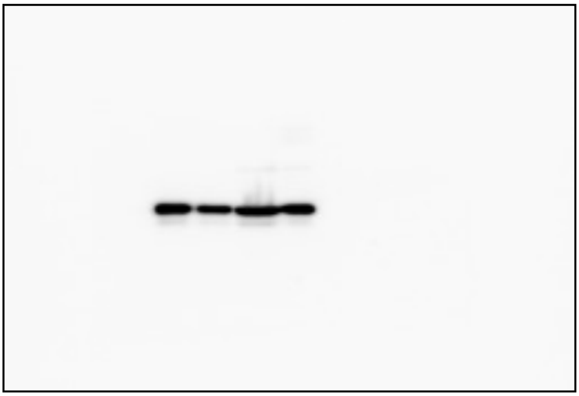

MERGED IMAGES OF MARKER AND PROTEIN

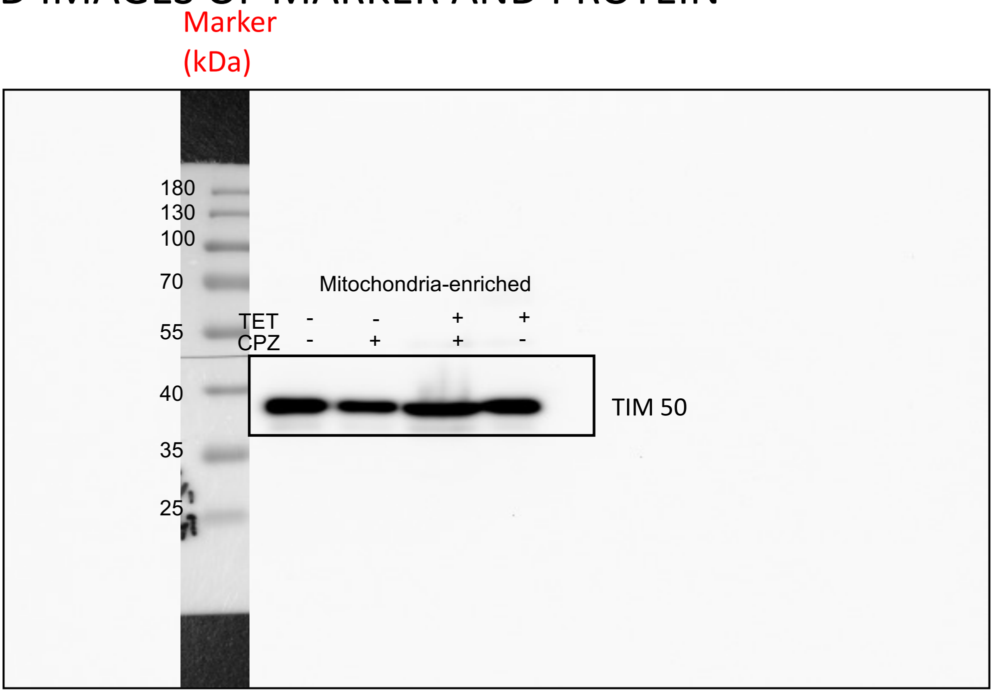

Figure 5M (GLUT10/V5, TIM 50 replicate)

RAW IMAGES OF PROTEIN BLOTS WITH MARKED Molecular weight

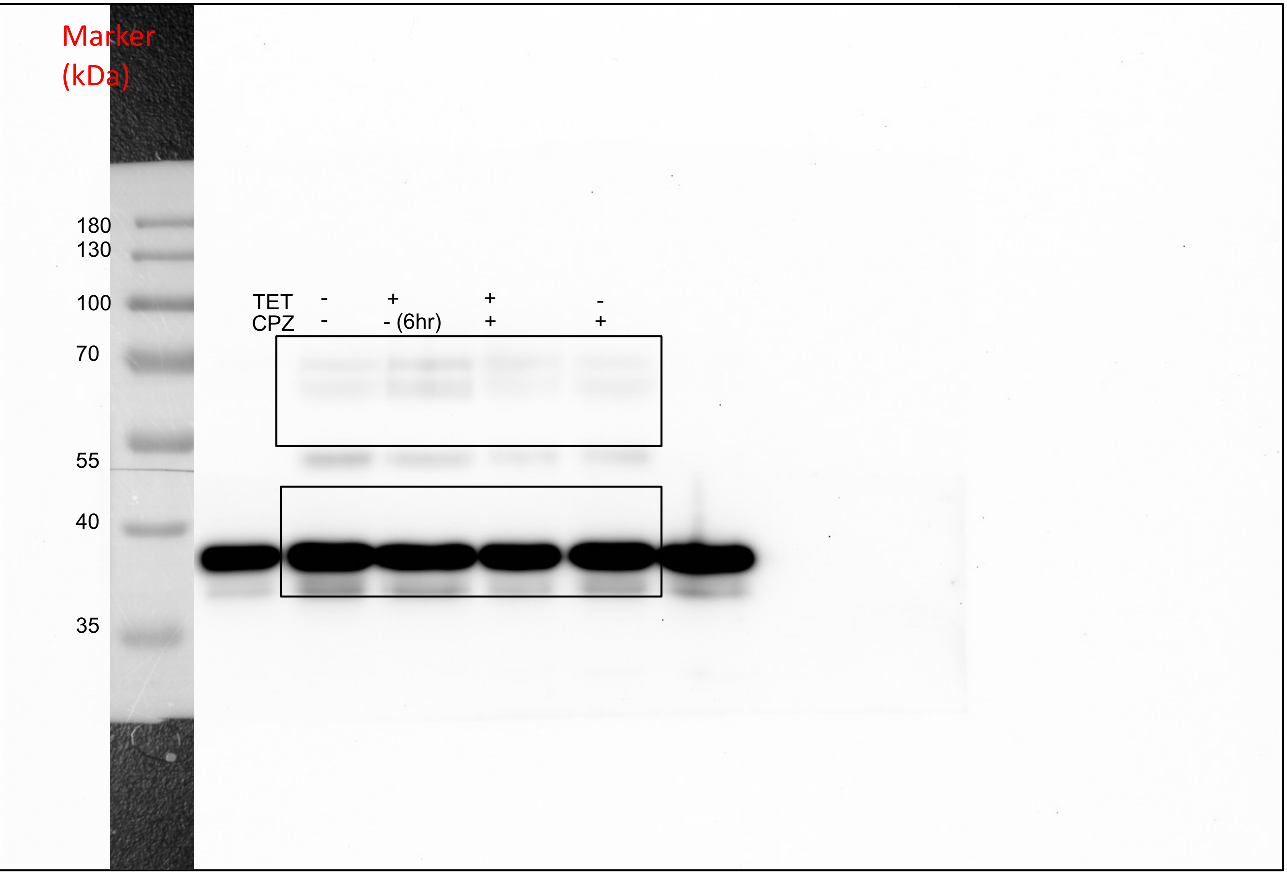

Figure 5N (GLUT10/V5 and beta Actin)

RAW IMAGES OF MARKER AND PROTEIN BLOTS

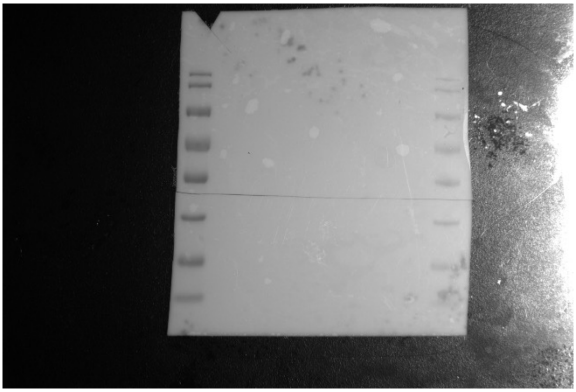

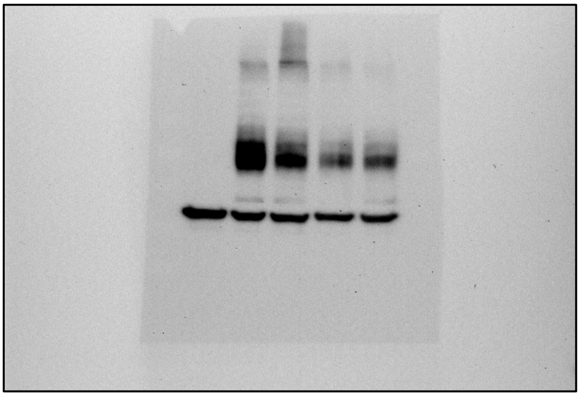

MERGED IMAGES OF MARKER AND PROTEIN

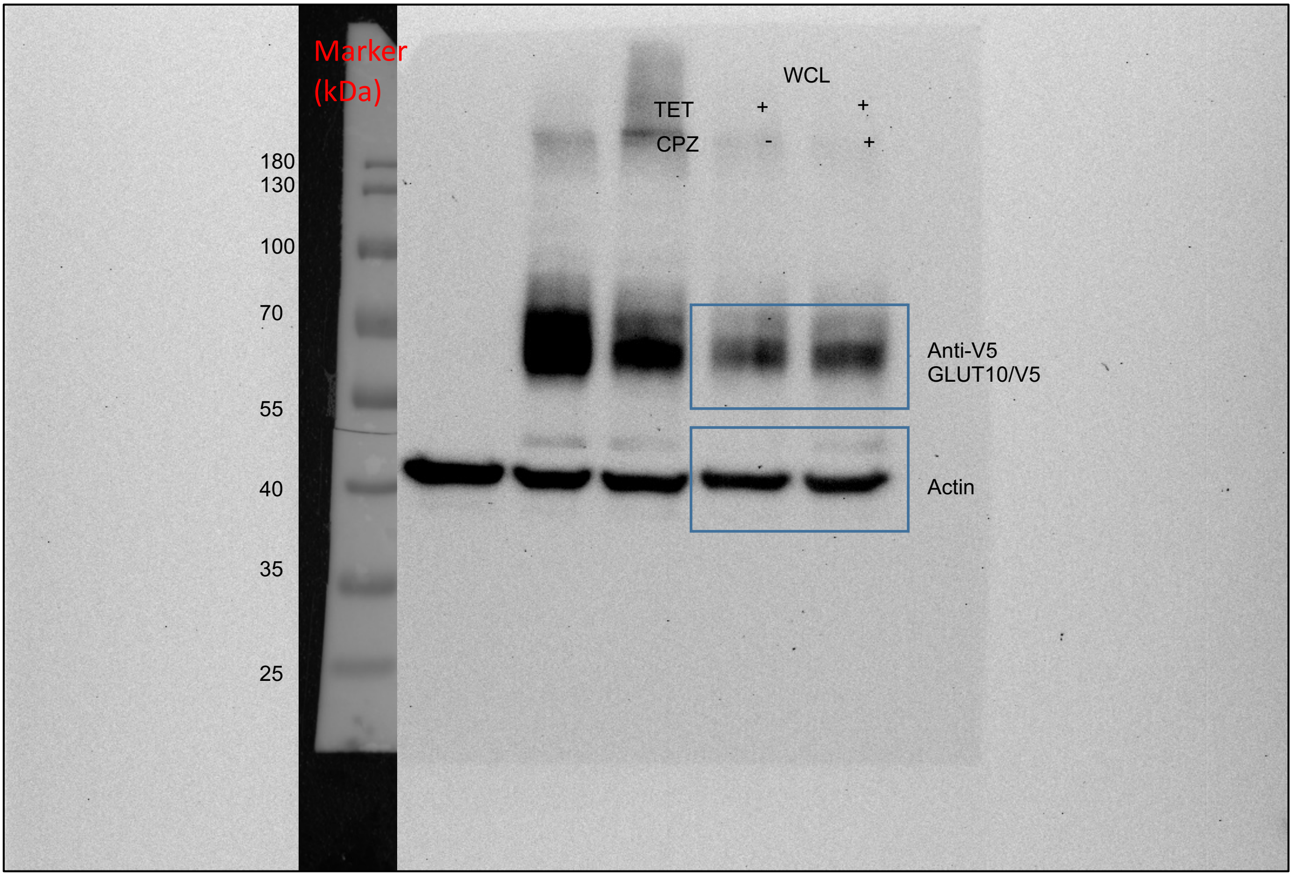

Figure 5N (GLUT10/V5, Beta actin replicate)

RAW IMAGES OF PROTEIN BLOTS WITH MARKED Molecular weight

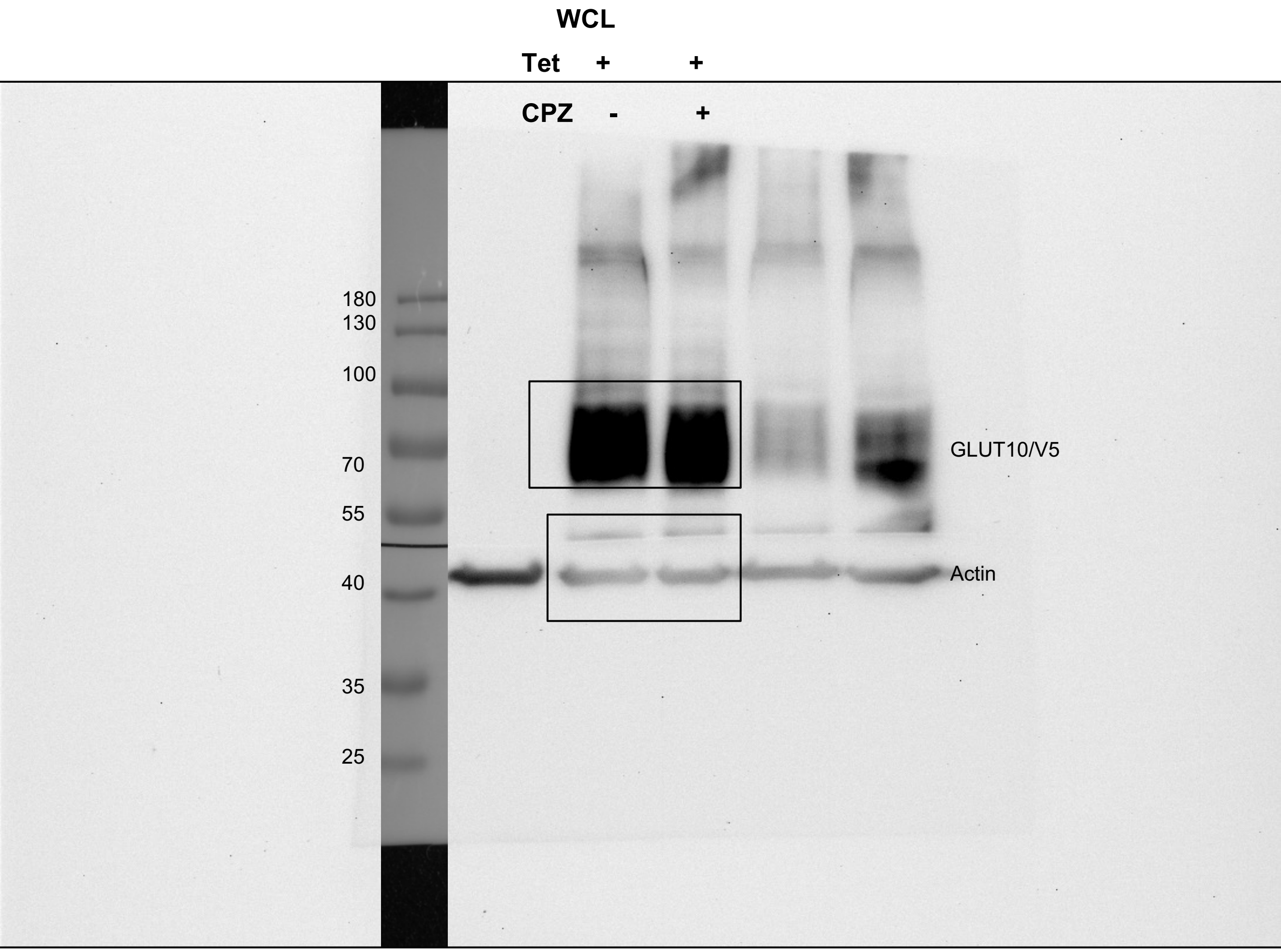

Figure 5O (GLUT10/V5 and TIM 50)

RAW IMAGES OF MARKER AND PROTEIN BLOTS

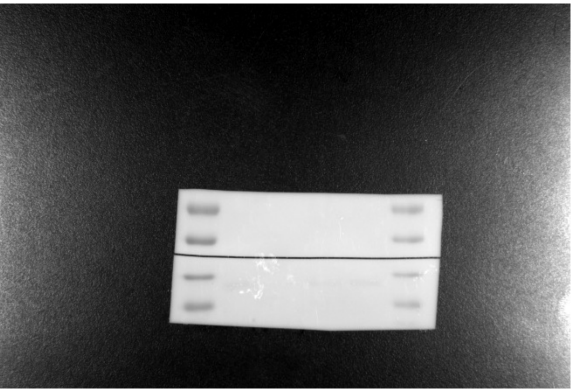

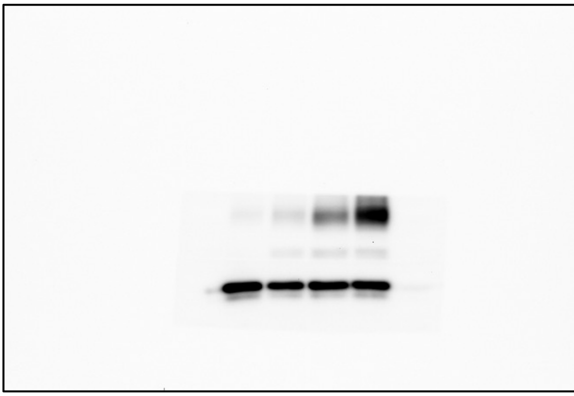

MERGED IMAGES OF MARKER AND PROTEIN

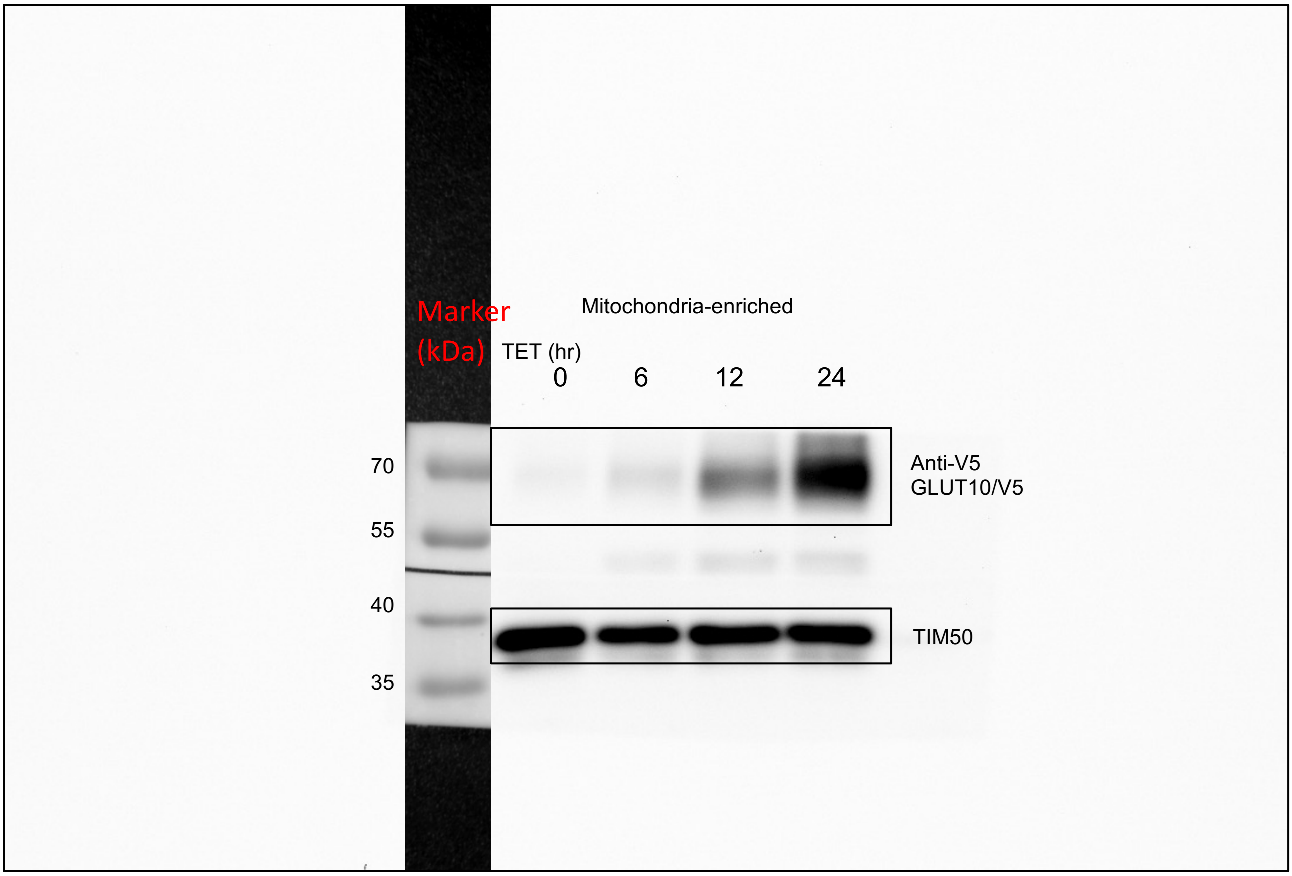

Fig. 6A (GLUT10/V5)

RAW IMAGES OF MARKER AND PROTEIN BLOTS

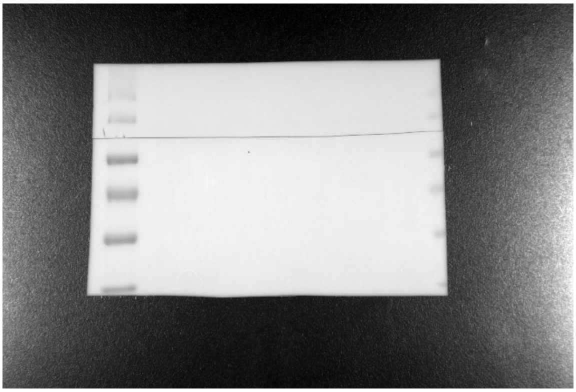

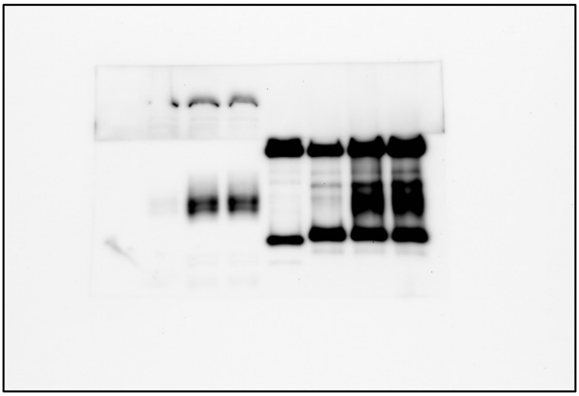

MERGED IMAGES OF MARKER AND PROTEIN

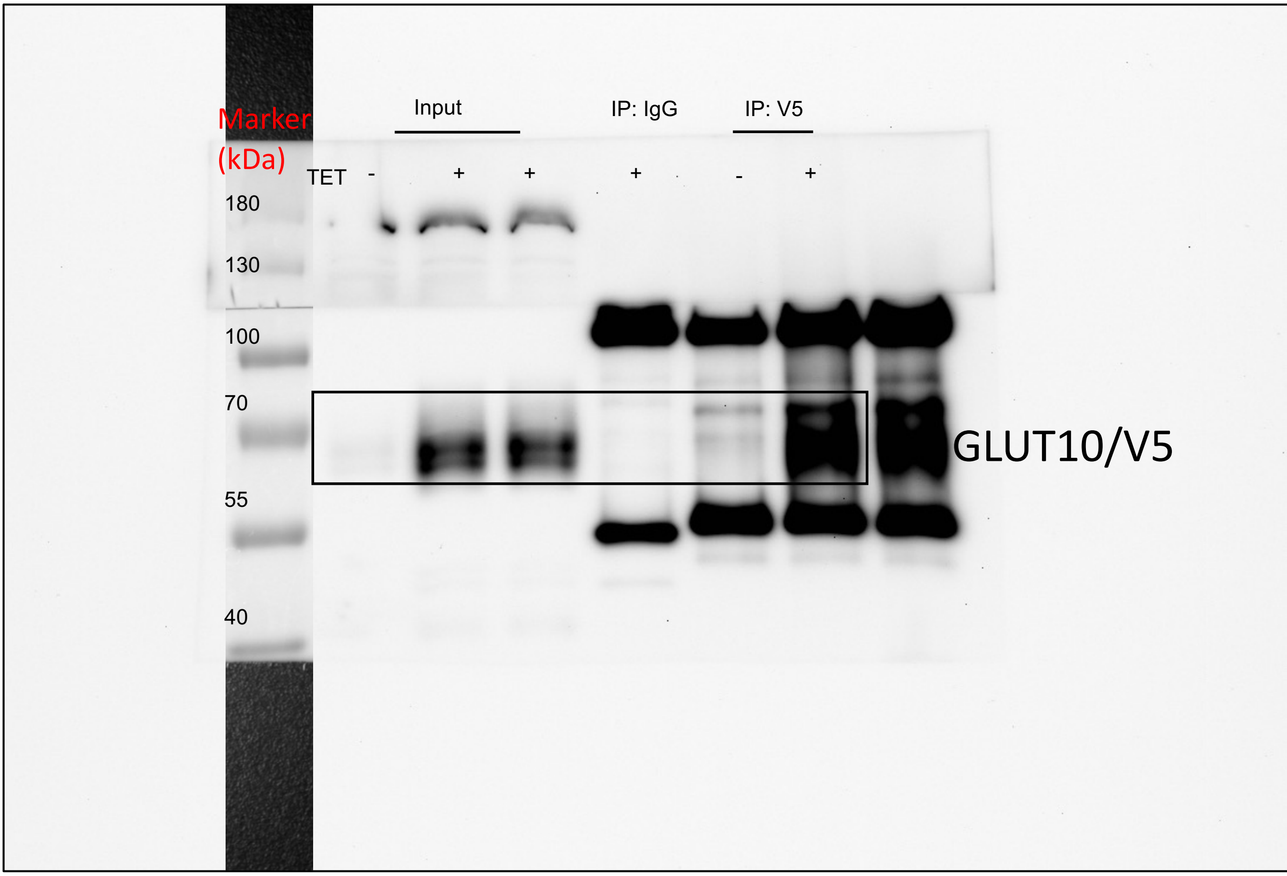

Fig. 6A (GLUT10/V5) (replicate)

RAW IMAGES OF PROTEIN BLOTS WITH MARKED Molecular weight

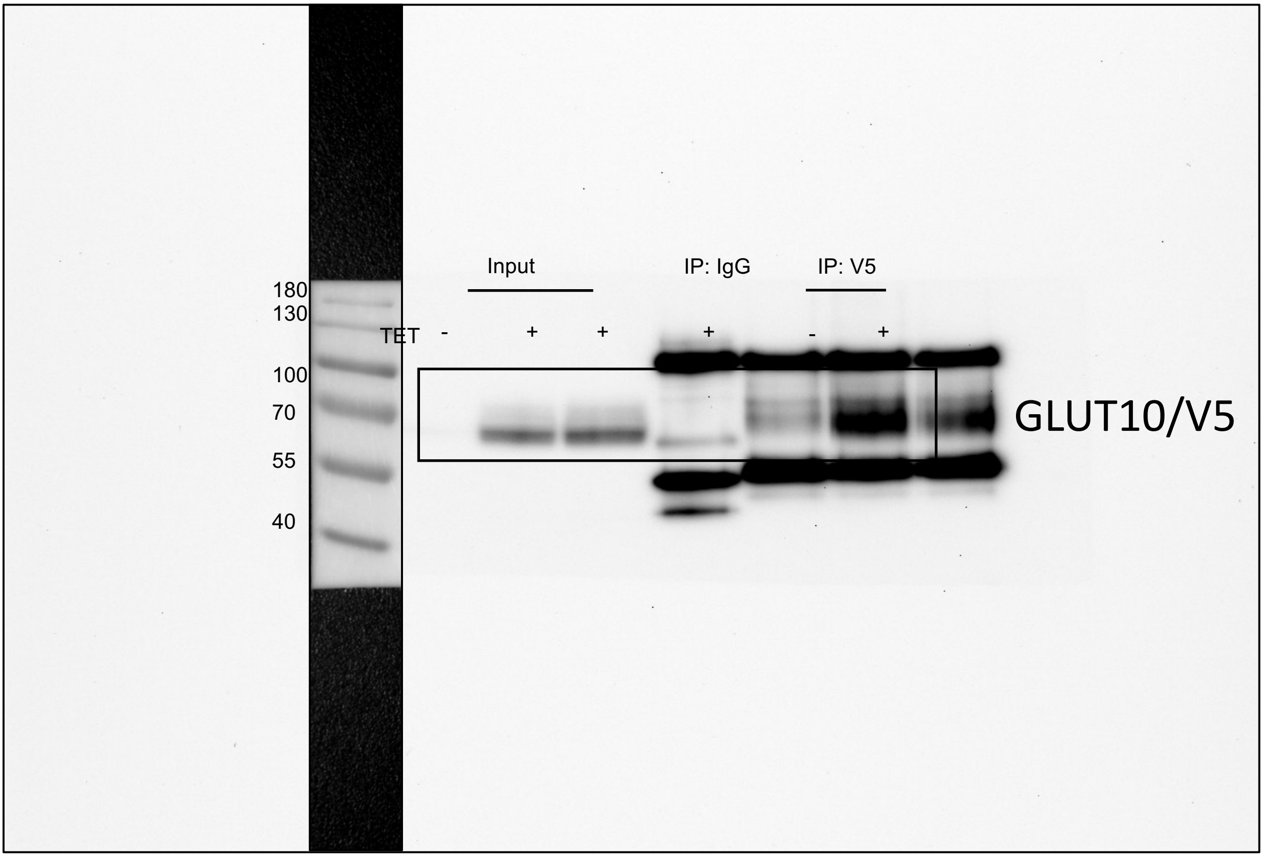

Fig. 6A (RAB5)

RAW IMAGES OF MARKER AND PROTEIN BLOTS

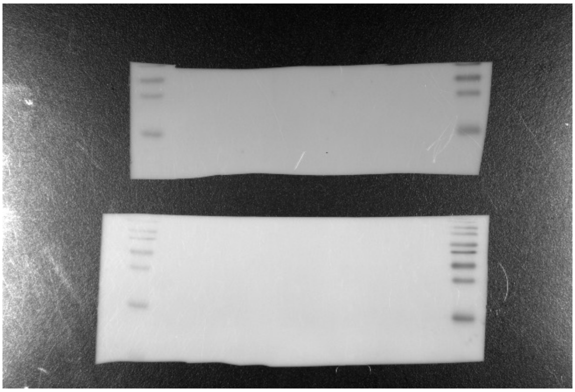

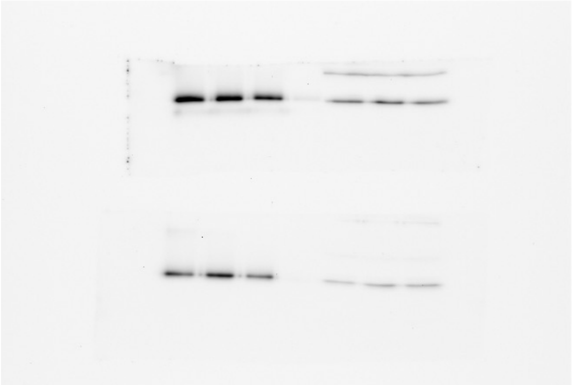

MERGED IMAGES OF MARKER AND PROTEIN

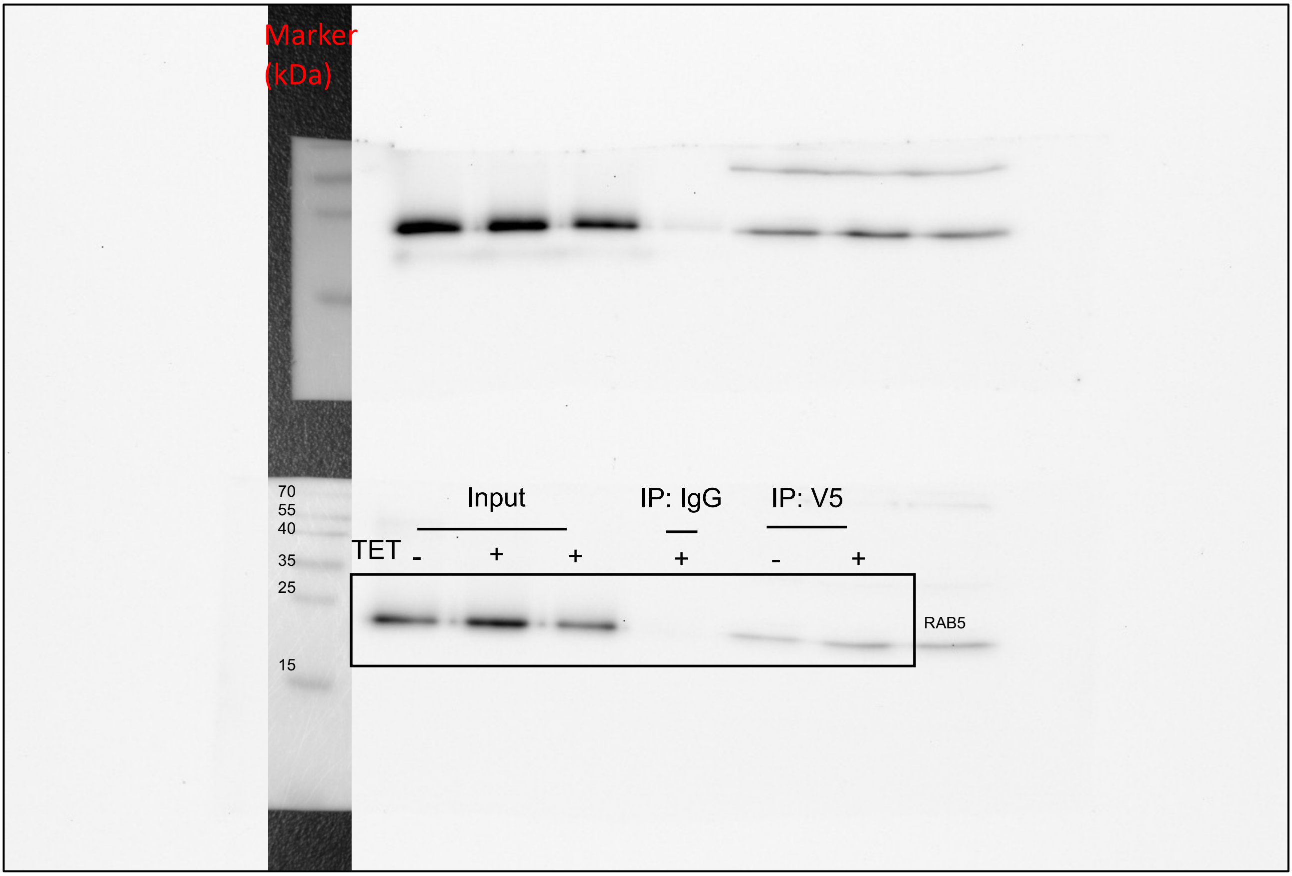

Fig. 6A (RAB5) (Replicate)

RAW IMAGES OF PROTEIN BLOTS WITH MARKED Molecular weight

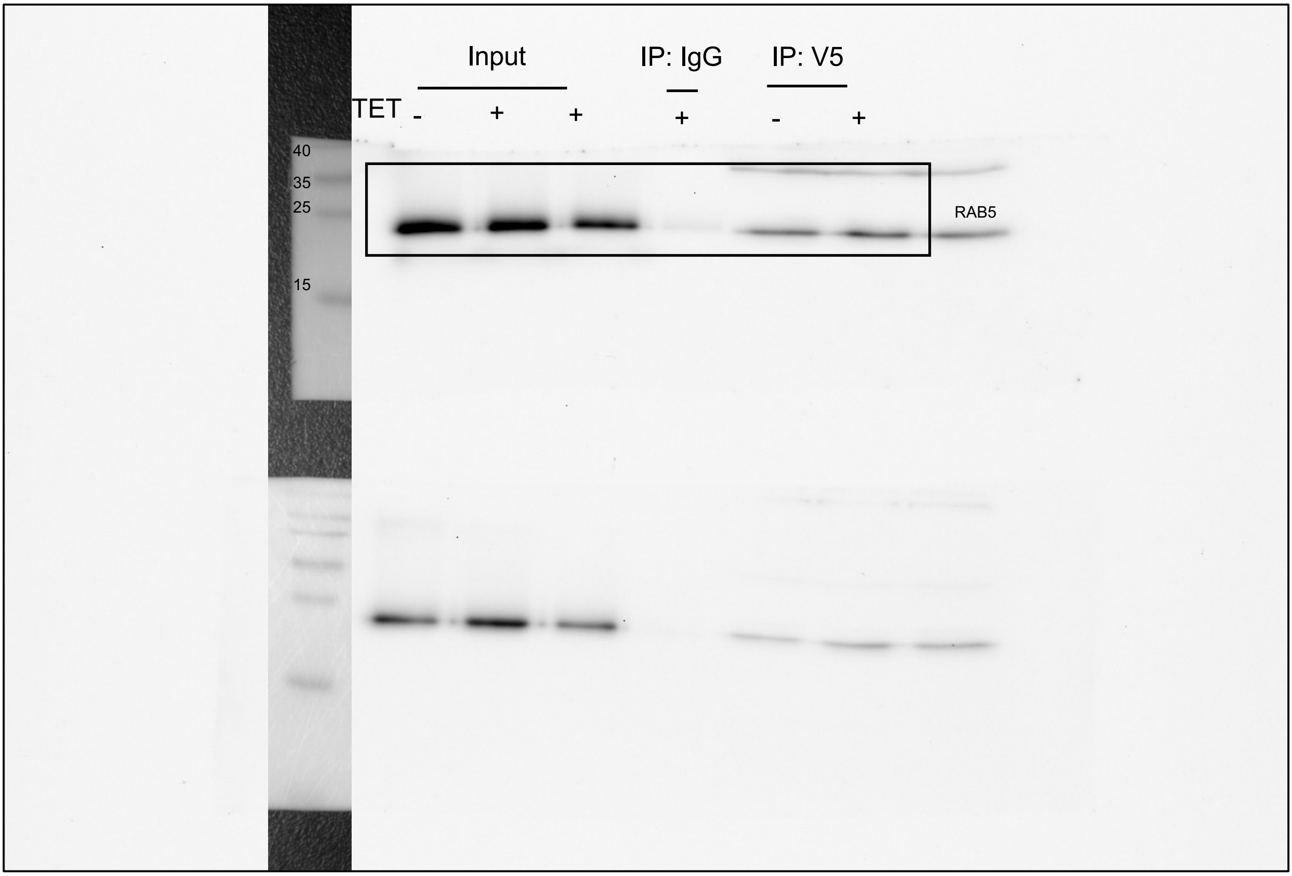

Fig. 6E/RAB5A

RAW IMAGES OF MARKER AND PROTEIN BLOTS

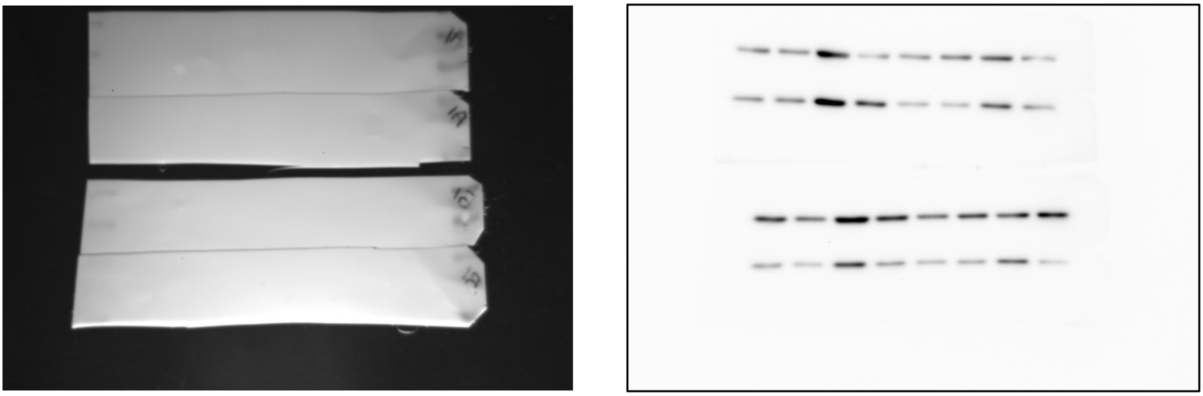

MERGED IMAGES OF MARKER AND PROTEIN

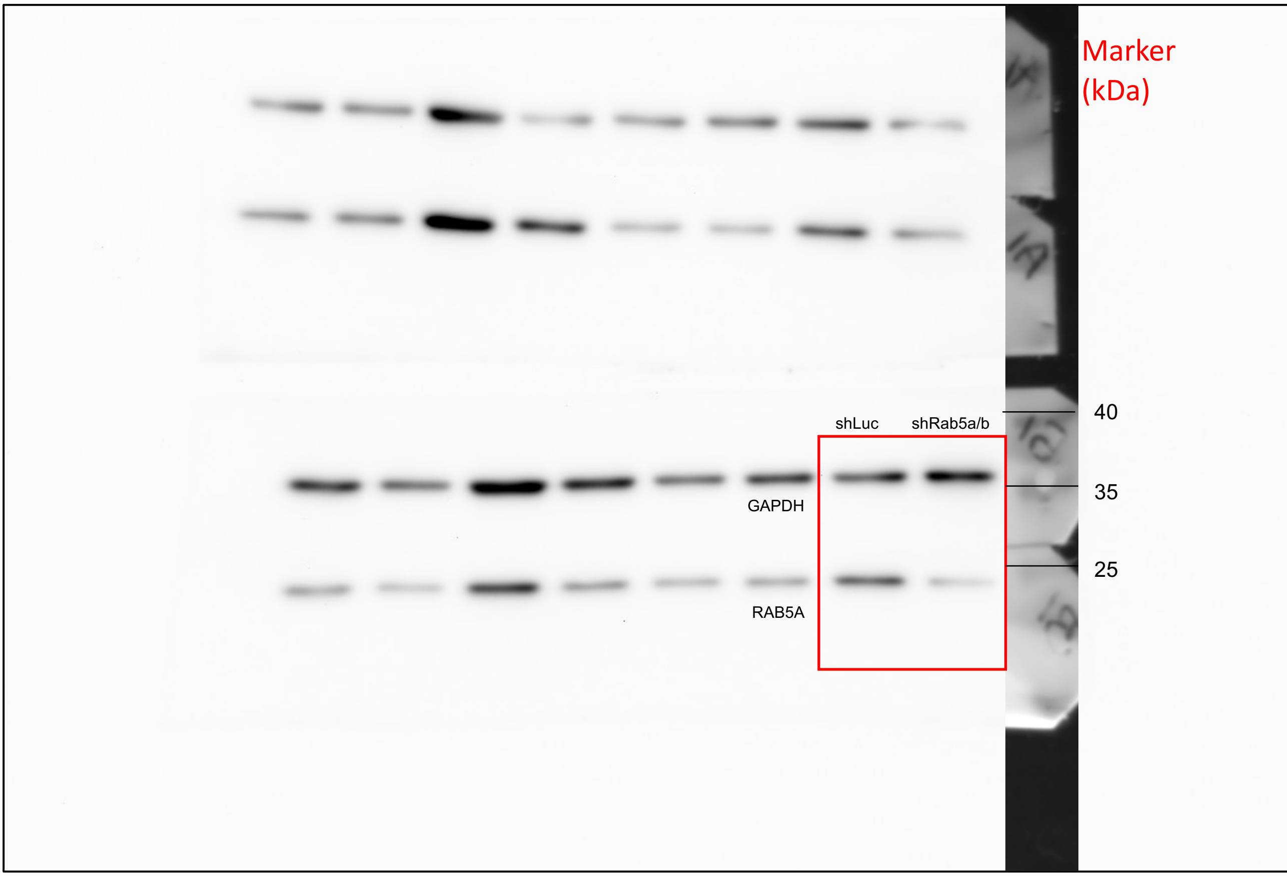

Fig. 6E/RAB5B

RAW IMAGES OF MARKER AND PROTEIN BLOTS

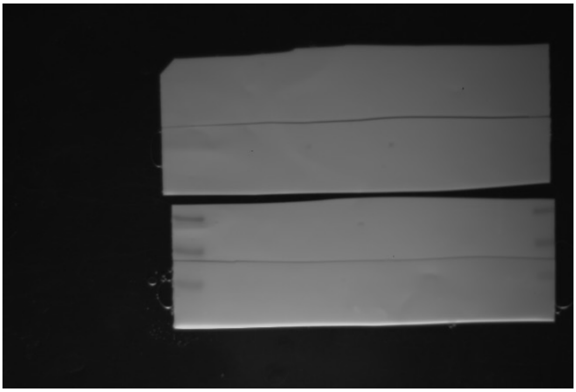

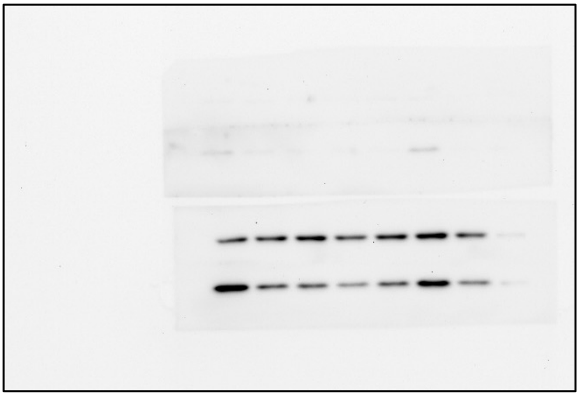

MERGED IMAGES OF MARKER AND PROTEIN

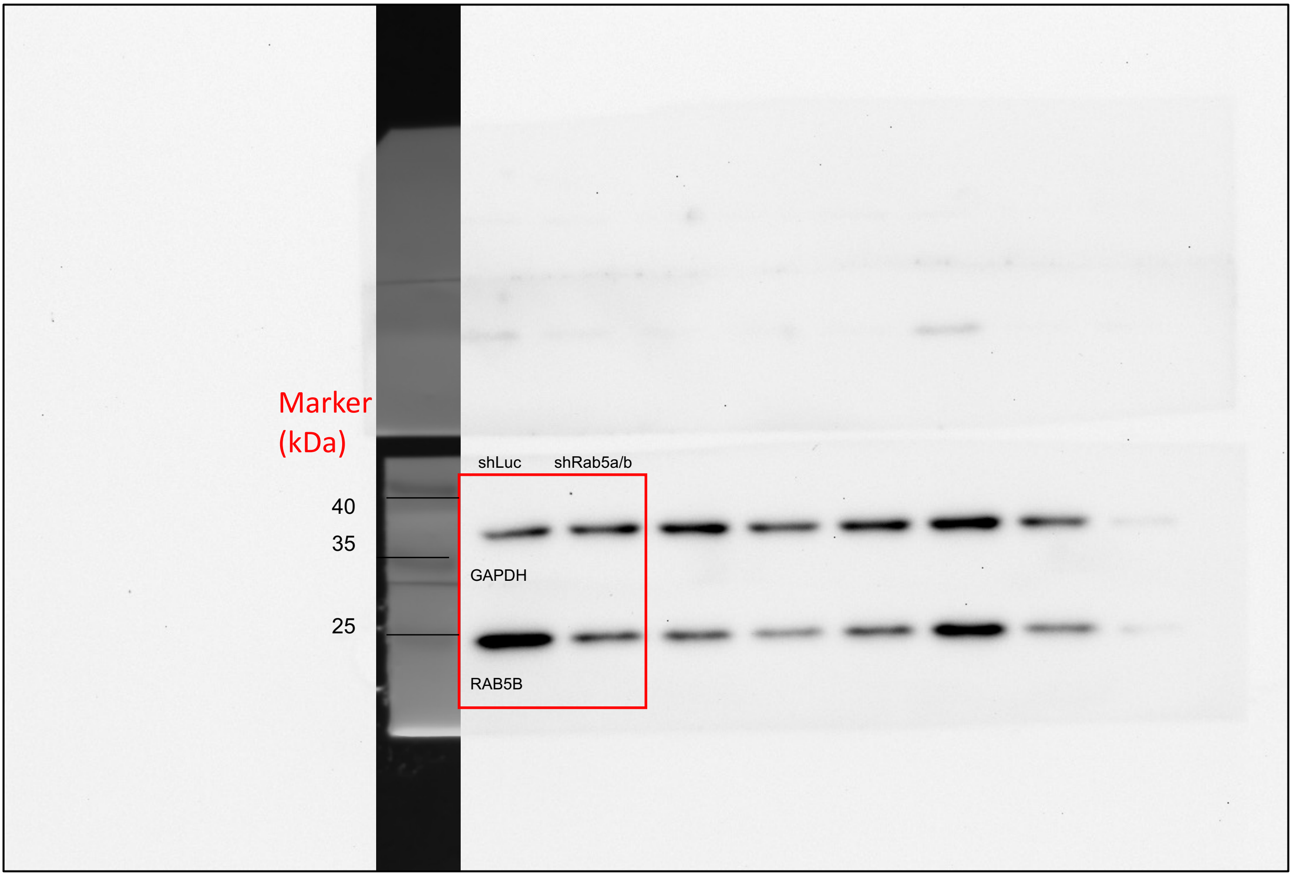

Fig. 7E

RAW IMAGES OF PROTEIN BLOTS WITH MARKED Molecular weight

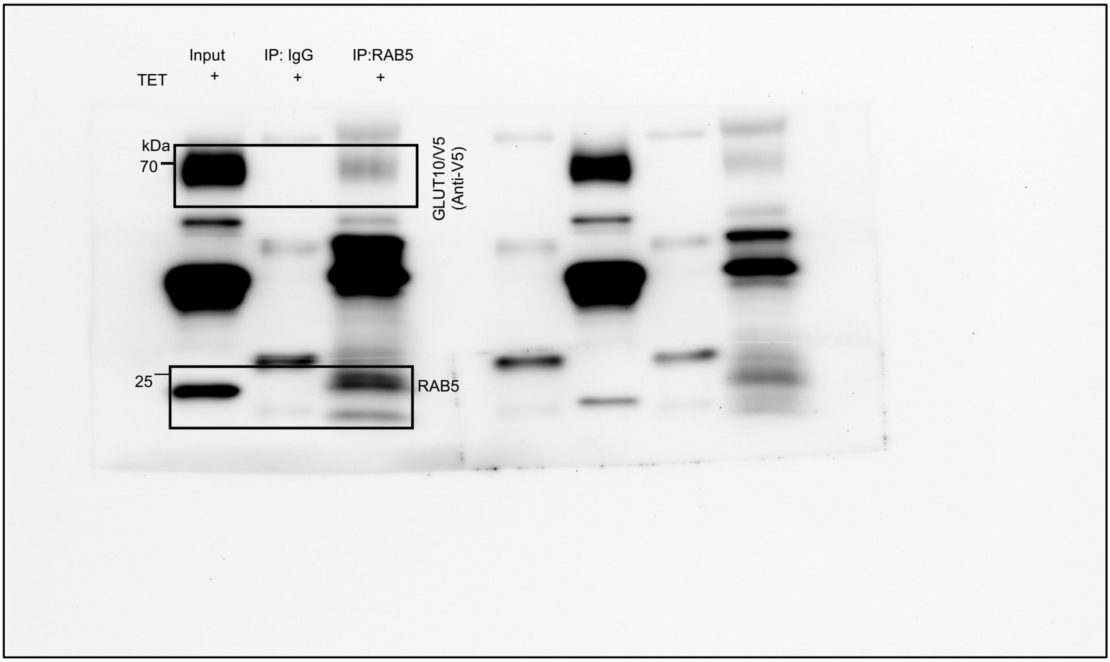

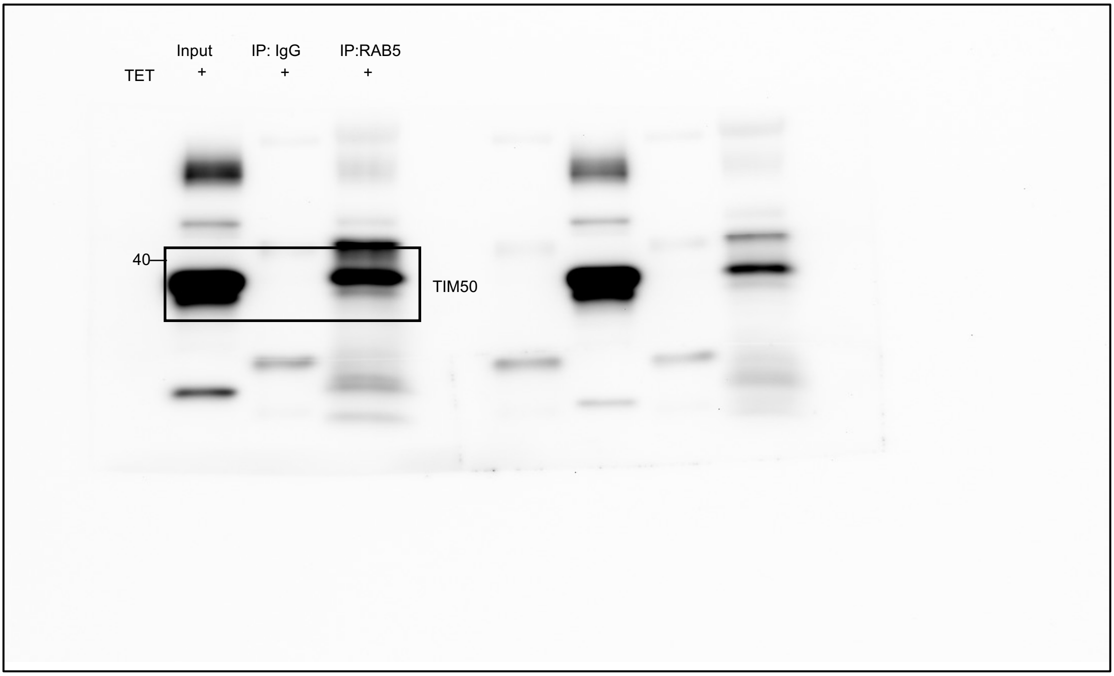

Fig. 7E (Replicate)

RAW IMAGES OF PROTEIN BLOTS WITH MARKED Molecular weight

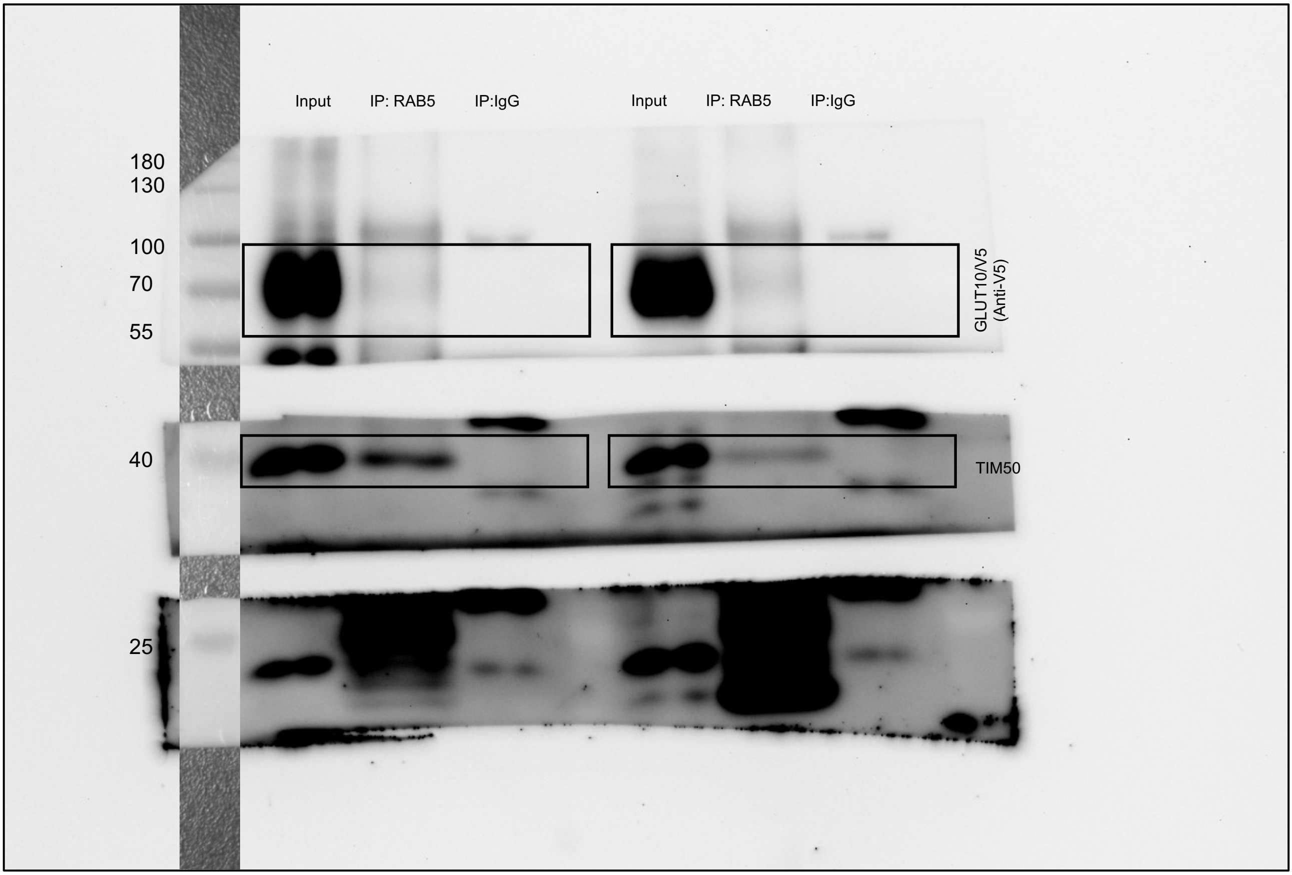

Fig. 7E (Replicate)

RAW IMAGES OF PROTEIN BLOTS WITH MARKED Molecular weight

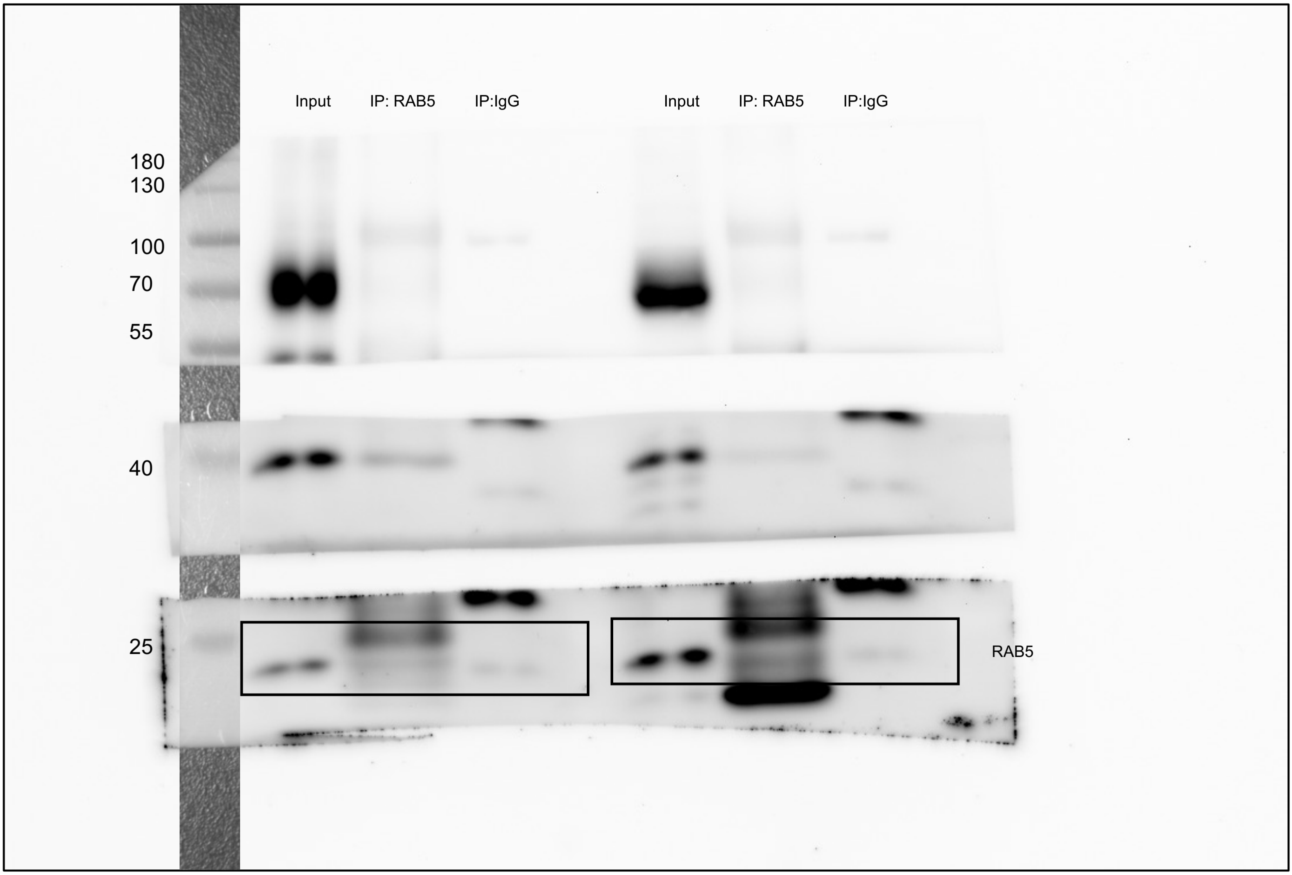

Fig. S1D (GLUT10/GFP)

RAW IMAGES OF MARKER AND PROTEIN BLOTS

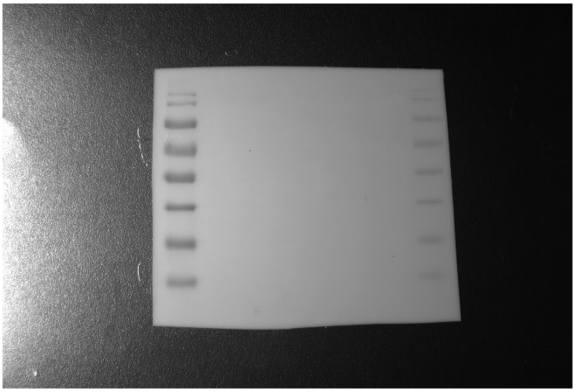

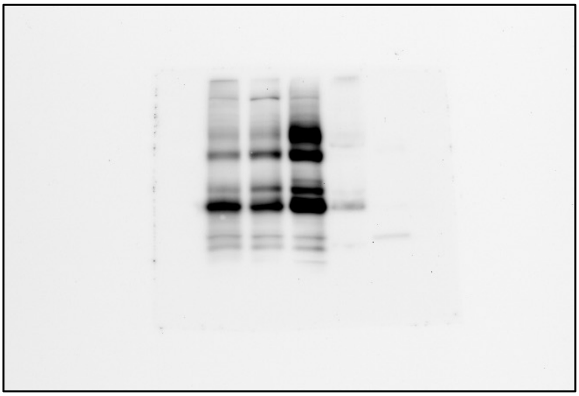

MERGED IMAGES OF MARKER AND PROTEIN

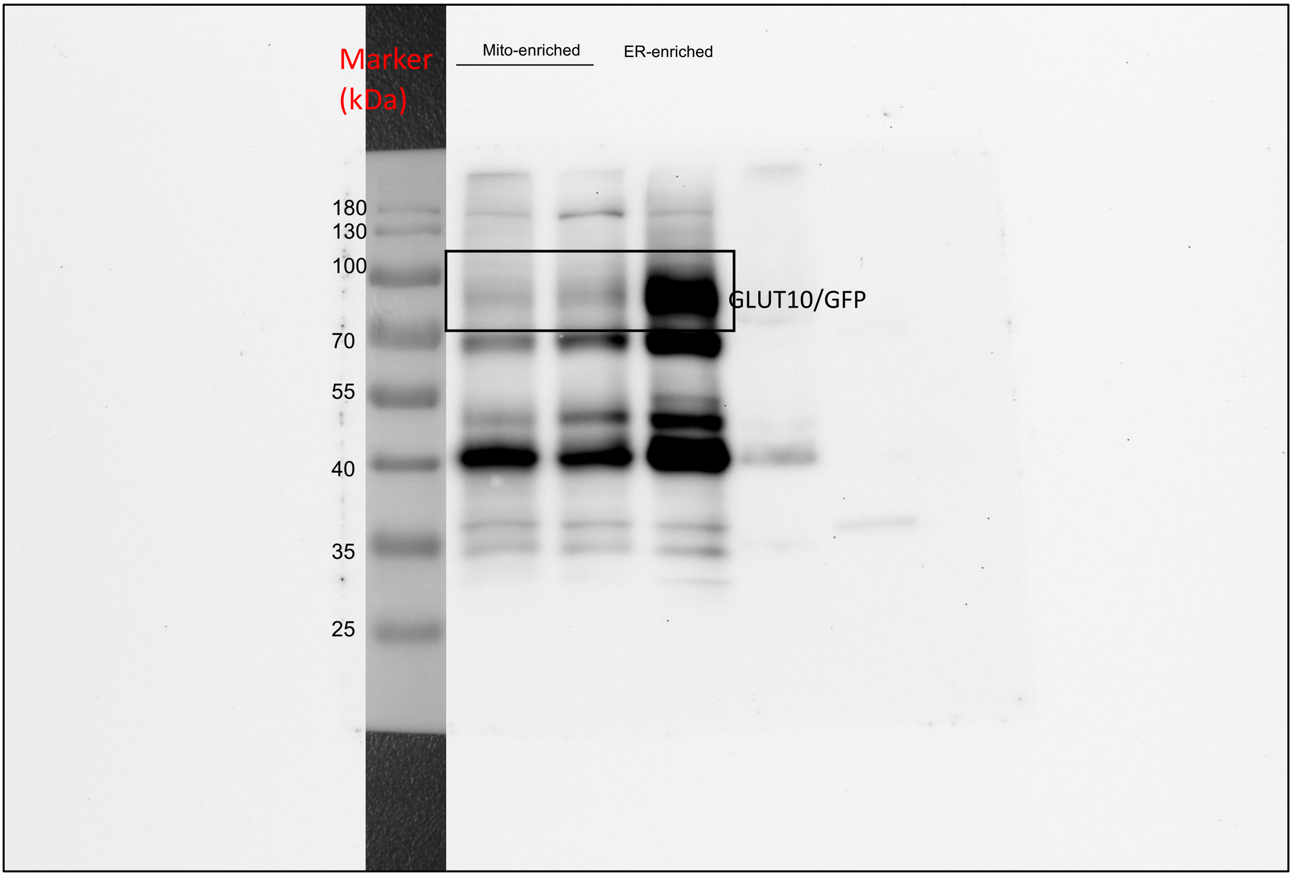

Fig. S1D/TIM 50

RAW IMAGES OF MARKER AND PROTEIN BLOTS

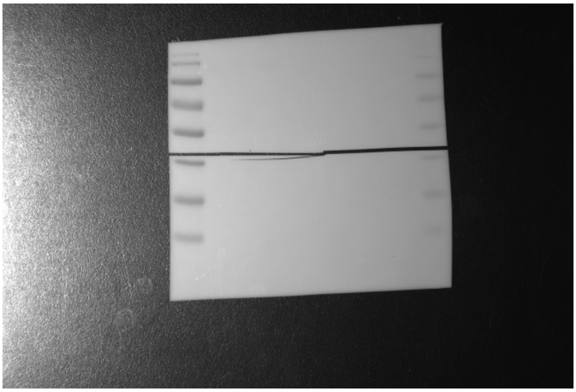

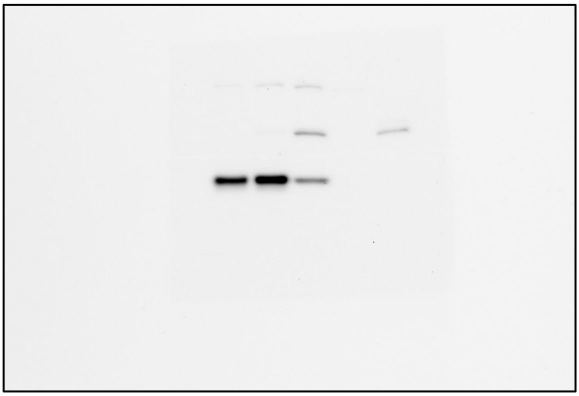

MERGED IMAGES OF MARKER AND PROTEIN

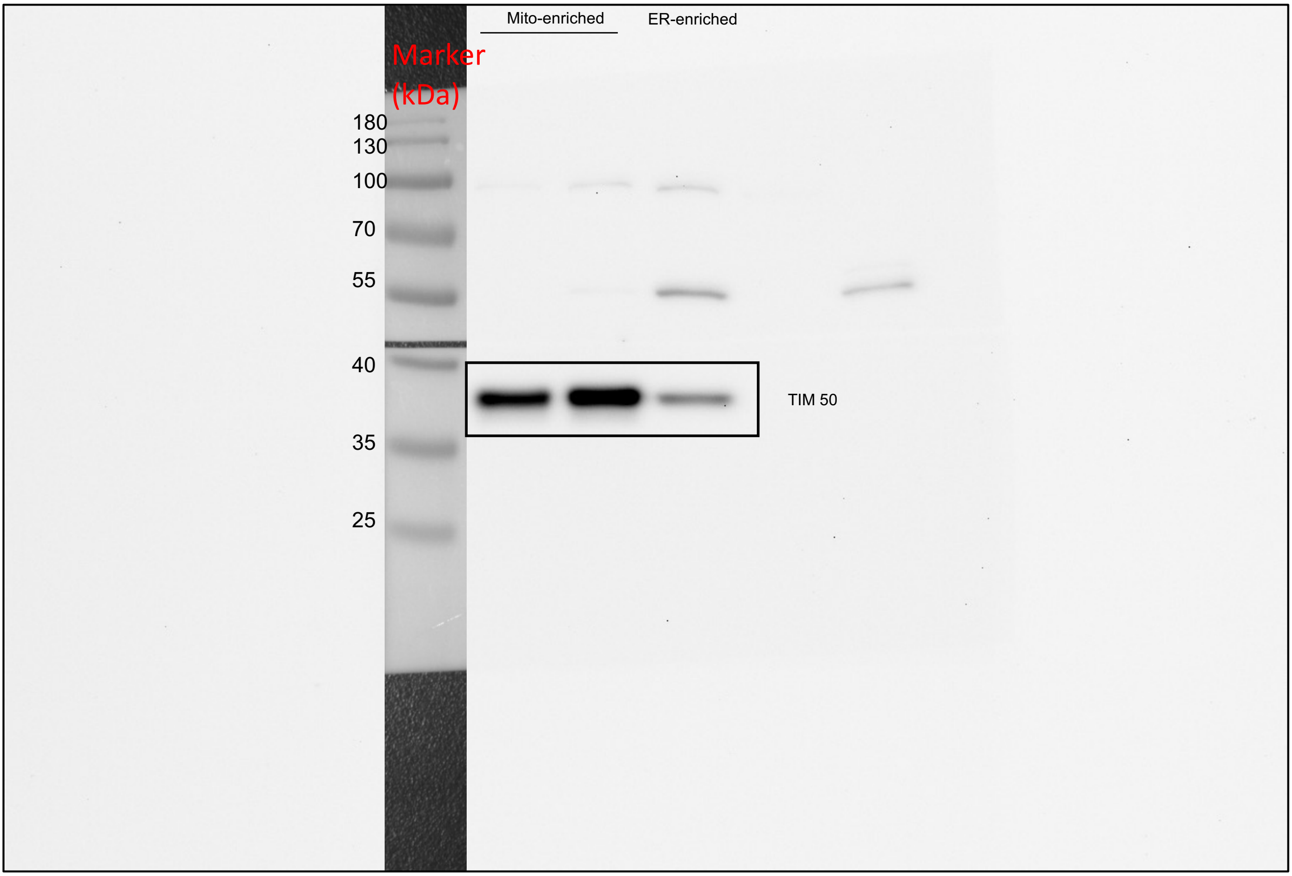

Fig. S1D/CRT, Calreticulin

RAW IMAGES OF MARKER AND PROTEIN BLOTS

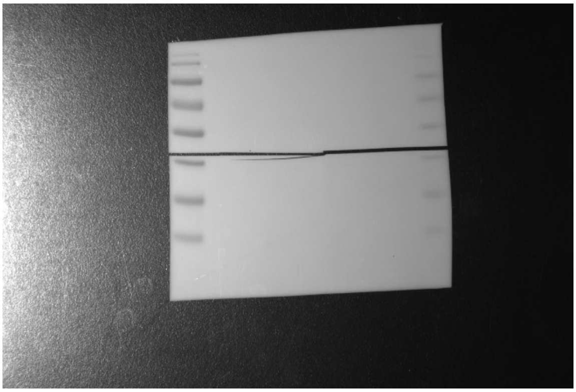

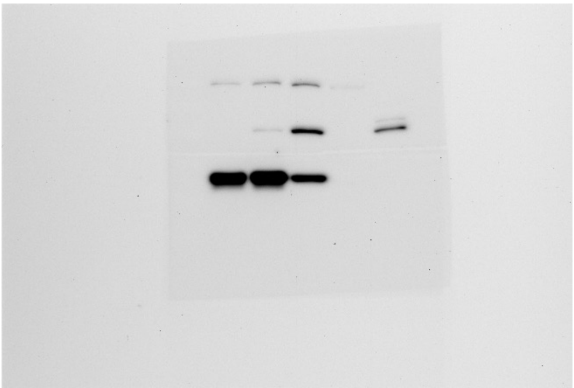

MERGED IMAGES OF MARKER AND PROTEIN

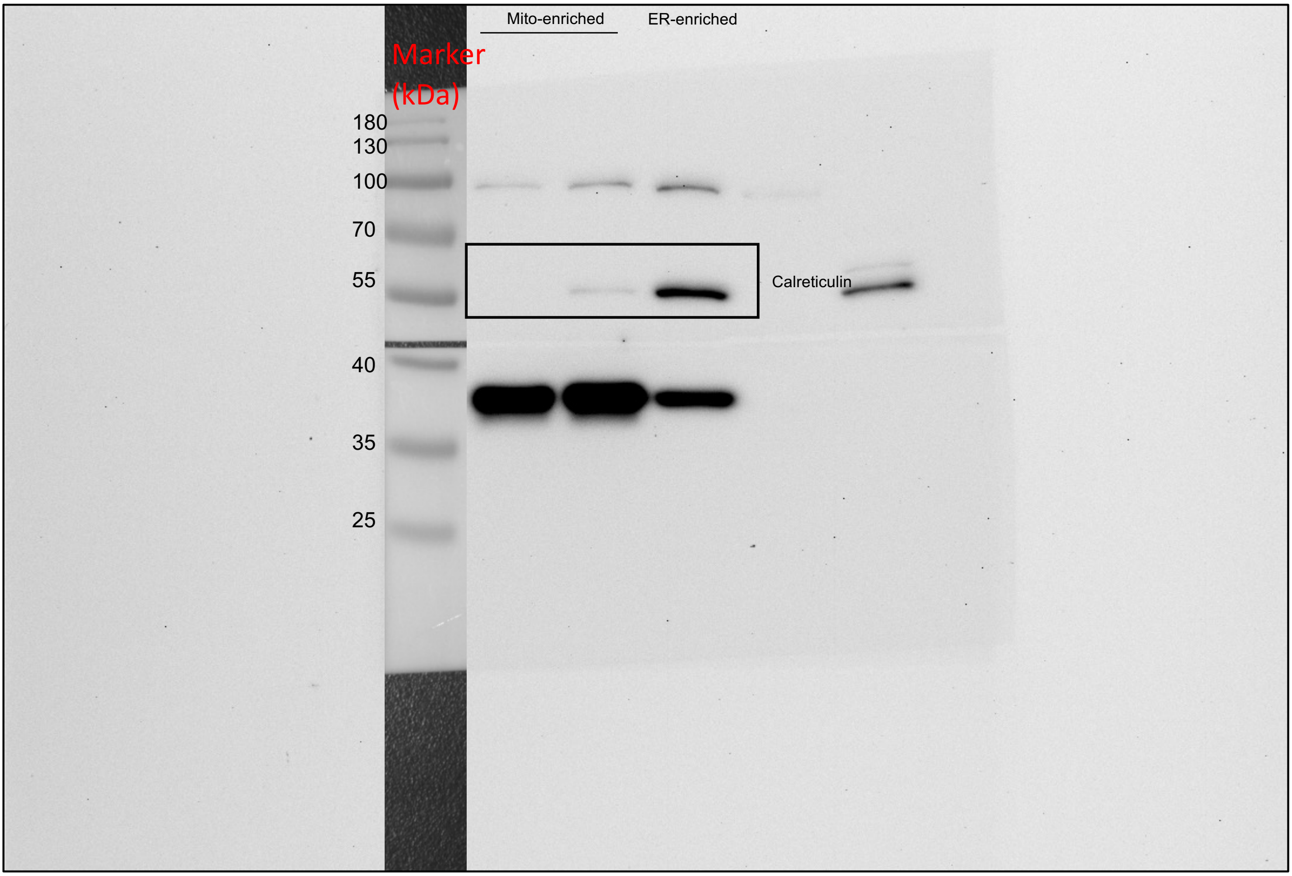

Fig. S1E/ GLUT10

RAW IMAGES OF MARKER AND PROTEIN BLOTS

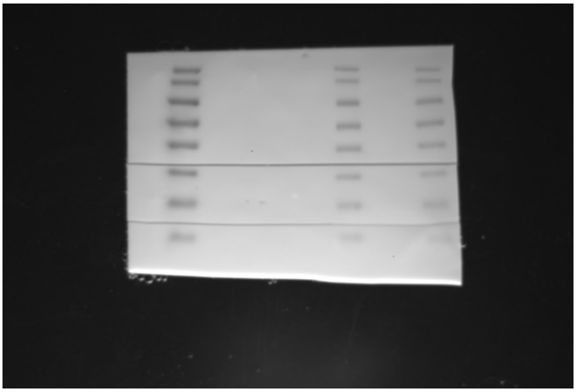

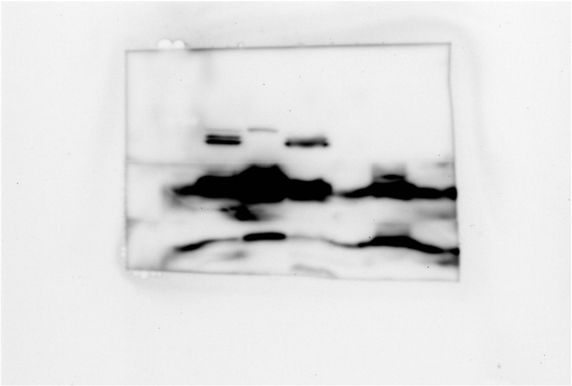

MERGED IMAGES OF MARKER AND PROTEIN

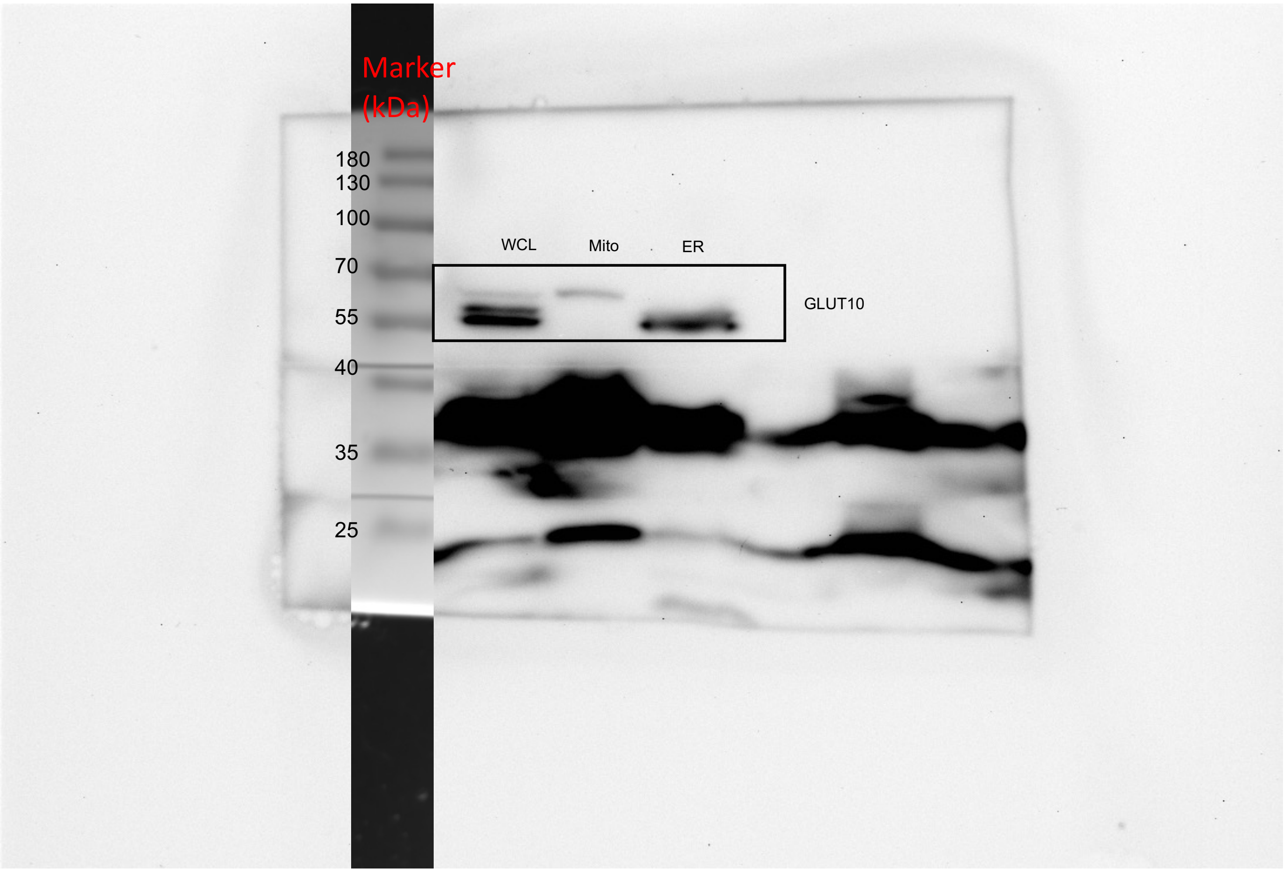

Fig. S1E/ TIM 50

RAW IMAGES OF MARKER AND PROTEIN BLOTS

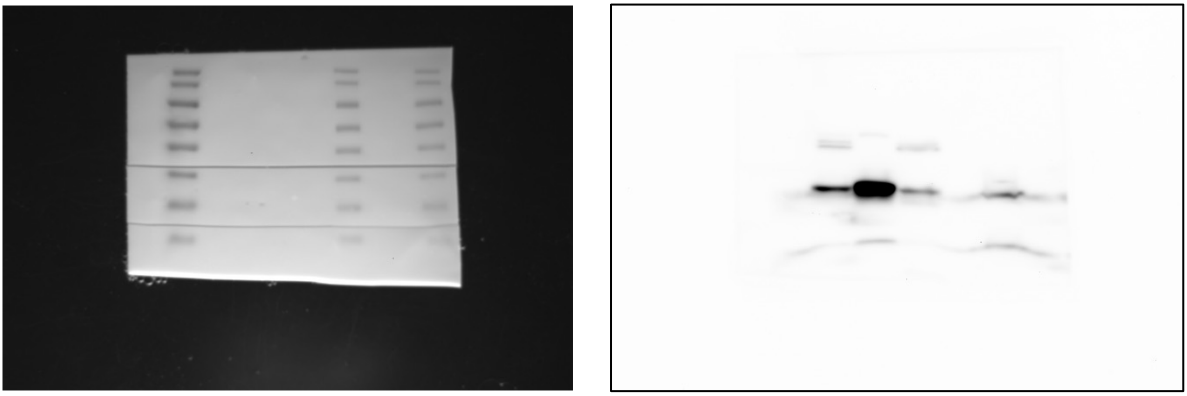

MERGED IMAGES OF MARKER AND PROTEIN

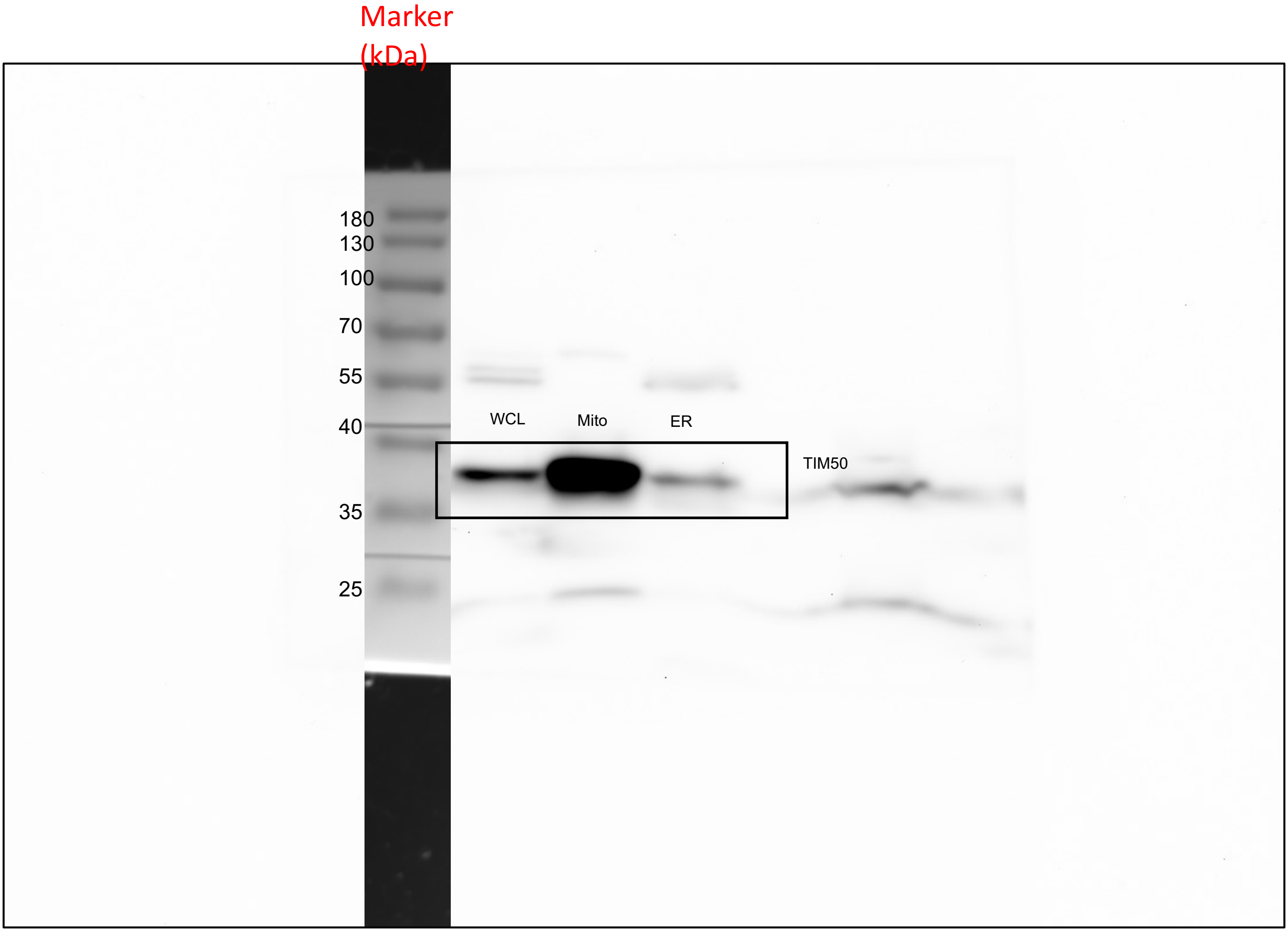

Fig. S1E/ KDEL

RAW IMAGES OF MARKER AND PROTEIN BLOTS

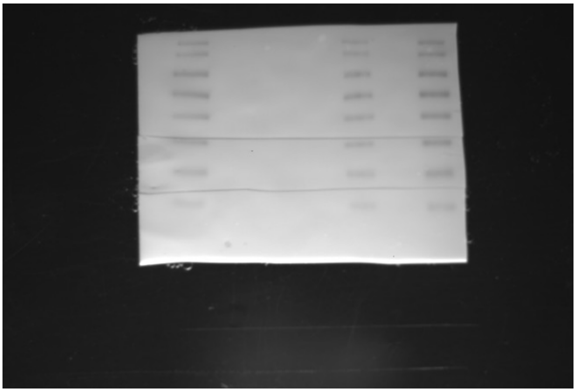

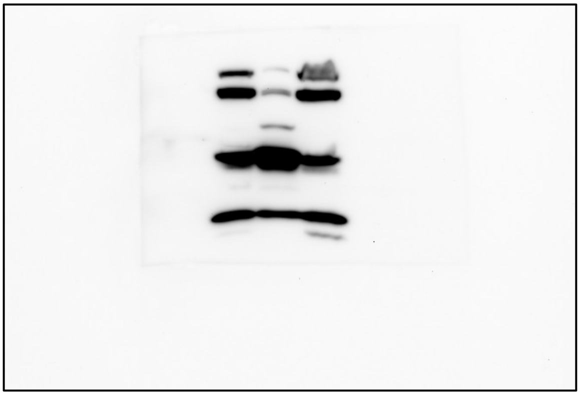

MERGED IMAGES OF MARKER AND PROTEIN

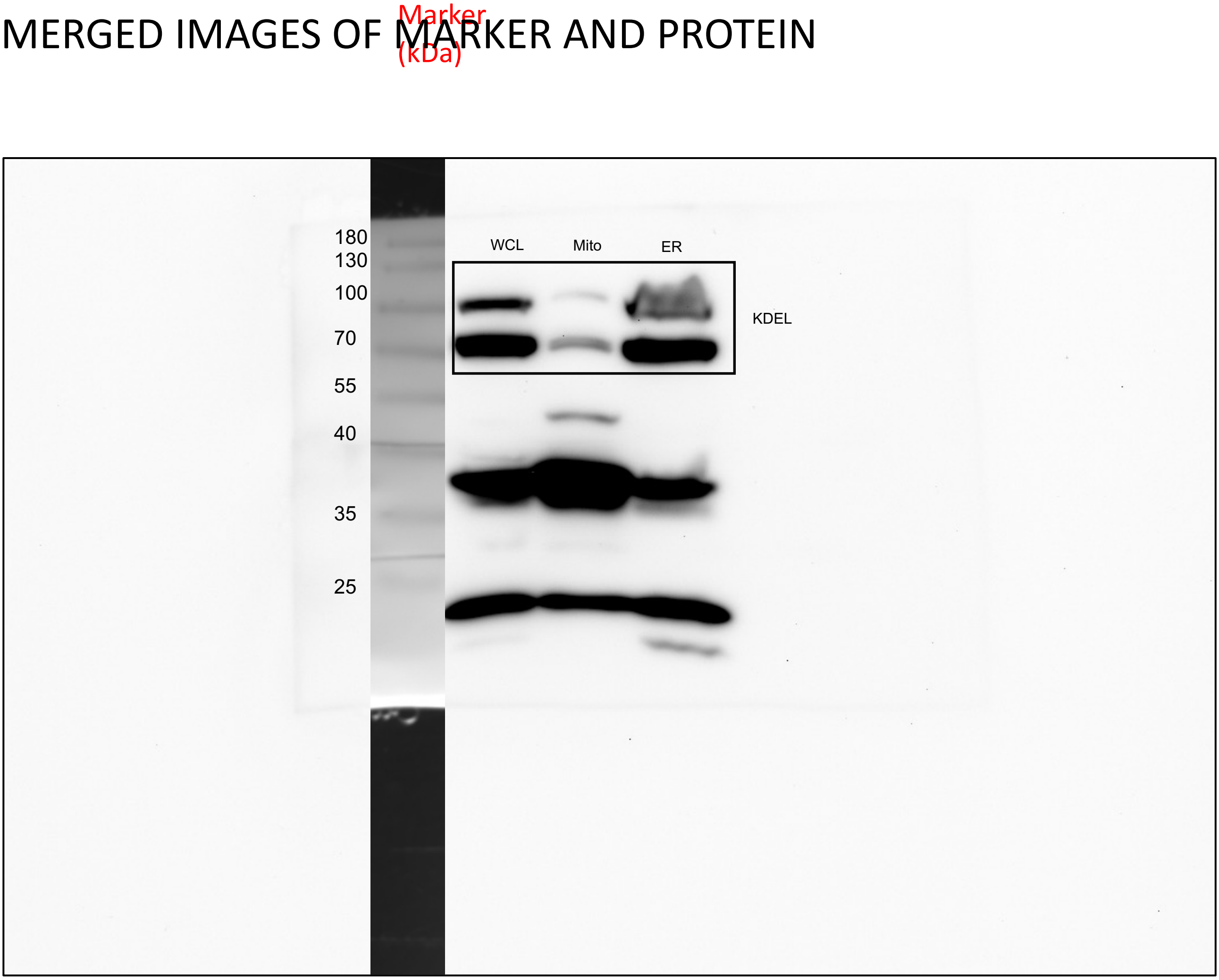

Fig. S1E/ RAB5

RAW IMAGES OF MARKER AND PROTEIN BLOTS

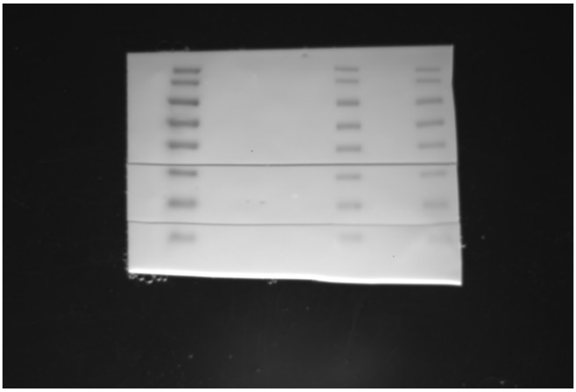

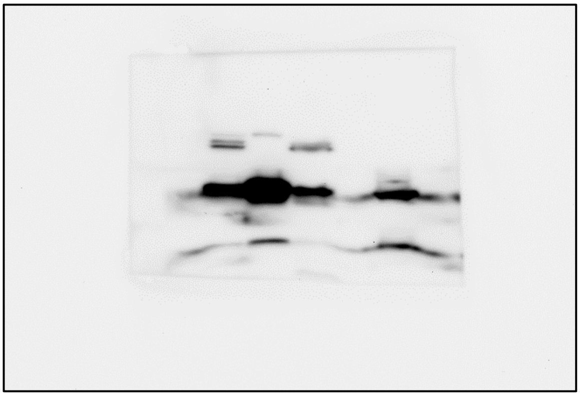

MERGED IMAGES OF MARKER AND PROTEIN

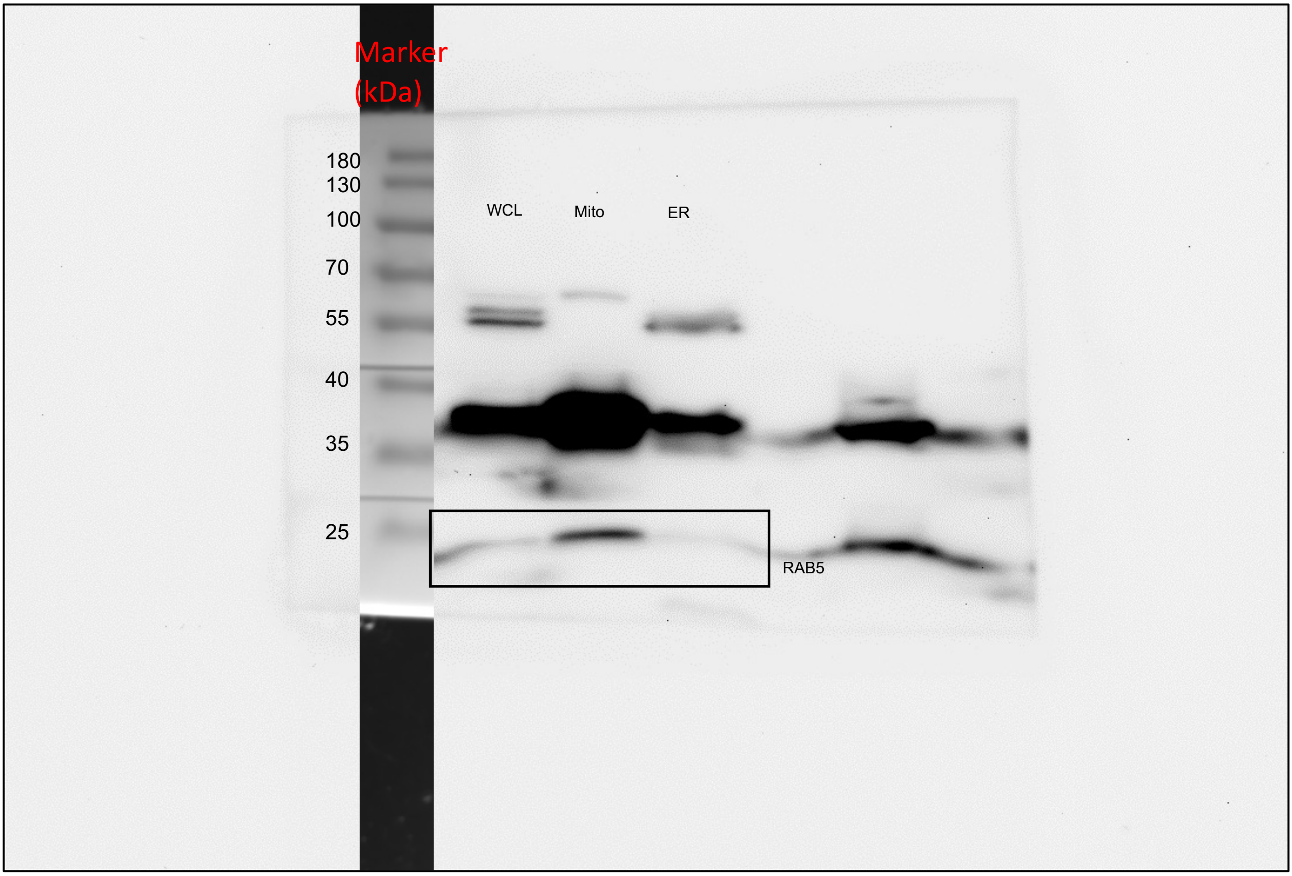

Fig. S1E/ RAB7

RAW IMAGES OF MARKER AND PROTEIN BLOTS

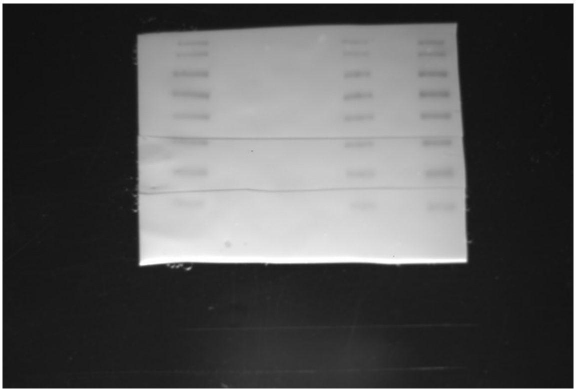

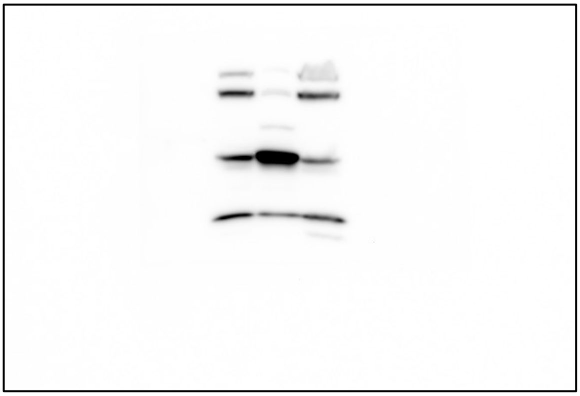

MERGED IMAGES OF MARKER AND PROTEIN

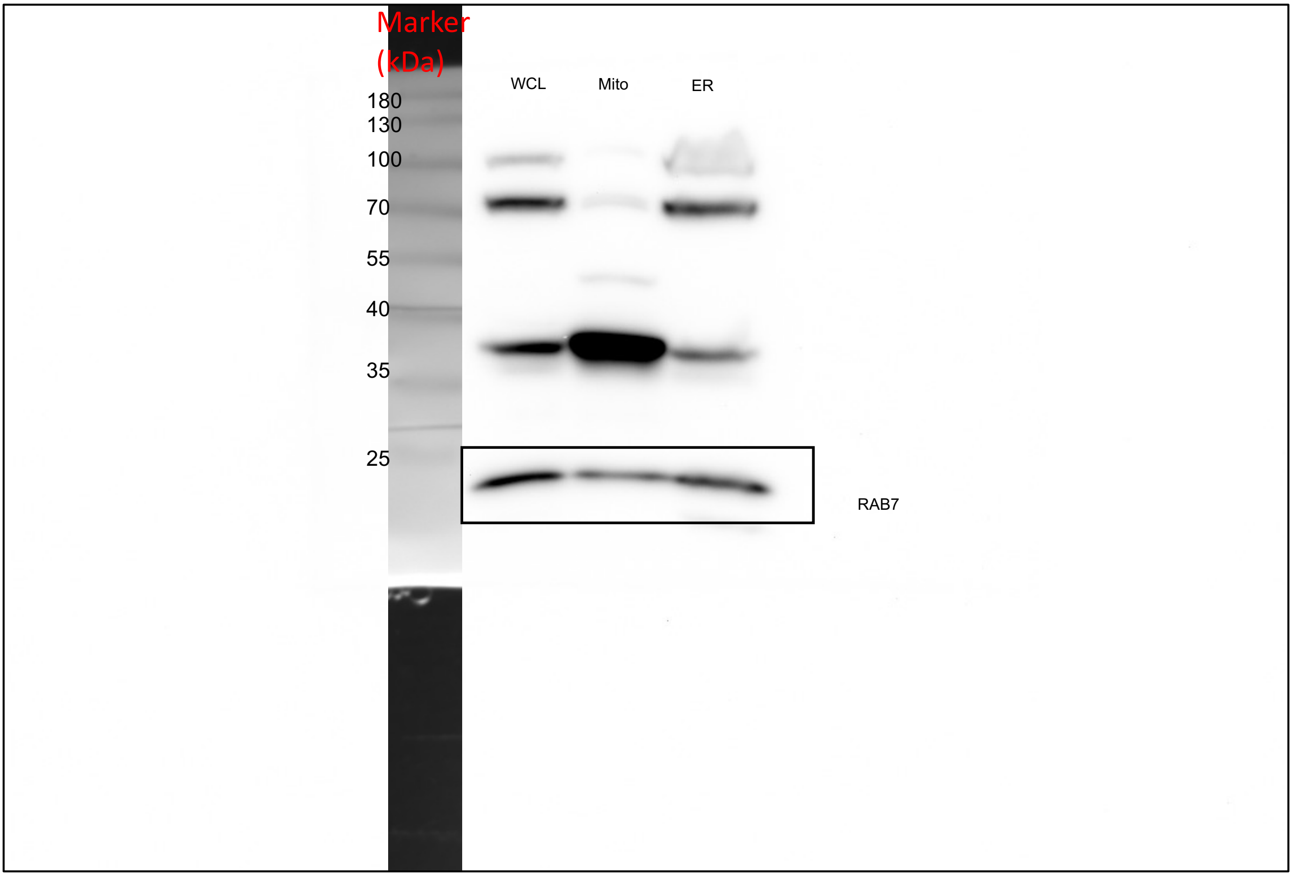

Fig. S6

RAW IMAGES OF MARKER AND PROTEIN BLOTS

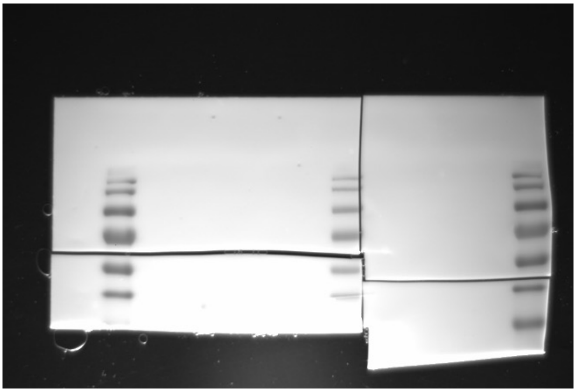

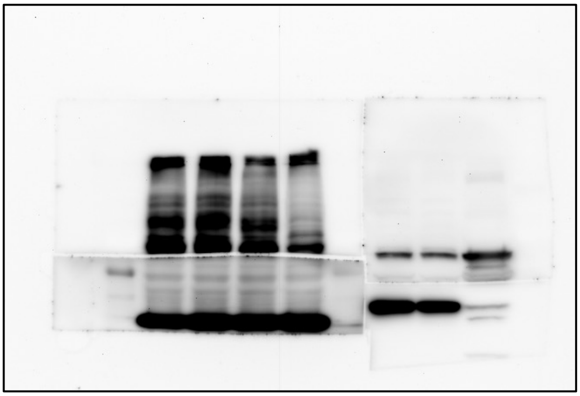

MERGED IMAGES OF MARKER AND PROTEIN

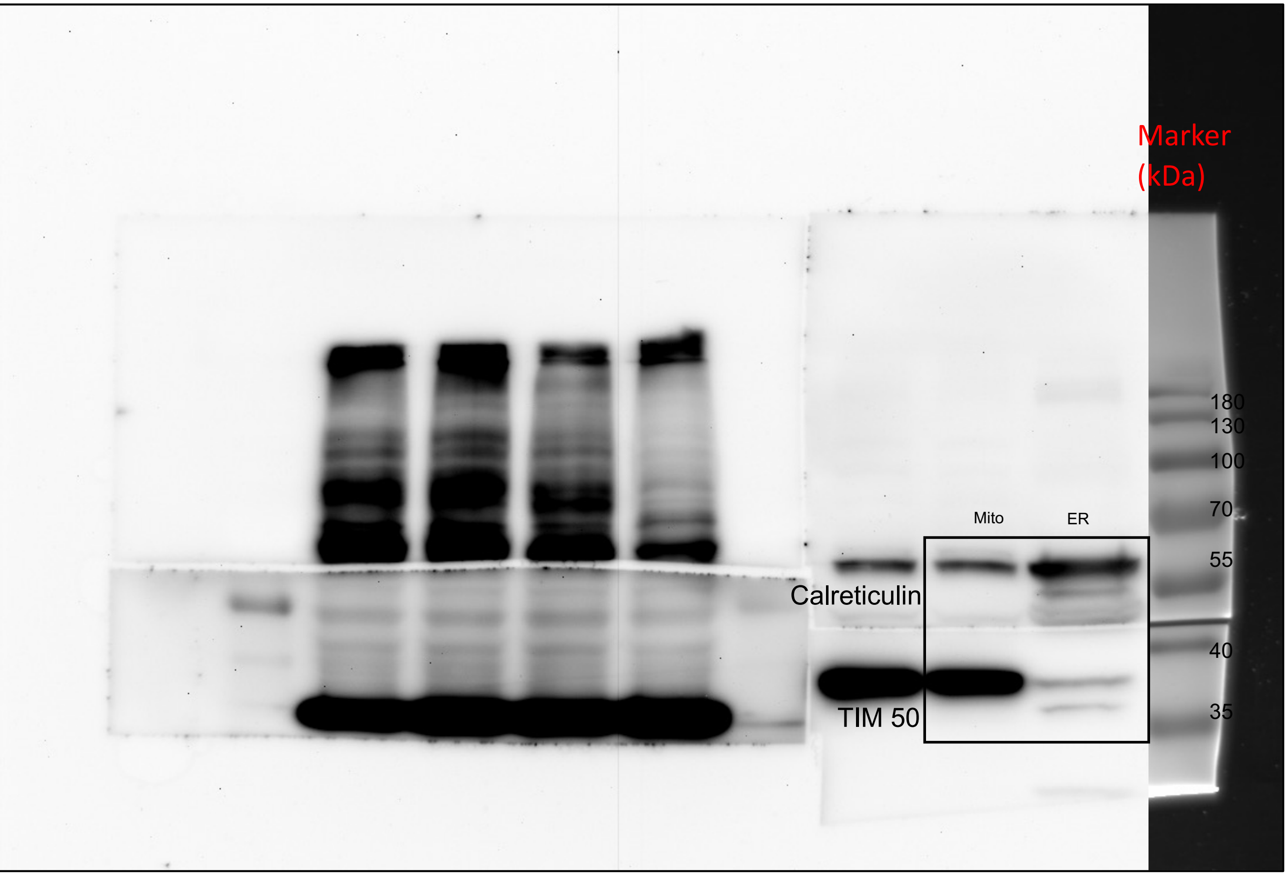

Figure S10 A/ RAB7

RAW IMAGES OF MARKER AND PROTEIN BLOTS

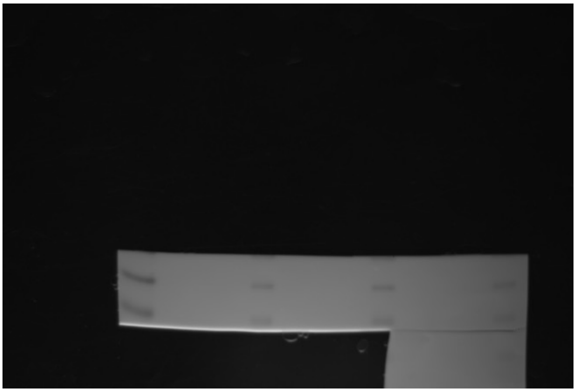

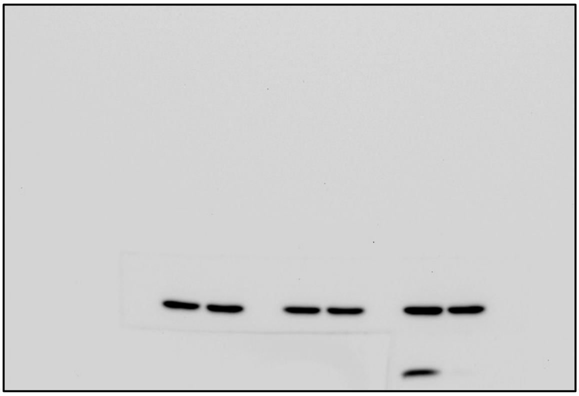

MERGED IMAGES OF MARKER AND PROTEIN

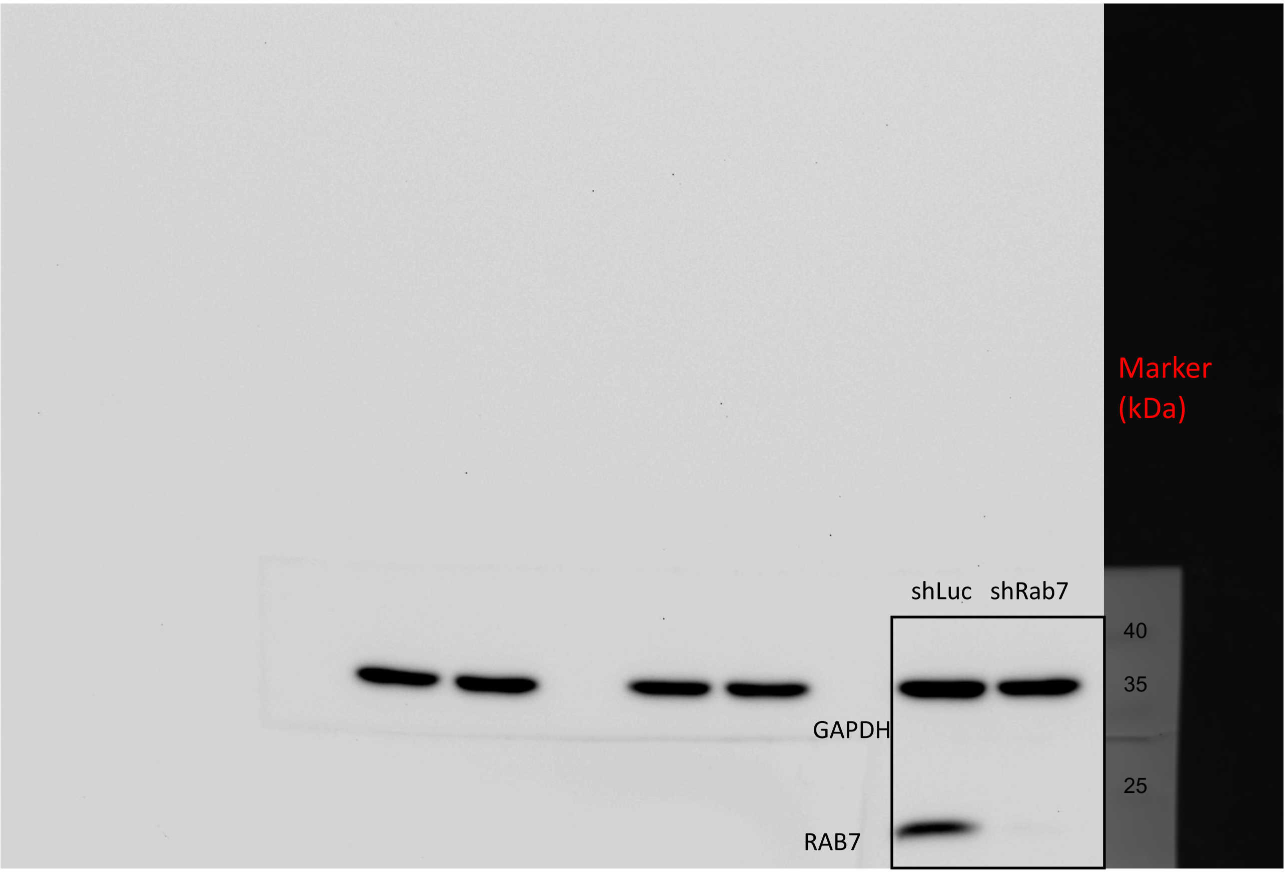

Figure S11 G (TET +6HR/ V5

RAW IMAGES OF MARKER AND PROTEIN BLOTS

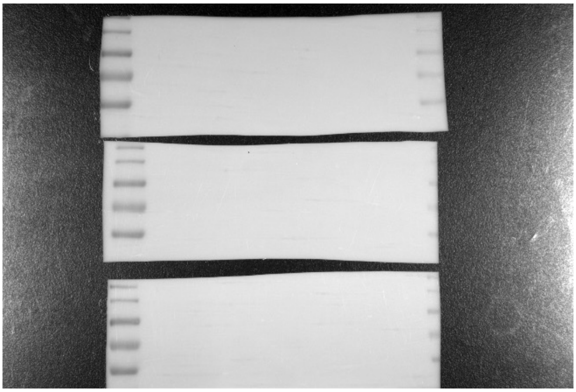

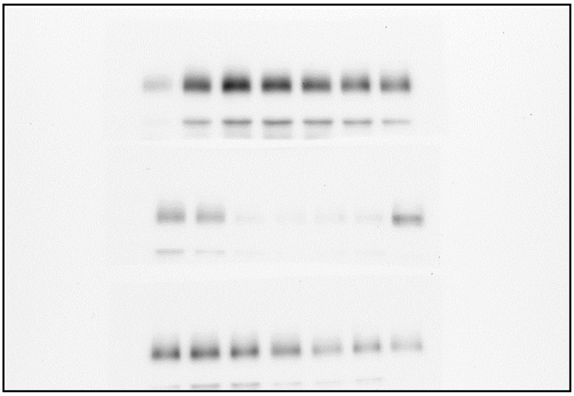

MERGED IMAGES OF MARKER AND PROTEIN

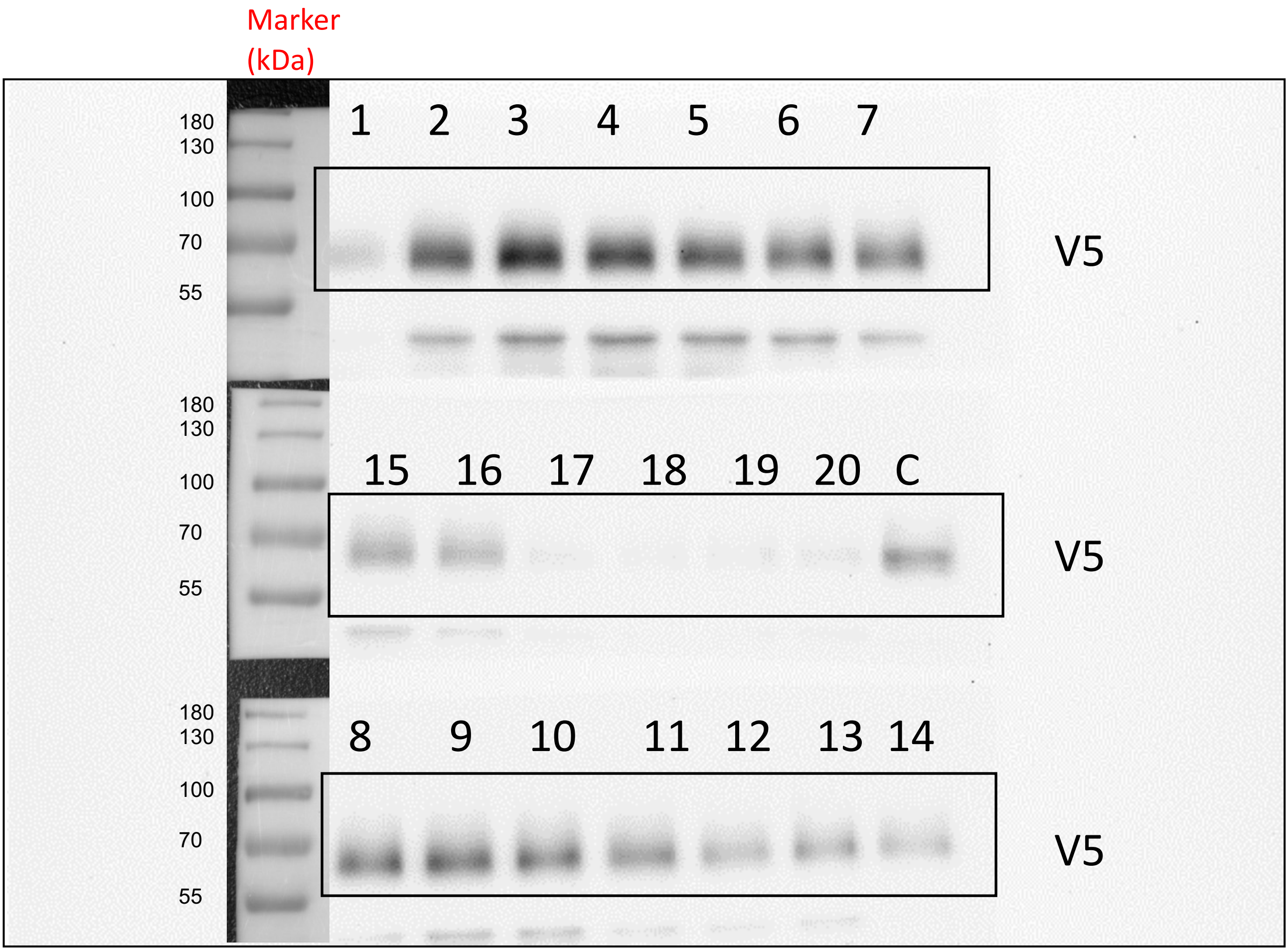

Figure S11 G (TET +6HR/TIM 50)

RAW IMAGES OF MARKER AND PROTEIN BLOTS

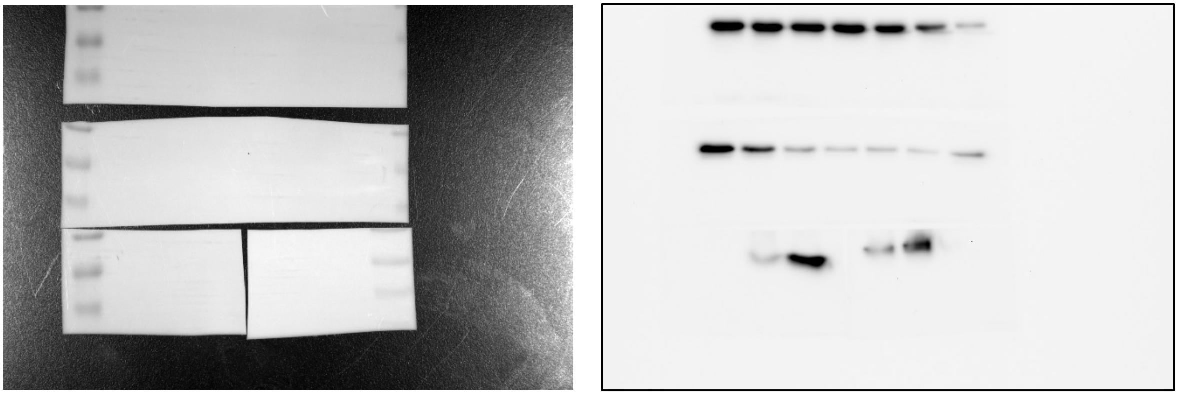

MERGED IMAGES OF MARKER AND PROTEIN

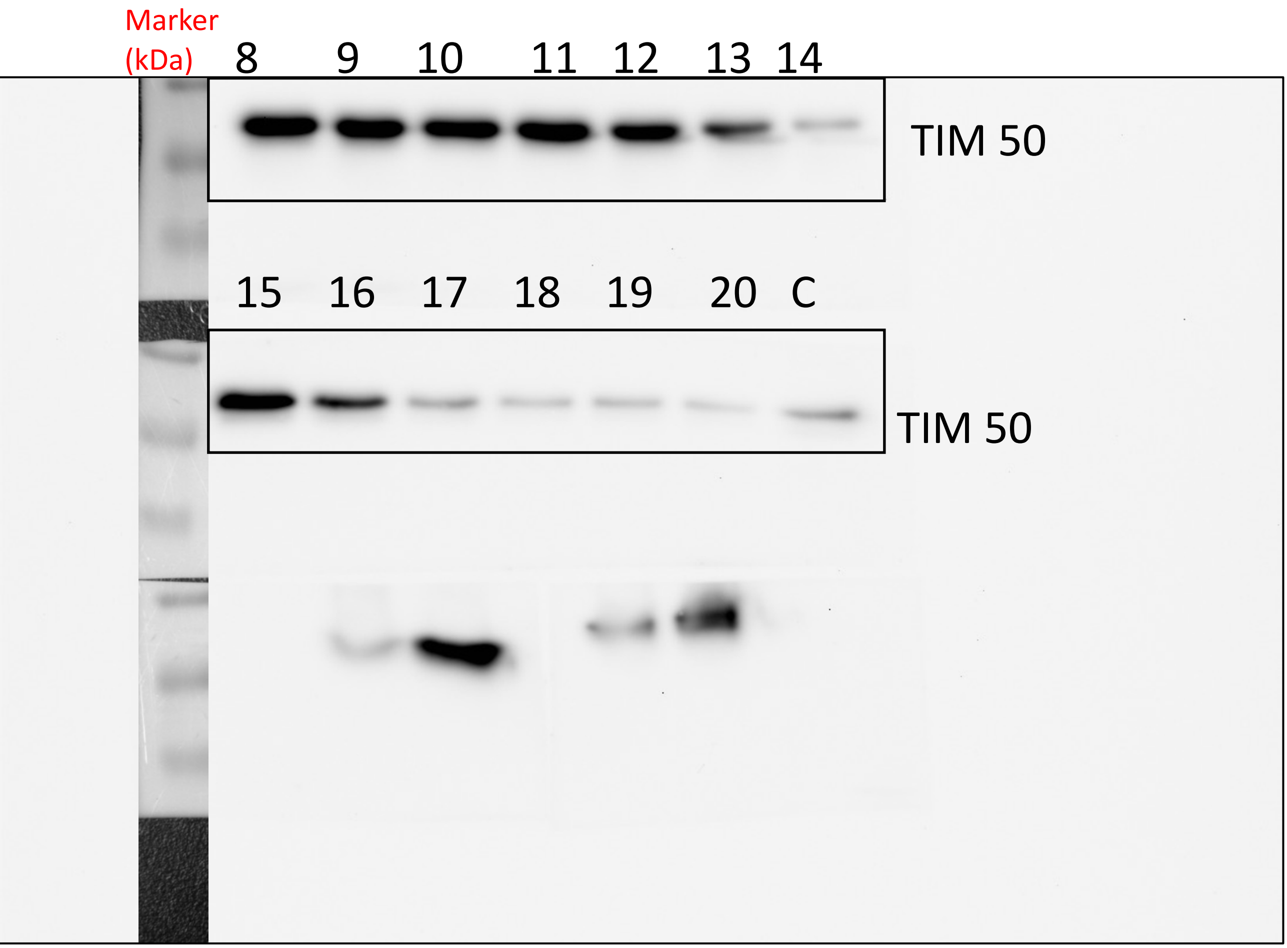

Figure S11 G (TET +6HR/TIM 50)

RAW IMAGES OF PROTEIN BLOTS with molecular weight marked

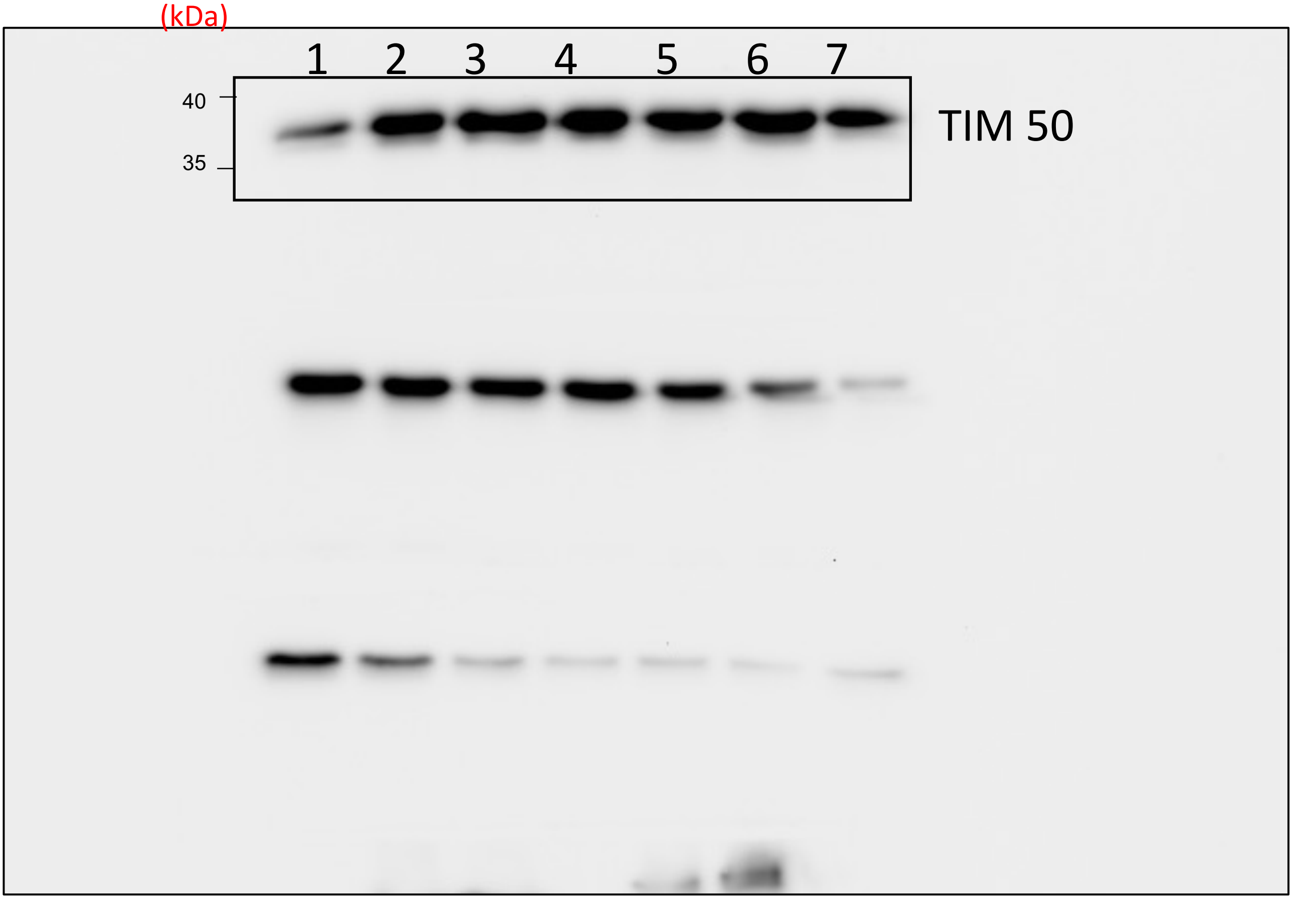

Figure S11 G (TET +6HR/ RAB5)

RAW IMAGES OF MARKER AND PROTEIN BLOTS

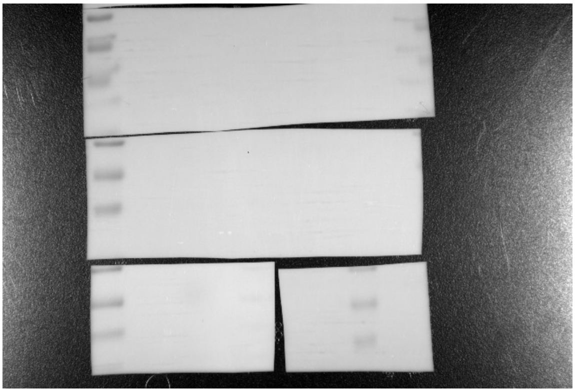

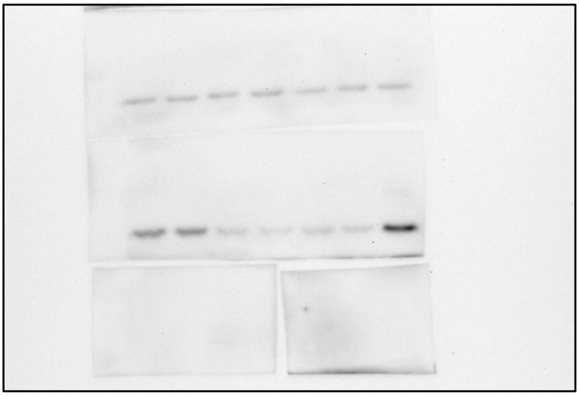

MERGED IMAGES OF MARKER AND PROTEIN

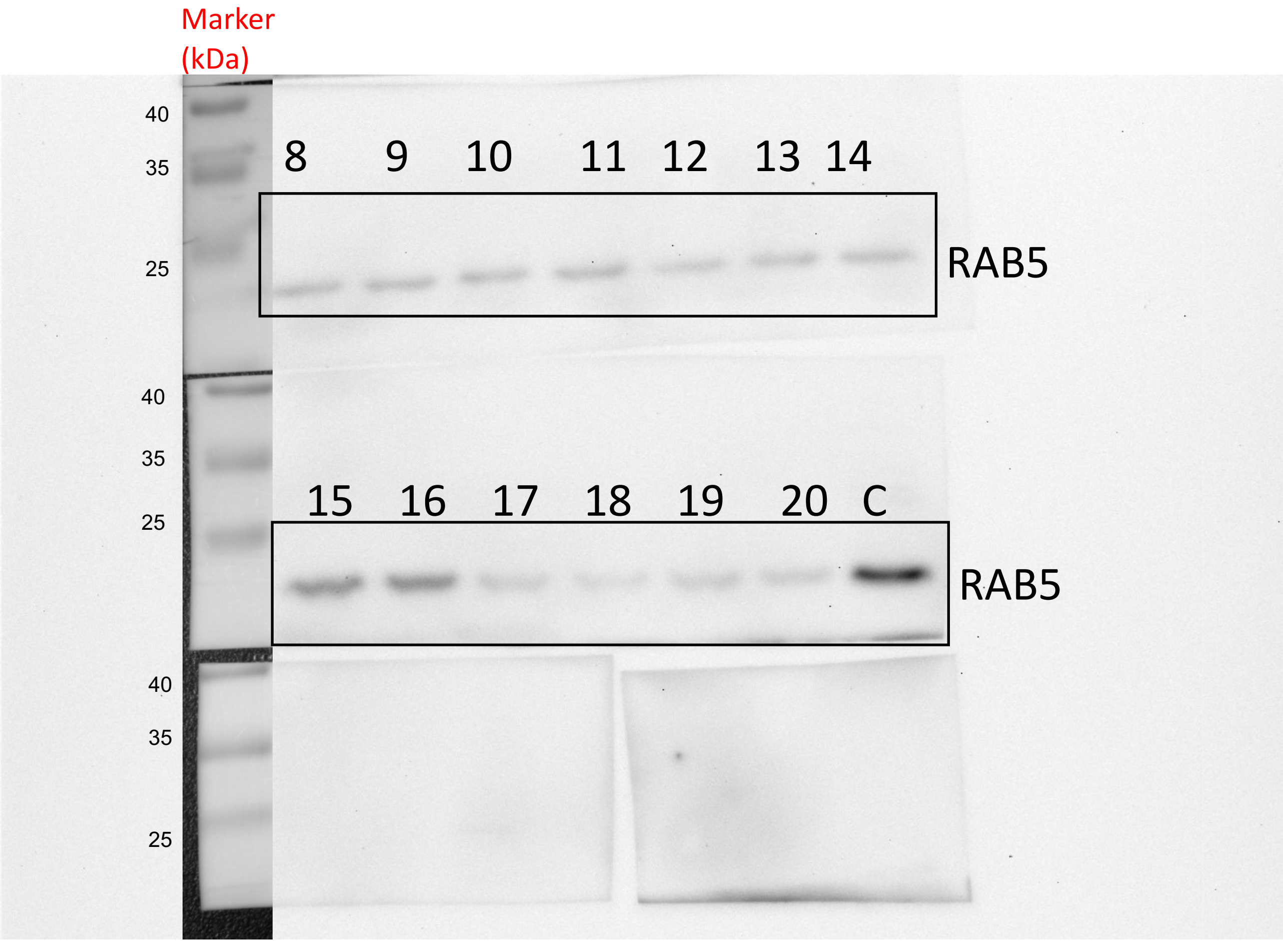

Figure S11 G (TET +6HR/ RAB5)

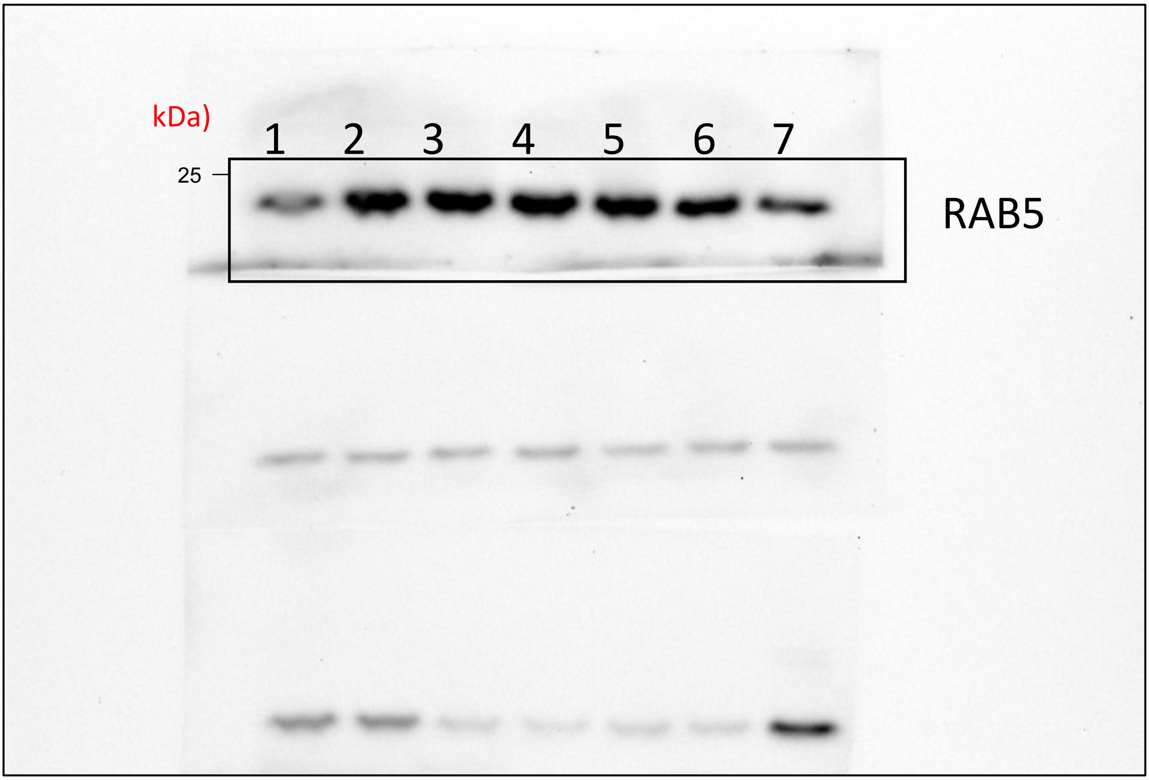

Figure S11 G (TET +6HR/ GM130 and KDEL

RAW IMAGES OF MARKER AND PROTEIN BLOTS

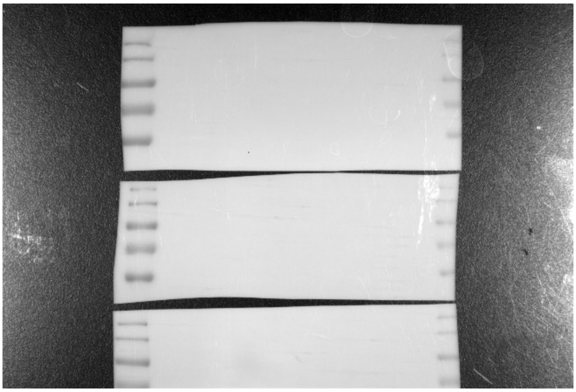

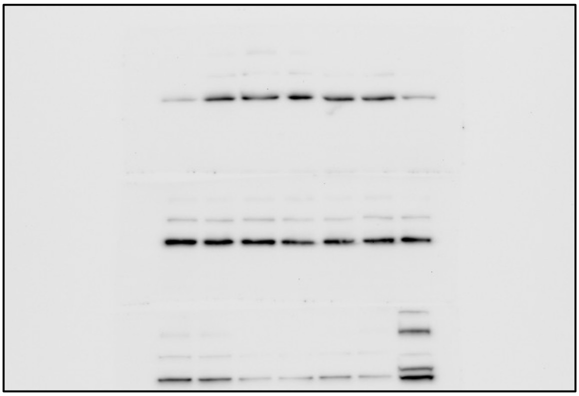

MERGED IMAGES OF MARKER AND PROTEIN

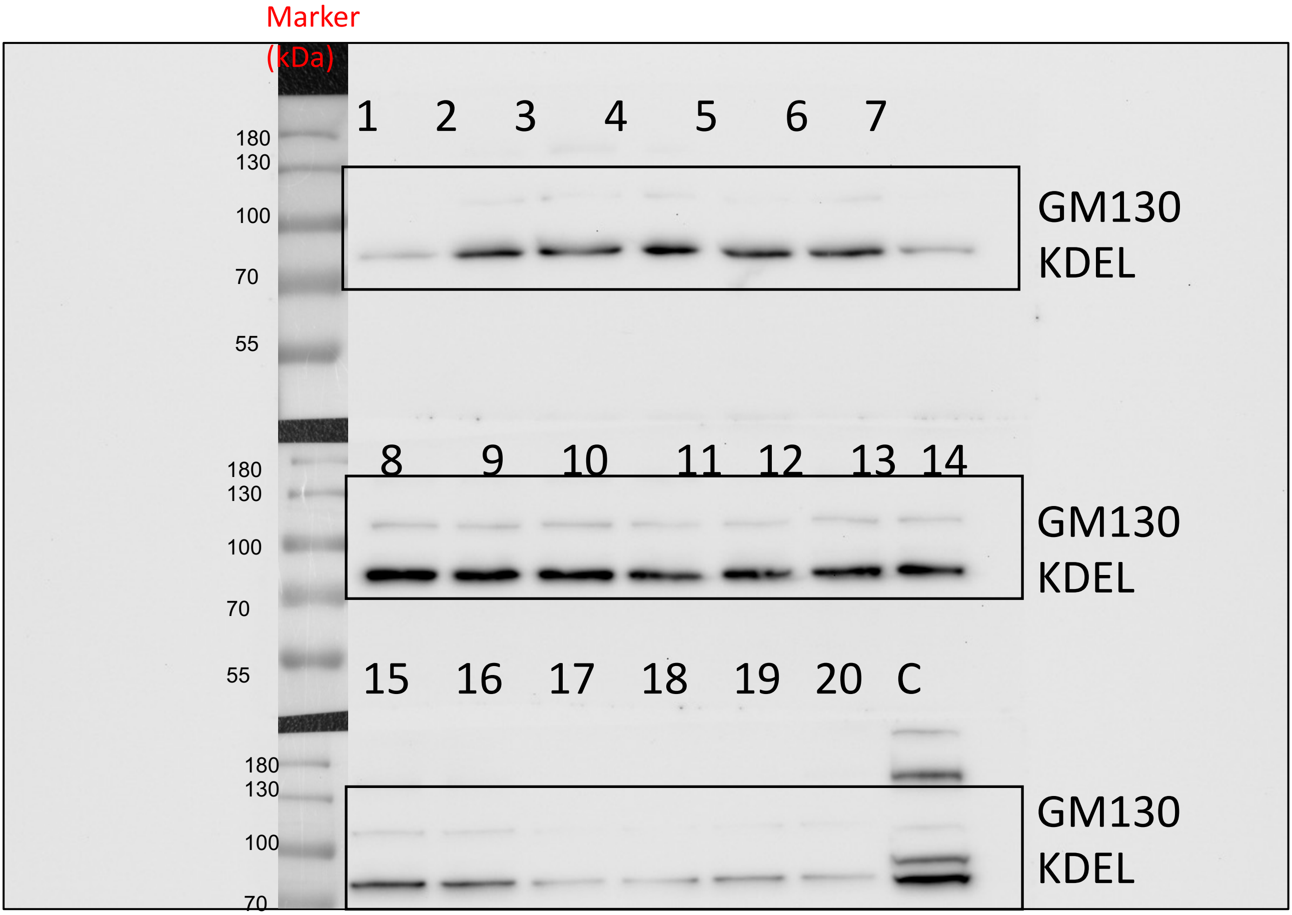

Figure S11 G (TET +24HR/ V5)

RAW IMAGES OF MARKER AND PROTEIN BLOTS

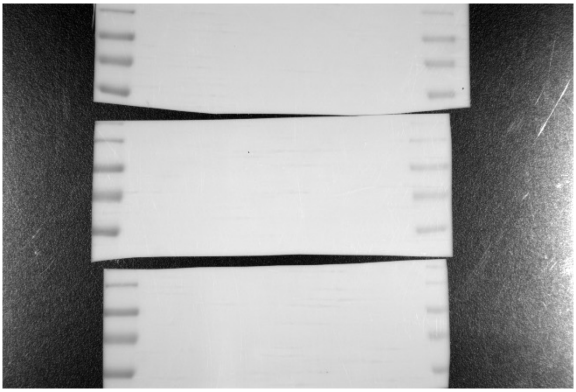

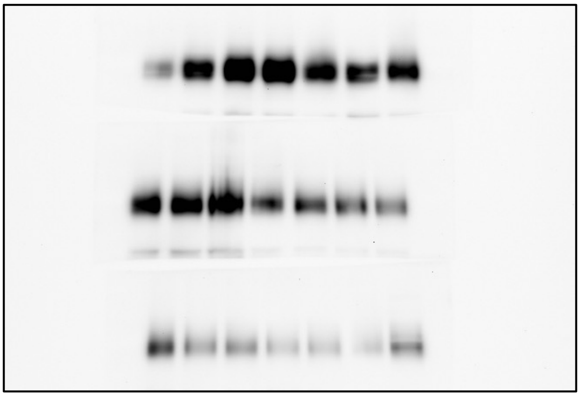

MERGED IMAGES OF MARKER AND PROTEIN

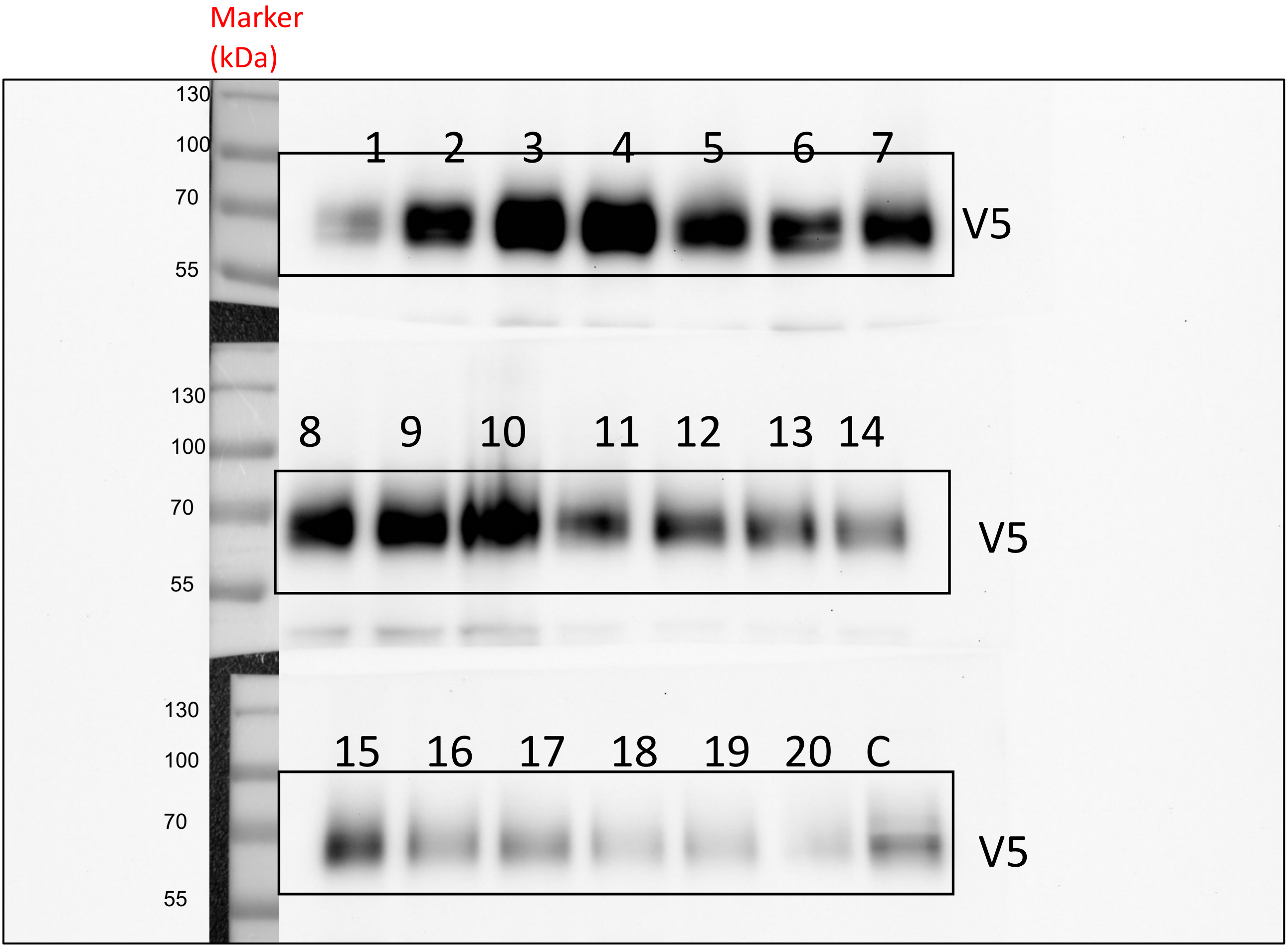

Figure S11 G (TET +24HR/ TIM50)

RAW IMAGES OF MARKER AND PROTEIN BLOTS

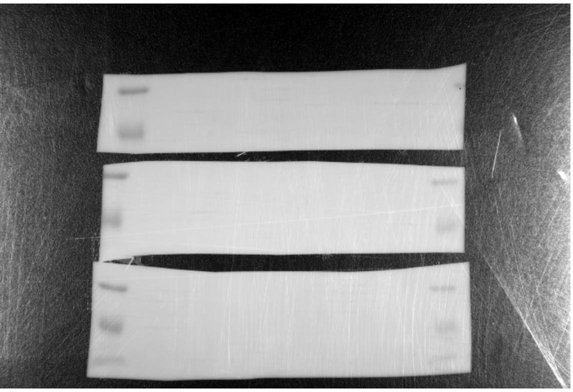

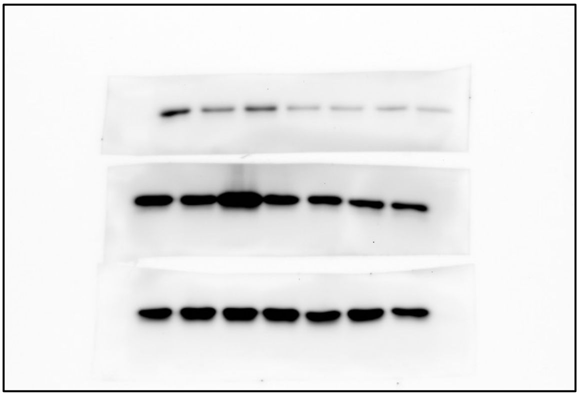

MERGED IMAGES OF MARKER AND PROTEIN

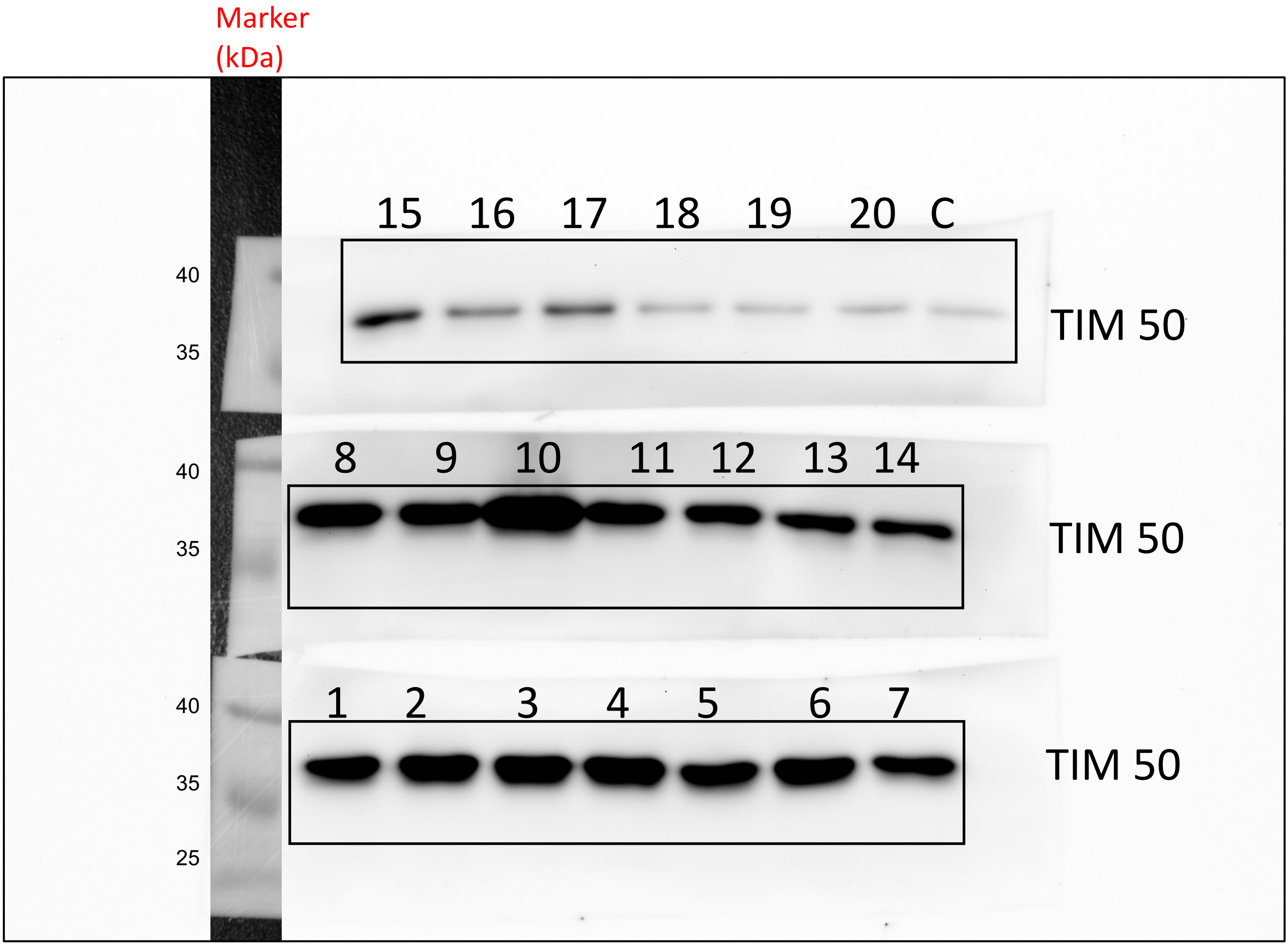

Figure S11 G (TET +24HR/ RAB5)

RAW IMAGES OF MARKER AND PROTEIN BLOTS

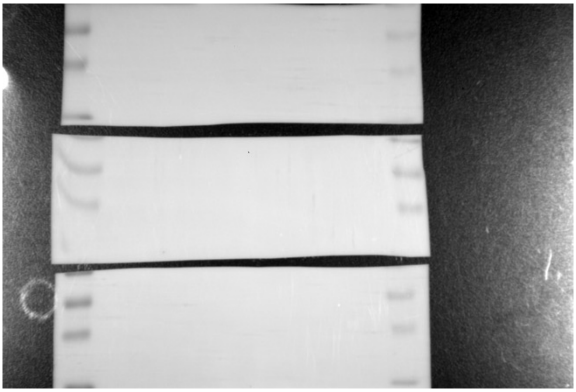

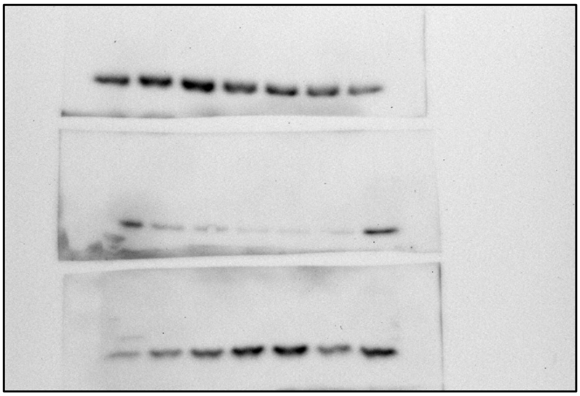

MERGED IMAGES OF MARKER AND PROTEIN

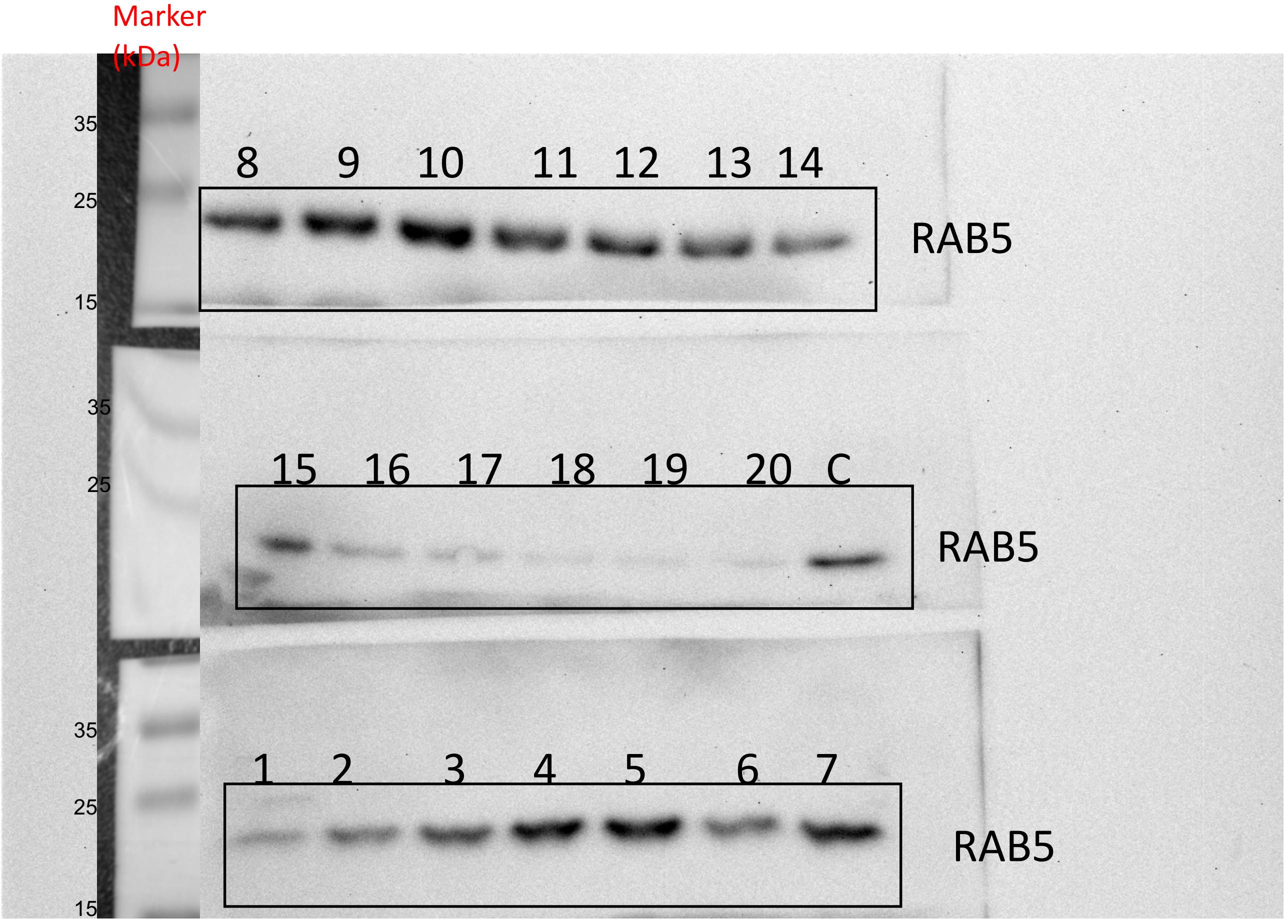

Figure S11 G (TET +24HR/ GM130 and KDEL)

RAW IMAGES OF MARKER AND PROTEIN BLOTS

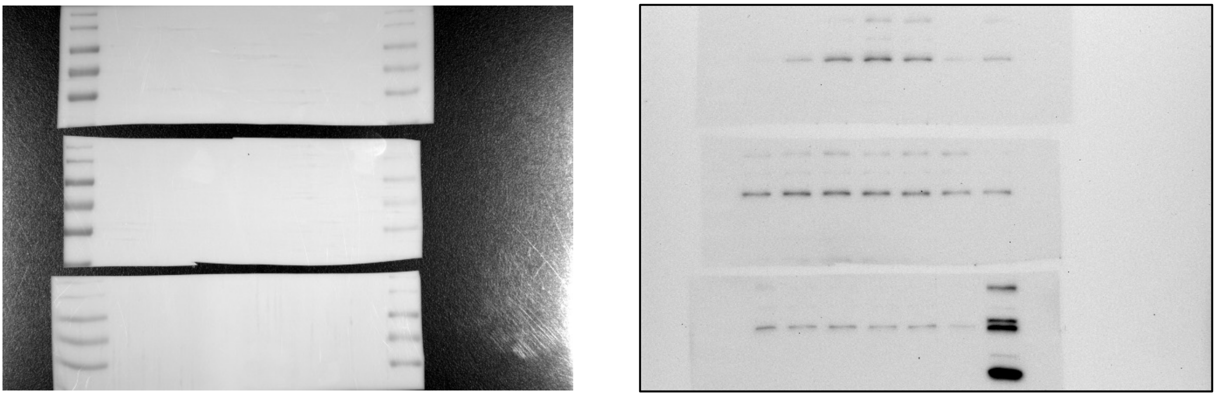

MERGED IMAGES OF MARKER AND PROTEIN

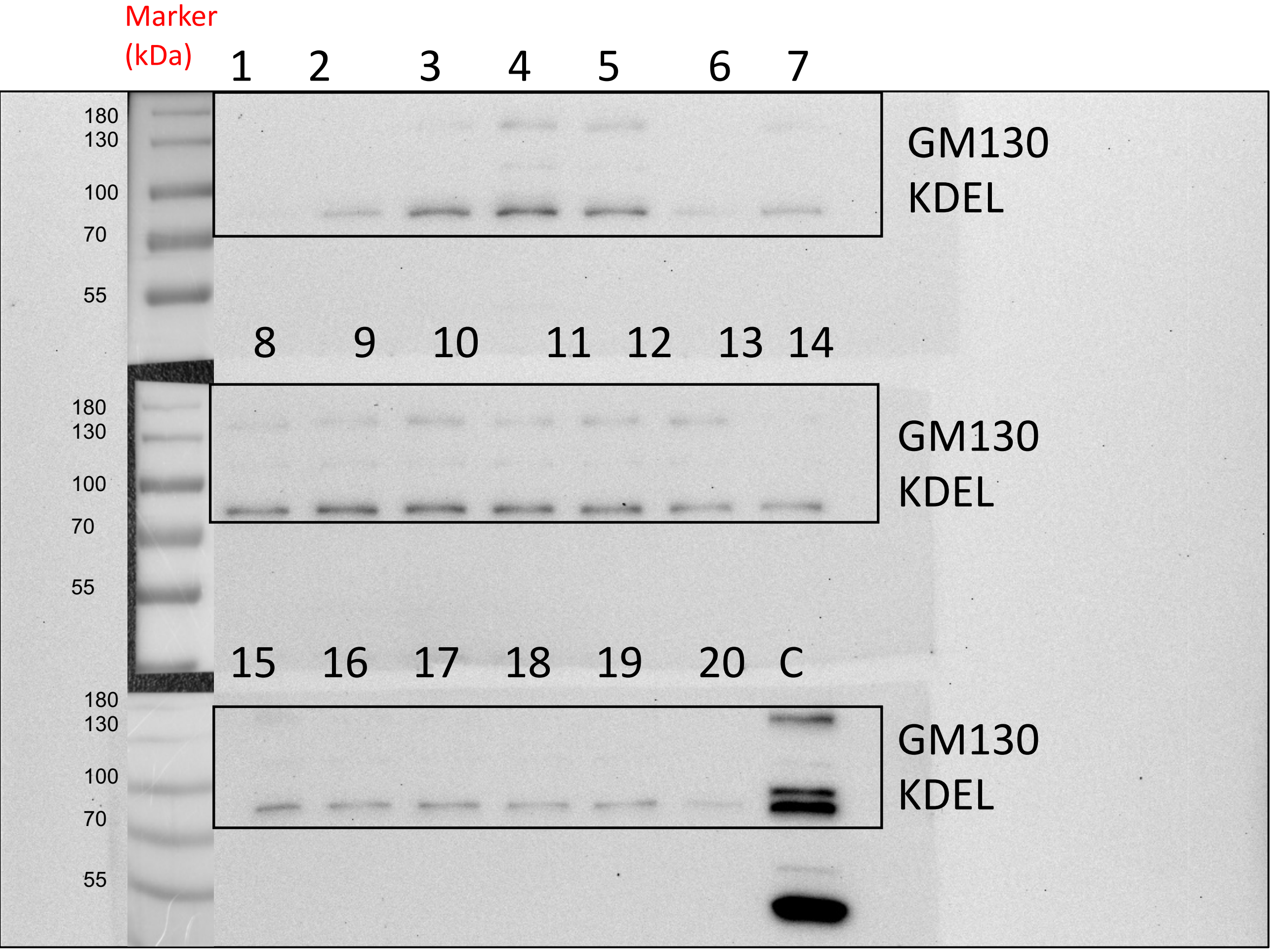

Supplement: Document S2. 20260325 WB and EM UNCROPPED IMAGES with replicates [file mmc2.pdf]
